# Genome-Scale Metabolic Modeling Reveals Metabolic Alterations of Multidrug-Resistant *Acinetobacter baumannii* in a Murine Bloodstream Infection Model

**DOI:** 10.3390/microorganisms8111793

**Published:** 2020-11-16

**Authors:** Jinxin Zhao, Yan Zhu, Jiru Han, Yu-Wei Lin, Michael Aichem, Jiping Wang, Ke Chen, Tony Velkov, Falk Schreiber, Jian Li

**Affiliations:** 1 Infection and Immunity Program, Department of Microbiology, Biomedicine Discovery Institute, Monash University, Clayton, VIC 3800, Australia; jinxin.zhao@monash.edu (J.Z.); Yu-Wei.Lin@monash.edu (Y.-W.L.); Jiping.Wang@monash.edu (J.W.); Ke.Chen@monash.edu (K.C.); 2 Population Health and Immunity Division, The Walter and Eliza Hall Institute of Medical Research, Parkville, VIC 3052, Australia; han.ji@wehi.edu.au; 3 Department of Computer and Information Science, University of Konstanz, 78457 Konstanz, Germany; michael.aichem@uni-konstanz.de (M.A.); falk.schreiber@uni-konstanz.de (F.S.); 4 Department of Pharmacology and Therapeutics, University of Melbourne, Melbourne, VIC 3010, Australia; tony.velkov@unimelb.edu.au

**Keywords:** *Acinetobacter baumannii*, genome-scale metabolic modeling, transcriptomics, bacteremia, RNA-seq

## Abstract

Multidrug-resistant (MDR) *Acinetobacter baumannii* is a critical threat to human health globally. We constructed a genome-scale metabolic model *i*AB5075 for the hypervirulent, MDR *A. baumannii* strain AB5075. Predictions of nutrient utilization and gene essentiality were validated using Biolog assay and a transposon mutant library. In vivo transcriptomics data were integrated with *i*AB5075 to elucidate bacterial metabolic responses to the host environment. *i*AB5075 contains 1530 metabolites, 2229 reactions, and 1015 genes, and demonstrated high accuracies in predicting nutrient utilization and gene essentiality. At 4 h post-infection, a total of 146 metabolic fluxes were increased and 52 were decreased compared to 2 h post-infection; these included enhanced fluxes through peptidoglycan and lipopolysaccharide biosynthesis, tricarboxylic cycle, gluconeogenesis, nucleotide and fatty acid biosynthesis, and altered fluxes in amino acid metabolism. These flux changes indicate that the induced central metabolism, energy production, and cell membrane biogenesis played key roles in establishing and enhancing *A. baumannii* bloodstream infection. This study is the first to employ genome-scale metabolic modeling to investigate *A. baumannii* infection in vivo. Our findings provide important mechanistic insights into the adaption of *A. baumannii* to the host environment and thus will contribute to the development of new therapeutic agents against this problematic pathogen.

## 1. Introduction

Multidrug-resistant (MDR) *Acinetobacter baumannii* has become a critical threat to human health globally [1]. It has a high incidence of nosocomial infections, including bacteremia, pneumonia, wound sepsis, and urinary tract infections [2]. Recently, the World Health Organization (WHO) identified carbapenem-resistant *A. baumannii* as one of three top-priority pathogens that urgently require novel antimicrobial therapeutics [3].

Antibiotics target essential components of bacterial growth, such as DNA replication, translation, and peptidoglycan biosynthesis [4]. However, resistance to antibiotics can develop rapidly in bacteria [5]. Virulence factors, such as outer membrane proteins, secretion systems, phospholipases, and iron acquisition systems, promote bacterial intracellular replication, cell adhesion, and invasion, and are crucial for pathogens to adapt to the host environment [6]. Strategies to inhibit virulence factors represent a promising alternative therapeutic option for the treatment of severe infections caused by MDR bacteria [7]. Dual RNA-seq is increasingly used to identify the key factors contributing to host adaptation during infection [8,9,10,11]. Several key metabolic pathways (e.g., the phenylacetic acid metabolism pathway) are associated with the establishment of *A. baumannii* infection in vivo [12,13]; however, it remains unclear how exactly host adaption is controlled by the complex bacterial metabolic network during infection. 

Genome-scale metabolic modeling (GSMM) is increasingly used to decipher the metabolic changes in pathogens under infection or antibiotic treatment conditions [14,15,16]. Integration with multi-omics data enabled GSMMs to accurately describe cellular metabolism [17]. In the present study, we report the development and validation of the first GSMM for *A. baumannii* MDR strain AB5075. By incorporating in vivo transcriptomics data as constraints, our model was able to identify the significant metabolic changes of AB5075 during infection in mice. This is the first integrative modeling of *A. baumannii* infection in vivo and provides key mechanistic information regarding bacterial metabolic changes in response to the host immune system. Such information will facilitate optimization of antibiotic therapy for infections caused by *A. baumannii*. 

## 2. Materials and Methods

### 2.1. Bacterial Strain and Growth Condition

Hypervirulent MDR *A. baumannii* AB5075 was obtained from the University of Washington and stored at −80 °C in tryptone soy broth (TSB, Oxoid Australia) with 20% glycerol. Prior to experiments, AB5075 was sub-cultured onto nutrient agar and incubated at 37 °C overnight. A single colony was then selected and grown overnight in 20 mL of cation-adjusted Mueller-Hinton broth (MHB; Oxoid, Australia; 20–25 mg·L^−1^ Ca^2+^ and 10–12.5 mg·L^−1^ Mg^2+^), from which a 1:100 dilution was performed in fresh broth to prepare mid-logarithmic cultures (OD_600nm_ = 0.4 to 0.6). All broth cultures were incubated at 37 °C in an open-air shaker (200 rpm). Final bacterial suspensions were concentrated to 1 × 10^10^ CFU·mL^−1^ in sterile saline. 

### 2.2. Animals

Animal experiments were approved by Monash University Animal Ethics Committee. For all animal experiments, Swiss mice (female, 8–10 weeks, body weight 25–35 g) were obtained from Monash Animal Services. Animals were handled, fed, and housed according to the criteria of the Australian Code of Practice for the Care and Use of Animals for Scientific Purposes [18]. Food and water were available *ad libitum*.

### 2.3. Non-Neutropenic Murine Bacteremia Model

A non-neutropenic murine bacteremia infection model was employed in this study. Mice were briefly anesthetized via placement into an isoflurane induction chamber. Anesthetized mice were placed on a Perspex support in a vertical upright position, which allowed the mice to be temporarily immobilized. Two independent groups of mice (*n* = 3 per group) were injected with 10 μL of bacterial suspension (approximately 1.0 × 10^9^ colony-forming units (CFUs) in early logarithmic phase) via an intravenous injection at 0 h and then placed onto a warm pad for rapid recovery. Our preliminary studies showed that bacterial infection established in mice after approximately 2 h post bacterial inoculation, and bacterial load peaked at approximately 4 h post bacterial inoculation. Therefore, we used 2 and 4 h post inoculation in this study to represent the establishment of bacterial infection and the maximum bacterial load, respectively. Samples were collected for RNA extraction and sequencing at Genewiz (paired-end 150 bp, Illumina HiSeq, Suzhou, China). Raw reads were submitted to the Sequence Read Archive Database (accession number: SRS7522398) [19].

### 2.4. Construction of the Genome-Scale Metabolic Model (GSMM) iAB5075

The genome annotation of *A. baumannii* AB5075 (i.e., AB5075-UW) was obtained from the PGAT database [20] and a draft model was initially constructed using CarveMe [21]. Further manual curation was conducted, including (i) adding transport reactions and extracellular metabolites; (ii) detecting and filling pathway gaps; and (iii) checking the mass and charge balance for each reaction. The obtained model was compiled in Systems Biology Markup Language (SBML) [22].

### 2.5. Biolog Assay and Prediction of Nutrient Utilizations

AB5075 was subcultured onto nutrient agar and incubated at 37 °C for 20 h. Biolog phenotype microarrays (PMs; Cell Biosciences, Australia) were employed to test the utilization of 190 carbon and 95 nitrogen sources, with 3 independent biological replicates. Bacterial growth was detected after 18 and 24 h of incubation at 37 °C by measuring the optical density at 595 nm using an Infinite M200 microplate reader (Tecan, Mannedorf, Switzerland). Readings with ≥1.5-fold of blank media controls were considered as utilization of nutrients. The constructed GSMM *i*AB5075 was then employed to predict the bacterial growth on a chemically defined media with 190 individual carbon sources and 95 nitrogen sources using flux balance analysis (FBA) method with COBRA toolbox 3.0 [23]. Biomass formation was optimized with the maximum specific carbon nutrient uptake rate set at 10 mmol·gDW^−1^·h^−1^ under aerobic condition [24]:

$$\max\;\;\;\;v_{biomass},$$

s.t.  S·v=0,

$$v_{j}^{\min}\leq v_{j}\leq v_{j}^{\max},j=1,2,\cdots ,n,$$

where S represents the stoichiometric matrix with *m* metabolites and *n* reactions. Each flux $v_{j}$ is constrained by the lower bound $v_{j}^{\min}$ and upper bound $v_{j}^{\max}$. The prediction accuracy was calculated by comparison with Biolog experimental results as previously described [21]. Briefly, a true positive (TP) or true negative (TN) was considered as a correct prediction of a utilizable or non-utilizable nutrient source for growth, respectively; a false negative (FN) or false positive (FP) was considered an incorrect prediction of a utilizable or non-utilizable nutrient source, respectively. The prediction accuracy was then calculated by:

$$\mathrm{overall}\;\mathrm{accuracy}=\frac{\;\left(TP+TN\right)}{\left(TP+TN+FP+FN\right)}$$

The Matthews correlation coefficient (MCC) [25] was then calculated by:

$$\frac{\;TP\times TN-PF\times PN}{\sqrt{\left(TP+FP\right)\left(TP+FN\right)\left(TN+FP\right)\left(TN+FN\right)}}$$

### 2.6. Gene Essentiality Analysis

In silico single-gene deletion was conducted using both FBA and minimization of metabolic adjustment (MOMA) algorithms [14]. FBA predicts growth and metabolic fluxes based on the assumption that growth efficiency has evolved to an optimal point using linear programming. In contrast to FBA, MOMA does not assume optimality of growth. Instead, MOMA relaxes the assumption of optimal growth flux for gene deletions by performing distance minimization in flux space using quadratic programming [14]. Nutrient uptake constraints were set according to ingredients of chemically defined M9, MH, and Luria-Bertani (LB) media. Essential metabolites were predicted by calculating the growth rate when switching off the corresponding consuming fluxes, and essential reactions were predicted by setting each reaction flux to zero while maximizing the biomass formation [15]. The recently generated three-allele transposon mutant library for *A. baumannii* AB5075 was employed as a reference to assess the prediction accuracy as previously described [26]. 

### 2.7. Calculating Metabolic Fluxes with Transcriptomics Constraints

The in vivo RNA-Seq data (accession number: SRS7522398) of AB5075 were incorporated to *i*AB5075 using INIT (Integrative Network Inference for Tissues) algorithm, which is formulated as a mixed integer linear programming problem (MILP) shown below [27]:

$$\max\underset{i\in R}{\sum }w_{i}y_{i}$$

s.t.     S·v=0,

$$\left|v_{i}\right|\leq 1000y_{i},$$

$$\left|v_{i}\right|+1000\left(1-y_{i}\right)\geq \varepsilon ,$$

$$v_{i}\geq 0,\;i\in \mathrm{irreversible}\;\mathrm{rxns},$$

$$y_{i}\in \left\{0,1\right\}.$$

The parameter *ε* is an arbitrarily small positive number, which was set by default in COBRA toolbox; *w_i_* is the weight of the *i*th reaction calculated using transcriptomic data; and *y_i_* is an integer variable indicating either including (*y_i_* = 1) or excluding (*y_i_* = 0) of the *i*th reaction in the extracted model [23]. The optimal trade-off between including and removing reactions was based on their weights (*w*) [27], which were calculated as follows:

$$w_{i,j}=5\log\left(\frac{RPKM_{i,j}}{Average_{i}}\right).$$

Specifically, RPKM (reads per kilobase per million) values were calculated using edgeR and employed to calculate the weights *w* [28]. Briefly, all the RNA-seq data of AB5075 available from the public database (Gene Expression Omnibus) and our previous studies were collected and used for estimation of the average expression level of each gene. If the RPKM of the *i*th gene in the *j*th condition is higher than its average across all the samples, *w_i,j_* is positive. Otherwise, *w_i,j_* is negative. An weights-containing objective function is then maximized to achieve the best agreement of the calculated metabolic fluxes with the transcriptomic data. Moreover, to further improve the prediction, additional protein crowding constraints were incorporated into the extracted model. The total abundance of metabolic enzyme (*P*) was set to 0.25 g⋅gDW^−1^ according to previous *Escherichia coli* data due to the limited quantitative proteomics data in *A. baumannii* [14]. The molecular weight (*MW_k_*) of *k*th protein was calculated based on its amino acid sequence:

$$\max v_{\mathrm{biomass}}=c\cdot v^{T},$$

s.t. S·v=0,

$$v_{k}^{\min}≦\;v_{k}≦v_{k}^{\max},$$

$$v_{k}≦k_{\mathrm{cat},k}\cdot e_{k},$$

$$\sum e_{k}MW_{k}=P,$$

where reaction flux $v_{k}$ is limited by the enzyme turnover rate *k*_cat,*k*_ and enzyme molar abundance $e_{k}$ as previously described [14,29]. Due to the lack of enzyme kinetic data in *A. baumannii*, an averaged *k*_cat_ (65 s^−1^) in *E. coli* was used unless specific *k*_cat_ values were available in BRENDA database (e.g., 2740 s^−1^ for xanthine dehydrogenase catalyzing reaction R_HXAND [30], 1 s^−1^ for methionyl aminopeptidase catalyzing reaction R_AMPTALAGLN [31], 11.14 s^−1^ for D-Alanine-d-alanine ligase catalyzing reaction R_ALAALAr [32], 34 s^−1^ for NAD^+^ synthase catalyzing reaction R_NADS1 [33]). FBA was conducted using COBRA toolbox 3.0 [23] in MATLAB environment and the significantly perturbed metabolic fluxes were identified using a *Z*-score based approach [34]. Briefly, genome-scale metabolic models for AB5075 at 2 and 4 h post-infection were obtained with the growth constraints (0.21 and 0.30 h^−1^ for 2 and 4 h post-infection, respectively) calculated from the bacterial viable counts. Metabolic solution space was sampled with 10000 random points with the ll-ACHRB (loopless Artificially Centered Hit-and-Run on a Box) algorithm and linear programming solver Gurobi 9.0 [35]. Statistical significance of differential flux distributions was estimated using a *Z*-score method. Differential metabolic fluxes were filtered with FDR < 0.05 and fold change > 2.

## 3. Results

### 3.1. Construction of the Genome-Scale Metabolic Model iAB5075

**Figure 1:**
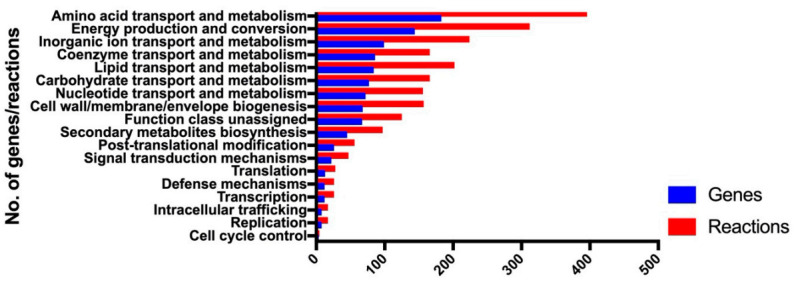
Clusters of orthologous (COG) functional classification of the genes involved in *i*AB5075.

A draft model involving 1480 metabolites and 2184 reactions was developed by CarveMe based on genome annotation. Further manual curation was conducted, including (i) addition of transport and exchange reactions to enable nutrient uptake and by-product secretion, and (ii) filling pathway gaps. Acinetobactin is the essential siderophore for iron uptake in *A. baumannii* [36]. To make our GSMM more representative of *A. baumannii*, 15 reactions and 20 metabolites involved in acinetobactin biosynthesis were added to the draft model. Altogether, a final model *i*AB5075 was obtained that involved 1530 metabolites and 2229 reactions (Table 1). *i*AB5075 includes 1015 genes representing 26.1% of the genome and 18 of 23 clusters of orthologous groups (COG, Figure 1); the majority of genes were from amino acid transport and metabolism, energy production and conversion, and pathways involving transport and metabolism of inorganic ion, coenzyme, lipid, carbohydrate and nucleotide, as well as cell envelope biogenesis (Figure 1). *i*AB5075 contained intracellular, extracellular, and periplasmic compartments (Table 1). Compared with other GSMMs (i.e., *i*ATCC19606v2 for strain ATCC 19606 and *i*CN718 for strain AYE), *i*AB5075 represents the most comprehensive GSMM for *A. baumannii* determined thus far (Table 1).

### 3.2. Prediction of Bacterial Growth on Various Nutrients

**Figure 2:**
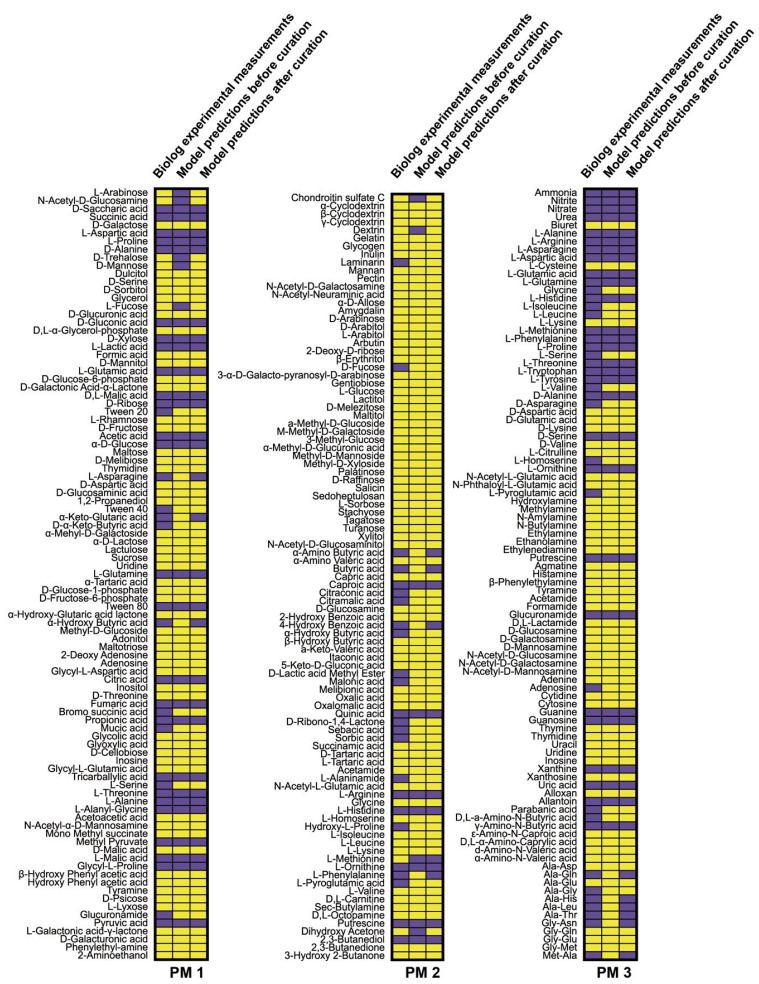
Comparison of Biolog experimental measurements (left columns) and model predictions before (middle columns) and after curation (right columns). Purple indicates valid growth (i.e., growth predicted by our model or based on the Biolog results) and yellow indicates no growth. Results from 190 carbon sources (PM1 and 2) and 95 nitrogen (PM3) sources are displayed.

With additional protein crowding constraints, model *i*AB5075 predicted bacterial exponential growth at 1.87, 2.64, 0.97, and 0.72 h^−1^ in Luria-Bertani (LB) media, Mueller-Hinton (MH) media, M9 media supplemented with citrate (M9C), and M9 media supplemented with succinate (M9S), respectively; these specific growth rates are consistent with experimental observations (Figure S1). Moreover, *i*AB5075 predicted that strain AB5075 was able to utilize 41 of 190 carbon sources and 34 of 95 nitrogen sources (Figure 2). Manual curation was conducted to enhance the prediction accuracy. A false positive was usually caused by incorrectly involving transport reactions during automatic model construction; these reactions were then removed from the draft model according to the literature. While a false negative was likely caused by mis-annotation of gene functions; extensive gap filling with homology search was conducted to add the missing reactions. For example, predictions using the draft model showed that AB5075 was unable to grow with glycyl-l-asparagine or l-alanyl-l-histidine as the sole nitrogen sources; whereas Biolog results indicated that it could. We then added the related transport reactions (R_DIPEPabc8 and R_DIPEPabc5, respectively) and two aminopeptidase reactions (R_AMPTGLYASN and R_AMPTALAHIS, ABUW_2837 and ABUW_3646, with 57% and 58% identity of their *E. coli* homologs, respectively) to enable *in silico* utilization of these two nutrients. Further prediction under anaerobic conditions was undertaken using *i*AB5075. The prediction results showed no growth under anaerobic conditions and it is consistent with the fact that *A. baumannii* is an obligate aerobe. The overall prediction accuracy of nutrient utilization achieved was high (86.3%; Fisher’s exact test, *P* = 2.2 × 10^−16^) compared with Biolog assay in which AB5075 grew on 59 carbon sources and 46 nitrogen sources (Figure 2). Core reactome analysis was conducted for all flux-carrying reactions in LB, MH, and M9 media supplemented with 73 different carbon and nitrogen sources. A core reactome containing 202 flux-carrying reactions was identified (Figure S2a), with 224 and 236 reactions as core reactomes for carbon and nitrogen sources, respectively. These core reactions are involved in 13 COG groups, including energy production and conversion, and transport and metabolism of lipid and amino acid (Figure S2b). Overall, the pan reactome consists of 1240 flux-carrying reactions, with 1139 and 1068 flux-carrying reactions specifically detected for growth on carbon and nitrogen sources, respectively.

### 3.3. Prediction of Essential Genes, Reactions, and Metabolites for Bacterial Growth

**Figure 3:**
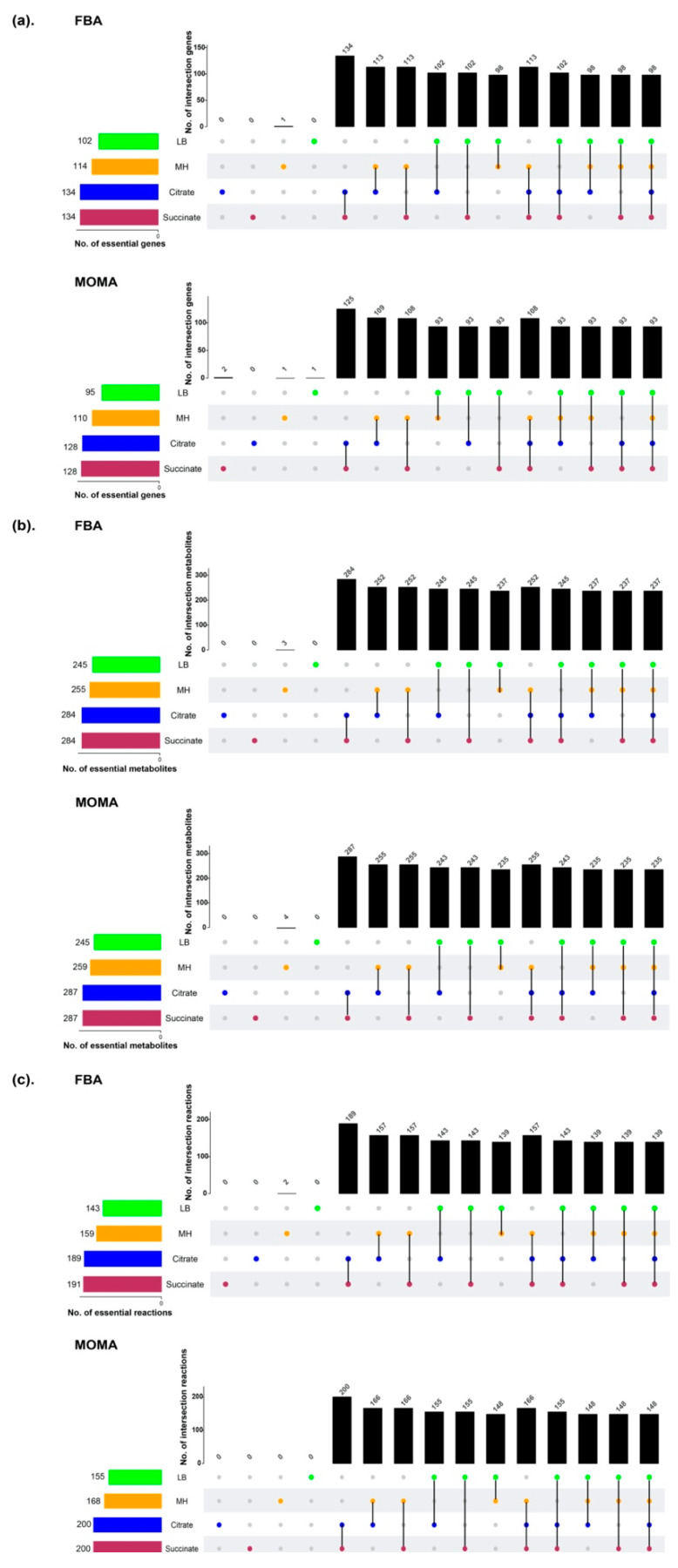
Essential genes, metabolites, and reactions predicted under four nutrient conditions using flux balance analysis (FBA) and minimization of metabolic adjustment (MOMA). The four nutrient conditions were LB media, MH media, M9 media supplemented with citrate (M9C), and M9 media supplemented with succinate (M9S). Results for FBA are shown at top and those for MOMA at bottom. (**a**) Essential genes, (**b**) essential metabolites, and (**c**) essential reactions. The circles represent different media (green, LB; orange, MH; blue, M9+citrate; red, M9+succinate). The vertical black bars represent the numbers of essential genes (**a**), metabolites (**b**), or reactions (**c**) uniquely or commonly identified for different media. The horizontal bars indicate the numbers of essential genes (**a**), metabolites (**b**), or reactions (**c**) totally identified for differential media.

In silico single gene deletion was conducted, followed by calculating the specific growth rate after iteratively removing each individual gene and its associated reactions. With the FBA approach, 102, 114, 134, and 134 genes were identified as essential for growth in LB, MH, M9C, and M9S, respectively; 95, 110, 126, and 128 genes were predicted to be essential for the respective media with the MOMA approach. Overall, 99 and 94 core essential genes were discovered using FBA and MOMA, respectively, for the 4 growth conditions (Figure 3). These genes were involved in energy production, biosynthesis of cofactors and cell envelope, and metabolism of amino acids, nucleotides, and lipids. When the prediction of gene essentiality was compared with the AB5075 three-allele transposon mutant library, *i*AB5075 showed a high accuracy of 87.6% using FBA and 88.5% with MOMA. Similarly, 155/143, 168/159, 200/189, and 200/191 reactions and 245/251, 255/259, 284/287, and 284/287 metabolites were considered essential for bacterial growth on LB, MH, M9C, and M9S media using FBA and MOMA, respectively (Figure 3 and Supplementary Materials Dataset 1). Comparison of FBA and MOMA core essential components revealed 89 core essential genes, 137 core essential reactions, and 237 core essential metabolites, indicating a substantially high agreement between both methods. Taken together, the prediction accuracies using draft *i*AB5075 obtained directly from CarveMe were only 72.8% and 74.3% for Biolog nutrient utilization and gene essentiality, respectively; whereas after curation, the corresponding prediction accuracies were significantly improved and achieved 86.3% and 87.6%. Overall, *i*AB5075 exhibits precise prediction of gene essentiality and thus can be utilized to systematically interrogate genotype–phenotype relationships.

### 3.4. Modeling Metabolic Changes of Strain AB5075 in a Murine Bacteremia Model with Transcriptomic Constraints

*A. baumannii* may alter its metabolism to adapt to the host environment during infection [37]. However, our understanding of such complex metabolic responses is very limited. With a combination of fold change > 2 and false discovery rate (FDR)-adjusted *p* value < 0.05, a total of 1408 genes in *A. baumannii* AB5075 were differentially expressed at 4 h post-infection compared to 2 h, and 396 genes were mapped to *i*AB5075 (Table S1). Based on pathway enrichment analysis (Fisher’s exact test, FDR-adjusted *p*-value < 0.05), the differentially expressed genes were mainly enriched in the following pathways: intracellular trafficking, secretion and vesicular transport; secondary metabolites biosynthesis, transport, and catabolism; inorganic ion transport and metabolism; and translation, ribosomal structure, and biogenesis. Among those genes mapped to *i*AB5075, 283 were identified as being involved with transport and metabolism of carbohydrates, nucleotides, and amino acids; energy production and conversion; and cell envelope metabolism, indicating their critical roles in adaption to the host environment.

The transcriptomic data were then incorporated in the model as flux constraints to accurately predict metabolic fluxes using the INIT algorithm. In addition, constraints on bacterial growth rate and protein crowding were also imposed on the model. Specifically, 478 flux-carrying reactions were detected at 2 h post infection, while 502 reactions had non-zero fluxes at 4 h post infection. Compared to 2 h post infection, 198 significantly changed fluxes (fold change > 2, FDR < 0.05) were identified at 4 h post infection, including 146 increased and 52 decreased fluxes (Table S2). The increased fluxes were mainly detected in a broad range of metabolic pathways, including the tricarboxylic acid (TCA) cycle, gluconeogenesis, amino acid metabolism, and biosynthesis of peptidoglycan, lipopolysaccharide (LPS), nucleotides, and fatty acids. 

**Figure 4:**
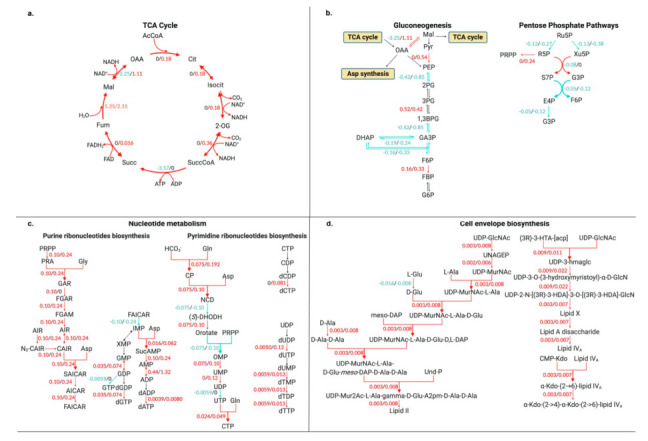
Differentially regulated metabolic fluxes in multiple pathways at 4 h post infection compared to 2 h post infection. Differentially regulated metabolic fluxes through (**a**) the TCA cycle, (**b**) the gluconeogenesis and pentose phosphate pathways, (**c**) nucleotide metabolism, and (**d**) cell structure biosynthesis. Significantly regulated fluxes (fold change > 2 and FDR < 0.05) are shown in color with red (produced fluxes) or blue (consumed fluxes). AcCoA, acetyl-CoA; CIT, citrate; Isocit, isocitrate; 2-OG, α-ketoglutarate; SuccCoA, succinyl-CoA; Succ, succinate; Fum, fumarate; MAL, (*S*)-malate; OAA, oxaloacetate; Pyr, pyruvate; PEP, phosphoenolpyruvate; 2PG, 2-phosphoglycerate; 3PG, 3-phosphoglycerate; 1,3-BPG, 1,3-bisphosphoglycerate; GA3P, glyceraldehyde 3-phosphate; DHAP, dihydroxyacetone phosphate; F6P, fructose 6-phosphate; FBP, fructose 1,6-biphosphate; G6P, glucose 6-phosphate; R5P, ribose 5-phosphate; Ru5P, ribulose 5-phosphate; Xu5P, xylulose 5-phosphate; S7P, sedoheptulose 7-phosphate; PRPP, phosphoribosyl pyrophosphate; PRA, 5-phospho-β-d-ribosylamine; Gly, glycine; GAR, *N*^1^-(5-phospho-β-d-ribosyl)glycinamide; FGAR, *N*^2^-formyl-*N*^1^-(5-phospho-β-d-ribosyl)glycinamide; FGAM, 2-(formamido)-*N*^1^-(5-phospho-β-d-ribosyl)acetamidine; AIR, 5-amino-1-(5-phospho-β-d-ribosyl)imidazole; CAIR, 5-amino-1-(5-phospho-d-ribosyl)imidazole-4-carboxylate; SAICAR, 5’-phosphoribosyl-4-(*N*-succinocarboxamide)-5-aminoimidazole; AICAR, 5-amino-1-(5-phospho-d-ribosyl)imidazole-4-carboxamide; FAICAR, 5-formamido-1-(5-phospho-d-ribosyl)-imidazole-4-carboxamide; Asp, l-aspartate; SucAMP, adenylo-succinate; Gln, L-glutamine; CP, carbamoyl phosphate; NCD, N-carbamoyl-l-aspartate; (S)-DHODH, (S)-dihydroorotate; UDP-GlcNAc, UDP-*N*-acetyl-α-d-glucosamine; UNAGEP, UDP-*N*-acetyl-α-d-glucosamine-enolpyruvate; UDP-MurNAc, UDP-*N*-acetyl-α-d-muramate; UDP-MurNAc-l-Ala, UDP-*N*-acetyl-α-d-muramoyl-l-alanine; UDP-MurNAc-l-Ala-d-Glu, UDP-*N*-acetyl-α-d-muramoyl-l-alanyl-d-glutamate; *meso*-DAP, *meso*-diaminopimelate; UDP-MurNAc-l-Ala-d-Glu-d,l-DAP, UDP-*N*-acetyl-α-d-muramoyl-l-alanyl-γ-d-glutamyl-*meso*-2,6-diaminopimelate; UDP-MurNAc-l-Ala-d-Glu-*meso*-DAP-d-Ala-d-Ala, UDP-*N*-acetyl-α-d-muramoyl-l-alanyl-γ-d-glutamyl-*meso*-2,6-diaminopimeloyl-d-alanyl-d-alanine; Und-P, *di-trans,octa-cis*-undecaprenyl phosphate; (3R)-3-HTA-[acp], (3*R*)-3-hydroxytetradecanoyl-[acp]; UDP-3-hmaglc, UDP-3-*O*-[(3*R*)-3-hydroxydecanoyl]-*N*-acetyl-α-d-glucosamine; UDP-3-*O*-(3-hydroxymyristoyl)-α-d-GlcN, UDP-3-O-(3-hydroxymyristoyl)-*N*-acetyl-α-d-glucosamine; UDP-2-*N*-[(3*R*)-3-HDA]-3-*O*-[(3*R*)-3-HDA]-GlcN, UDP-2-*N*-[(3*R*)-3-hydroxydodecanoyl]-3-*O*-[(3*R*)-3-hydroxydecanoyl]-α-d-glucosamine.

In the TCA cycle, six of eight metabolic fluxes were significantly increased at 4 h post-infection compared to 2 h post-infection, while the flux through fumarate hydratase was significantly decreased (Figure 4a). Notably, the production flux through malate dehydrogenase was 1.11 mmol·gDW·h^−1^ from malate to oxaloacetate 4 h post infection, whereas the flux from oxaloacetate to malate was 3.25 mmol·gDW·h^−1^; the two metabolites (malate and oxaloacetate) were utilized by the gluconeogenesis pathway, resulting in significantly increased fluxes through gluconeogenesis (Figure 4b). At the same time, most fluxes through the pentose phosphate pathway (PPP) were significantly increased, whereas the flux from d-ribose 5-phospahte towards d-sedoheptulose 7-phosphate was significantly decreased (Figure 4b). Remarkably, the flux thorough 5-phospho-α-d-ribose 1-diphosphate (PRPP) synthase was dramatically increased (0 mmol·gDW·h^−1^ at 2 h post infection versus 0.24 mmol·gDW·h^−1^ at 4 h post infection), and the increased PRPP was then utilized in the biosynthesis of purine and pyrimidine nucleotides.

The increased fluxes in the PPP toward nucleotide metabolism were significantly increased 4 h post infection (Figure 4b). As the downstream utilization of PRPP, the overall fluxes via enzymes (e.g., amido phosphoribosyl transferase, phosphoribosyl amine-glycine ligase, and phosphoribosyl glycinamide formyltransferase) in inosine-5’-phosphate biosynthesis were increased, resulting in the increased production of dGTP and dATP from guanosine and adenosine ribonucleotide *de novo* biosynthesis (Figure 4c). Additionally, increased fluxes involving pyrimidine deoxyribonucleotide biosynthesis were also identified, which enhanced the production of dCTP and dTTP (Figure 4c).

At 4 h post-infection, there were remarkable increases in fluxes involving the biosynthesis of cell envelope components (e.g., peptidoglycan and LPS biosynthesis). At this time, most fluxes within peptidoglycan biosynthesis from UDP-*N*-acetyl-α-d-glucosamine to *meso*-diaminopimelate containing lipid II were increased 266.6% to 300.0%, whereas the flux over glutamate racemase was significantly decreased (Figure 4d). In lipid A biosynthesis, fluxes through acyl-ACP-UDP-*N*-acetylglucosamine *O*-acyltransferase, UDP-3-*O*-acyl-*N*-acetylglucosamine deacetylase, UDP-3-*O*-(3-hydroxymyristoyl) glucosamine *N*-acyltransferase, UDP-2,3-diacylglucosamine diphosphatase, lipid-A-disaccharide synthase, and tetraacyldisaccharide 4’-kinase were upregulated, resulting in a dramatic increase in the production of lipid A (Figure 4d). 

In addition to the increased fluxes through the above metabolic pathways, fluxes through fatty acid biosynthesis and amino acid metabolism were also significantly impacted at both 2 and 4 h post infection. In fatty acid biosynthesis, most fluxes were significantly increased at 4 h compared to 2 h (Table S3a), whereas 71 key fluxes in amino acid metabolism were significantly affected at each time point (Table S3b).

## 4. Discussion

*A. baumannii* is a Gram-negative opportunistic pathogen which has imposed a heavy burden on the global health care system [38]. There is an urgent need to understand how *A. baumannii* responds to the host immune system during infection. GSMM has been increasingly employed to predict key genetic targets in order to guide therapeutic interventions and decipher the mechanism(s) of antibiotic killing and resistance. Unfortunately, few studies have previously examined bacterial metabolic responses in vivo using GSMMs, and none of these studies examined *A. baumannii*. We report here, for the first time, the development, validation, and application of a GSMM named *i*AB5075 for the MDR clinical isolate *A. baumannii* AB5075.

To the best of our knowledge, the only GSMMs previously developed for *A. baumannii* are for strains ATCC 19606 and AYE [14,16,39,40]. However, ATCC 19606 and AYE were isolated in the 1950s and 2003, respectively, and have significant differences in their genomic content and virulence phenotypes compared to more recent clinical isolates [41]. In addition, of both isolates, only AYE is MDR. Importantly, AB5075, the focus of the present study was a well-characterized modern clinical isolate that has been employed as a type strain of *A. baumannii* to investigate virulence and multidrug resistance [41,42,43]. Given this situation, we developed and validated an AB5075-specific GSMM, *i*AB5075 (Figure 2 and Figure 3). Compared to the most recent GSMM in AYE (*i*CN718), *i*AB5075 contains a larger number of unique genes, metabolites, and reactions, and has a much higher prediction accuracy (i.e., 87.6% for *i*AB5075 compared to 80.2% for *i*CN718). These differences are mainly attributable to the different compartment settings, transport and exchange reactions contained within the two models (Table 1), and indicate that *i*AB5075 represents the most comprehensive GSMM thus far developed for *A. baumannii*.

Transcriptomics is increasingly employed to examine the mechanisms underpinning bacterial pathogenesis and antibacterial resistance in Gram-negative pathogens in vitro and in vivo [13,44,45,46]. However, no study to date has integrated in vivo transcriptomic data with GSMM in *A. baumannii*. It is important to note that the nutrient complex composition varies from in vivo to in vitro based on nutrient conditions [47]. In the present study, the integration and simulation with in vivo transcriptomic data from the murine bloodstream infection model provide an important expansion of genes involved with adaption to the host environment. This, in turn, enabled a global view of metabolic responses to the host at the network level. Compared to 2 h post infection, 4 h post infection induced significant metabolic changes in biosynthesis of nucleotides, peptidoglycan, lipopolysaccharide, and fatty acids; and central carbon and amino acid metabolism.

When INIT algorithm was applied to the RNA-seq data, 198 fluxes were identified as critical for AB5075 to cause serious bloodstream infections in mice at 4 h post infection (Figure 4). These fluxes represent a novel set of metabolic functions, which are integral to the establishment of *A. baumannii* infections. Within central metabolism, the upregulated fluxes were associated with TCA cycle, gluconeogenesis, and pentose phosphate pathway (Figure 4a,b). The increased production flux in TCA cycle via malate dehydrogenase at 4 h resulted in enhanced fluxes in gluconeogenesis (Figure 4b). The downstream metabolites from glucogenesis were subsequently utilized by PPP pathway, with most of the identified PPP fluxes being dramatically increased (Figure 4b). It has previously been shown that the TCA cycle and gluconeogenesis play key roles in the virulence of *Salmonella enterica* during infection [48]. A number of intermediates in the TCA cycle and gluconeogenesis have been identified to contribute to the virulence in macrophages in vivo [48]. Our observations suggest that the increased fluxes through central metabolic pathways might have important consequences for bloodstream infections, given that *A. baumannii* relies on these pathways to provide adequate energy metabolism and to contribute to the establishment and enhancement of infection.

*A. baumannii* can evade the host innate immune response using multiple virulence factors, such as surface glycoconjugates [49]. An important barrier that protects *A. baumannii* against host immune responses during infection is the outer membrane. In *A. baumannii*, the primary component of the outer leaflet of outer membrane is LPS, which consists of lipid A, a core oligosaccharide, and a polysaccharide *O*-antigen. Model simulation showed that fluxes through lipid A biosynthesis were significantly increased (up to 267%) 4 h post infection compared to 2 h post infection. In addition to lipid A biosynthesis, increased fluxes were also detected in peptidoglycan biosynthesis via the conversion of UDP-*N*-acetyl-α-d-glucosamine to *meso* diaminopimelate containing lipid II. It has previously been shown that genes involved in the biosynthesis and maintenance of LPS and peptidoglycan contribute to the fitness of *A. baumannii* during bacteremia [9], and that both LPS and peptidoglycan contribute to bacterial cell stability and resistance to lysozyme in the host environment [50,51]. During infection, the significantly increased fluxes in LPS and peptidoglycan biosynthesis are in line with these previous findings, suggesting that both metabolic pathways contribute to bacterial fitness during infection in the murine host. The balance of LPS and fatty acid biosynthesis plays an important role in maintaining cell envelope function and integrity in *A. baumannii* [52]. Compared to 2 h post infection, most fluxes through fatty acids biosynthesis were significantly increased at 4 h post infection, consistent with the increased fluxes in LPS biosynthesis. These increased fluxes are crucial for rebalancing fatty acids and LPS to maintain membrane integrity.

In addition to surface glycoconjugates, other strategies also contribute to bacterial defense against host immune systems, including secreted proteins and multiple other regulators and metabolic pathways (e.g., OmpA porin, BfmR global regulator, phenylacetic acid catabolism pathways). Nucleotide second messengers (e.g., cAMP, cyclic di-GMP, and *penta*/*tetra*-guanosine phosphate) are key bacterial regulators involved in adaptations to conditions of limiting or non-optimal carbon and energy resources [53,54]. Cyclic di-GMP and other nucleotide second messengers control a variety of processes (e.g., production of exopolysaccharides, protein adhesins, pili and flagella, and cell differentiation) and have a central role in modulating virulence and persistence [55]. Increased fluxes were detected in inosine-5’-phosphate biosynthesis I, inosine-5’-phosphate biosynthesis II, and pyrimidine ribonucleotide biosynthesis (Figure 4c), indicating that AB5075 increased production of nucleotide to adapt to the host environment. As the downstream production in nucleotide metabolism, second messengers most likely contributed to the regulation of surface adaption and virulence factors that assisted with initiation of infections in the mice. Finally, fluxes via amino acid metabolism were significantly impacted in AB5075 during bacteremia. These effects might be attributed to the adaption of metabolic changes in multiple pathways.

Overall, fluxes were significantly changed through multiple pathways in AB5075 at 4 h post infection compared with 2 h post infection. Marked fluxes through central metabolism, nucleotide metabolism, and fatty acid and cell envelope biosynthesis might contribute to adaptations to the host environment and enhanced infection during bacteremia. These impacted pathways may be potential therapeutic targets for future drug development and therapy optimization.

## 5. Conclusions

In summary, we constructed and validated the first GSMM, *i*AB5075, for a modern model strain of MDR *A. baumannii*, namely AB5075. This is the first study to integrate in vivo transcriptomic data with GSMM, and importantly, the modeling provides a precise understanding of metabolic changes in *A. baumannii* in response to a host immune system during bacteremia. Model *i*AB5075 provides a unique >in silico platform for predicting bacterial metabolic responses to different treatments at the network level, which may assist in the optimization of antibiotic treatment in patients.

## Figures and Tables

**Table 1:** Genome contents and model components.

Content	*i*AB5075_draft_	*i*AB5075	*i*ATCC19606v2	*i*CN718
Genome size (Mb)	3.97		3.98	3.90
Assembly status	Complete		Complete	Complete
GC content	39.1%		39.2%	39.0%
No. of genes	3895		3805	3694
No. of CDS	3771		3663	3600
No. of reactions	2184	2229	2114	1016
No. of metabolites	1480	1530	1422	890
No. of involved genes	1010	1015	1009	718
Compartment ^a^	3 (c, p, e)	3 (c, p, e)	3 (c, p, e)	2(c, e)
Prediction accuracy ^b^	72.8%/74.3%	86.3%/87.6%	85.6%/82.1%	83.7/80%
MCC ^b^	0.58/0.26	0.76/0.28	0.68/0.33	0.72/0.32

Comparison of genome and GSMM features among isolates AB5075 (*i*AB5075), ATCC19606 (*i*ATCC19606v2) and AYE (*i*CN718) [14,15,16]; ^a^ the compartment in the models; c: intracellular compartment; p: periplasmic compartment; e: extracellular compartment; ^b^ Prediction accuracy and Mathews correlation coefficients for Biolog and gene essentiality results.

## Supplementary Materials

The following are available online at https://www.mdpi.com/2076-2607/8/11/1793/s1.
