# Supplementary material for: Genome-Scale Metabolic Modeling Reveals Metabolic Alterations of Multidrug-Resistant Acinetobacter baumannii in a Murine Bloodstream Infection Model
Source: Microorganisms. 2020 Nov 16;8(11):1793. doi: 10.3390/microorganisms8111793 (PMC7696501; doi:10.3390/microorganisms8111793)
Supplement: Supplementary file 1 [file microorganisms-08-01793-s001.zip › Supplementary materials/Supplementary materials 20201112.docx]

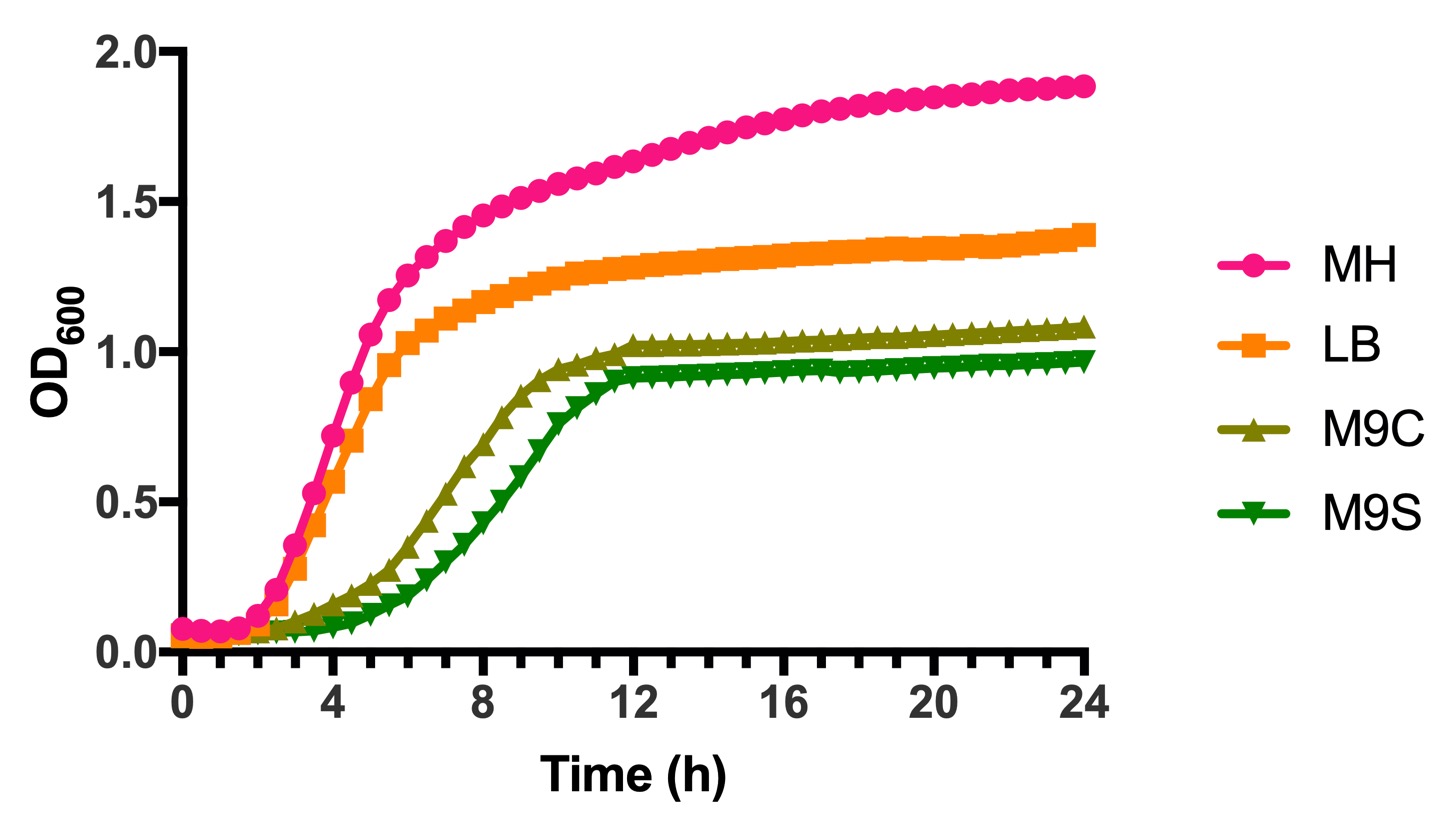
**Figure S1.**

**Figure S1.** **Growth curves for *A. baumannii* AB5075 in LB, MH, M9C and M9S**.

**
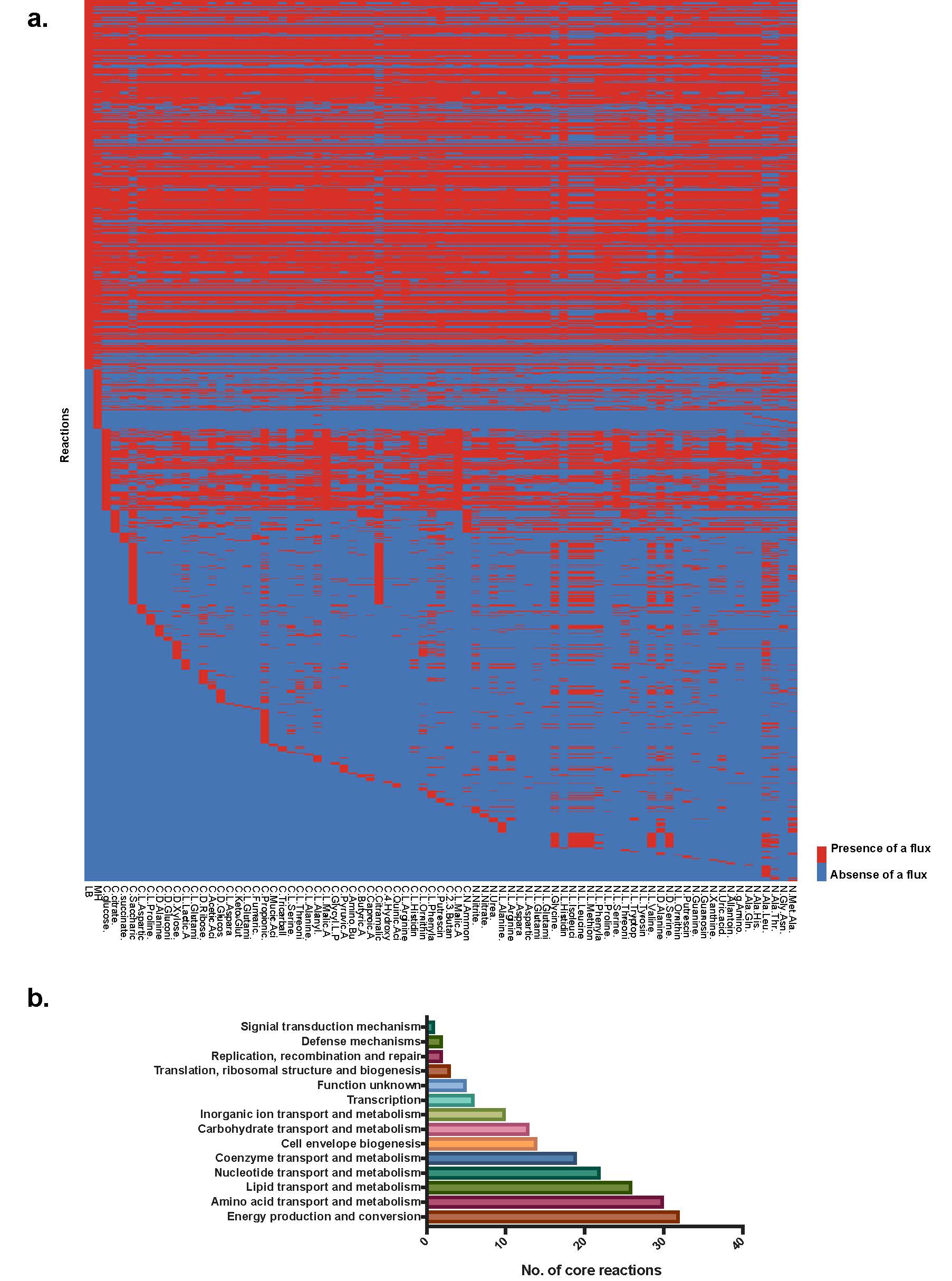
Figure S2.**

**Figure S2. (a)** Flux-carrying reactions under 75 nutrient conditions. **(b)** COG functional classification of the core reactome.

**Table** **S1.** The differentially expressed genes during infection.

| **Gene ID** | **Fold change** | **FDR** | **Gene name** | **Annotation** |
| --- | --- | --- | --- | --- |
| ABUW_2515 | 6.77586202 | 9.05E-16 | *-* | hypothetical protein |
| ABUW_2510 | 6.50661304 | 3.99E-13 | *-* | hypothetical protein |
| ABUW_1621 | 6.45225534 | 9.01E-19 | *ald1* | aldehyde dehydrogenase |
| ABUW_1703 | 6.35202713 | 0.00409498 | *-* | hypothetical protein |
| ABUW_1427 | 5.79209974 | 0.0004626 | *-* | hypothetical protein |
| ABUW_2608 | 5.66820209 | 0.00142243 | *-* | hypothetical protein |
| ABUW_1624 | 5.49840349 | 1.27E-17 | *-* | alcohol dehydrogenase, iron-containing |
| ABUW_2540 | 5.29647651 | 0.00464492 | *-* | transposase |
| ABUW_2891 | 5.13782194 | 0.00892678 | *-* | hypothetical protein |
| ABUW_2691 | 4.95029329 | 2.94E-17 | *-* | transporter LysE family |
| ABUW_4081 | 4.93375809 | 3.8434E-06 | *-* | hypothetical protein |
| ABUW_2514 | 4.78186881 | 3.95E-09 | *-* | hypothetical protein |
| ABUW_2696 | 4.68549536 | 7.66E-21 | *-* | hypothetical protein |
| ABUW_3783 | 4.68307759 | 3.22E-19 | *mmsA1* | methylmalonate-semialdehyde dehydrogenase |
| ABUW_1700 | 4.58042377 | 0.02839044 |  |  |
| ABUW_2756 | 4.54278006 | 1.71E-12 | *-* | hypothetical protein |
| ABUW_2507 | 4.50888901 | 9.03E-29 | *-* | L-carnitine dehydratase/bile acid-inducible protein F |
| ABUW_0010 | 4.39623489 | 5.40E-16 | *adeT* | RND type efflux pump |
| ABUW_2513 | 4.38412403 | 1.15E-42 | *csp2* | cold-shock DNA-binding domain protein |
| ABUW_1473 | 4.31427177 | 3.08E-24 | *-* | hypothetical protein |
| ABUW_0727 | 4.31390051 | 7.34E-29 | *-* | fatty acid desaturase |
| ABUW_3782 | 4.29392033 | 1.68E-18 | *mmsB* | 3-hydroxyisobutyrate dehydrogenase |
| ABUW_2509 | 4.28101195 | 5.76E-12 |  |  |
| ABUW_1785 | 4.26783781 | 3.76E-16 | *-* | hypothetical protein |
| ABUW_1472 | 4.23620923 | 5.52E-16 | *-* | hypothetical protein |
| ABUW_1820 | 4.10367235 | 4.25E-21 | *-* | NADH:flavin oxidoreductase/nadh oxidase |
| ABUW_3508 | 4.09890188 | 1.64E-20 | *-* | hypothetical protein |
| ABUW_3125 | 4.07309136 | 1.05E-26 | *bfr2* | bacterioferritin |
| ABUW_2703 | 3.97243497 | 6.40E-15 | *-* | hypothetical protein |
| ABUW_2512 | 3.96205696 | 1.15E-10 | *-* | hypothetical protein |
| ABUW_1239 | 3.9515806 | 8.57E-20 | *-* | branched-chain amino acid permease |
| ABUW_2511 | 3.91496935 | 1.01E-16 | *-* | hypothetical protein |
| ABUW_1819 | 3.84728784 | 1.95E-29 | *-* | FAD/FMN-binding/pyridine nucleotide- disulfide oxidoreductase family protein |
| ABUW_1640 | 3.83076595 | 0.00013543 | *-* | hypothetical protein |
| ABUW_1962 | 3.79520732 | 3.0743E-06 |  |  |
| ABUW_2687 | 3.77969467 | 3.22E-12 | *-* | hypothetical protein |
| ABUW_2700 | 3.77718025 | 5.38E-20 | *-* | hypothetical protein |
| ABUW_2505 | 3.74835812 | 3.16E-14 | *-* | major facilitator superfamily MFS_1 |
| ABUW_2325 | 3.70444017 | 2.69E-15 | *-* | transcriptional regulator, TetR family |
| ABUW_4080 | 3.69827728 | 9.81E-08 | *-* | hypothetical protein |
| ABUW_3781 | 3.63177167 | 1.63E-14 | *-* | acetyl-coenzyme A synthetase |
| ABUW_1112 | 3.59611087 | 1.67E-07 | *-* | transcriptional regulator, AsnC family |
| ABUW_2658 | 3.59321926 | 2.39E-14 | *-* | hypothetical protein |
| ABUW_0934 | 3.58934971 | 2.10E-07 | *-* | hypothetical protein |
| ABUW_2458 | 3.57500913 | 4.57E-08 | *-* | oxidoreductase |
| ABUW_1821 | 3.57150117 | 5.28E-26 | *-* | hypothetical protein |
| ABUW_2516 | 3.56550721 | 1.54E-08 | *-* | hypothetical protein |
| ABUW_2438 | 3.55851704 | 0.0005065 | *cinA1* | competence/damage-inducible protein CinA |
| ABUW_2451 | 3.52507768 | 4.93E-12 | *-* | transcriptional regulator AcrR family |
| ABUW_2811 | 3.51693685 | 9.60E-22 | *-* | hypothetical protein |
| ABUW_1787 | 3.4972673 | 1.78E-13 | *-* | hypothetical protein |
| ABUW_1662 | 3.4897619 | 0.02158484 | *-* | hypothetical protein |
| ABUW_2534 | 3.47233155 | 4.0101E-05 | *paaH* | phenylacetate-CoA oxygenase, PaaH subunit |
| ABUW_2689 | 3.42280066 | 4.02E-16 | *-* | hypothetical protein |
| ABUW_4123 | 3.41090858 | 1.18E-12 | *-* | hypothetical protein |
| ABUW_0831 | 3.38879935 | 1.6274E-05 | *-* | ammonium transporter |
| ABUW_3216 | 3.3497283 | 3.33E-13 | *-* | zinc-binding dehydrogenase |
| ABUW_1113 | 3.32225042 | 1.07E-07 | *-* | indole-3-pyruvate decarboxylase |
| ABUW_2448 | 3.3142502 | 4.67E-17 | *-* | hypothetical protein |
| ABUW_2535 | 3.31192042 | 1.05E-07 | *paaG* | phenylacetate-CoA oxygenase, PaaG subunit |
| ABUW_1519 | 3.3116777 | 6.66E-11 | *-* | aspartate racemase |
| ABUW_2428 | 3.31065771 | 4.2356E-06 | *-* | sensor histidine kinase/response regulator |
| ABUW_2630 | 3.30689654 | 5.11E-26 | *-* | lipid A biosynthesis lauroyl acyltransferase |
| ABUW_1950 | 3.27269194 | 2.25E-07 | *-* | multidrug resistance protein VceB |
| ABUW_2450 | 3.2676788 | 8.98E-12 | *-* | long-chain fatty-acid-CoA ligase |
| ABUW_1240 | 3.26764735 | 1.9083E-06 | *-* | hypothetical protein |
| ABUW_1943 | 3.23589364 | 1.88E-24 | *-* | nicotinamide phosphoribosyltransferase |
| ABUW_2690 | 3.23493057 | 1.08E-20 | *csp1* | cold-shock DNA-binding domain protein |
| ABUW_2701 | 3.22349509 | 2.83E-21 | *-* | amino acid efflux pump, RhtB family |
| ABUW_2533 | 3.21337967 | 2.44E-07 | *paaI2* | phenylacetate-CoA oxygenase, PaaI subunit |
| ABUW_2130 | 3.19076033 | 1.42E-09 | *acoB* | acetoin:2,6-dichlorophenolindophenol oxidoreductase subunit beta |
| ABUW_4007 | 3.18118522 | 5.18E-26 | *-* | hypothetical protein |
| ABUW_4009 | 3.18063921 | 1.3644E-06 | *-* | hypothetical protein |
| ABUW_3702 | 3.16546314 | 1.14E-12 | *-* | hypothetical protein |
| ABUW_2506 | 3.15154824 | 6.54E-12 | *-* | 3-hydroxy-3-methylglutaryl-CoA lyase |
| ABUW_2064 | 3.14901741 | 0.00188557 | *-* | hypothetical protein |
| ABUW_2452 | 3.13066305 | 1.73E-08 | *-* | isovaleryl-CoA dehydrogenase |
| ABUW_4091 | 3.11836958 | 4.66E-12 | *-* | hypothetical protein |
| ABUW_0935 | 3.09299646 | 6.08E-08 | *-* | hypothetical protein |
| ABUW_2219 | 3.07084633 | 8.06E-08 | *-* | hypothetical protein |
| ABUW_1620 | 3.06514829 | 2.14E-07 | *eat* | ethanolamine permease |
| ABUW_1210 | 3.06478027 | 4.82E-20 | *-* | hypothetical protein |
| ABUW_1286 | 3.06330708 | 1.3779E-06 | *-* | hypothetical protein |
| ABUW_1494 | 3.06306959 | 5.34E-14 | *-* | hypothetical protein |
| ABUW_2822 | 3.04827476 | 2.12E-15 | *-* | 2,4-dienoyl-CoA reductase |
| ABUW_2834 | 3.0401145 | 2.13E-10 | *-* | hypothetical protein |
| ABUW_1918 | 3.01931464 | 2.3712E-05 | *-* | hypothetical protein |
| ABUW_1708 | 3.01128346 | 2.93E-14 | *-* | molybdopterin oxidoreductase |
| ABUW_2614 | 3.00384578 | 3.59E-09 | *-* | hypothetical protein |
| ABUW_2318 | 2.99685478 | 1.17E-08 | *-* | cytosine/purines/uracil/thiamine/allantoin permease family protein |
| ABUW_2132 | 2.98453066 | 3.52E-14 | *lipA2* | lipoic acid synthetase |
| ABUW_2430 | 2.98066055 | 6.6007E-06 | *-* | transporter, LysE family |
| ABUW_2812 | 2.97468064 | 2.73E-17 | *-* | hypothetical protein |
| ABUW_3245 | 2.97400485 | 3.62E-11 | *-* | FilA |
| ABUW_3213 | 2.9688903 | 4.04E-13 | *mucK2* | cis,cis-muconate transport protein |
| ABUW_2145 | 2.95570308 | 2.71E-09 | *-* | hypothetical protein |
| ABUW_2056 | 2.95145211 | 1.01E-25 | *-* | hypothetical protein |
| ABUW_1340 | 2.94517429 | 0.0002383 | *hisQ* | histidine transport system permease protein HisQ |
| ABUW_1306 | 2.94386905 | 0.00625315 | *-* | hypothetical protein |
| ABUW_0304 | 2.93327444 | 1.35E-18 | *-* | type IV pilin structural subunit |
| ABUW_2922 | 2.93057721 | 1.44E-10 | *-* | hypothetical protein |
| ABUW_0183 | 2.92376032 | 4.25E-17 | *-* | hypothetical protein |
| ABUW_3388 | 2.90199013 | 7.25E-15 | *-* | putative hydrolase |
| ABUW_4008 | 2.88526589 | 6.57E-19 | *-* | hypothetical protein |
| ABUW_1040 | 2.88418057 | 2.34E-12 | *-* | hypothetical protein |
| ABUW_2532 | 2.87951917 | 1.528E-06 | *paaJ* | phenylacetate-CoA oxygenase, PaaJ subunit |
| ABUW_3353 | 2.87614177 | 1.0626E-06 | *-* | putative transcriptional regulator |
| ABUW_2814 | 2.8546051 | 0.00021731 | *-* | hypothetical protein |
| ABUW_2144 | 2.84233797 | 5.67E-15 | *-* | hypothetical protein |
| ABUW_2953 | 2.84171729 | 0.00062084 | *-* | rieske (2Fe-2S) protein |
| ABUW_2041 | 2.83320224 | 1.6099E-06 | *-* | hypothetical protein |
| ABUW_3804 | 2.81683119 | 8.9644E-06 | *-* | hypothetical protein |
| ABUW_2324 | 2.80945221 | 2.92E-08 | *-* | heme oxygenase-like protein |
| ABUW_0226 | 2.8067923 | 3.30E-07 | *-* | hypothetical protein |
| ABUW_3191 | 2.78710045 | 1.95E-14 | *-* | hypothetical protein |
| ABUW_1339 | 2.78641377 | 7.8506E-05 | *hisJ* | histidine-binding periplasmic protein |
| ABUW_0184 | 2.78225832 | 3.35E-15 | *-* | Na+/solute symporter |
| ABUW_2772 | 2.77135287 | 9.95E-12 | *vanK* | vanillate transporter |
| ABUW_2688 | 2.76799817 | 1.30E-18 | *-* | pyrroline-5-carboxylate reductase |
| ABUW_3004 | 2.76391878 | 8.8495E-05 | *-* | hypothetical protein |
| ABUW_2770 | 2.75986358 | 2.82E-18 | *-* | GMC oxidoreductase |
| ABUW_2539 | 2.75113208 | 0.04240247 | *-* | transposase inhibitor |
| ABUW_2771 | 2.75084363 | 1.40E-11 | *-* | porin |
| ABUW_4121 | 2.7490016 | 1.29E-15 | *-* | hypothetical protein |
| ABUW_2061 | 2.74633741 | 1.06E-13 | *-* | hypothetical protein |
| ABUW_2796 | 2.74333216 | 1.13E-11 | *-* | hypothetical protein |
| ABUW_0936 | 2.7393358 | 0.00011376 | *-* | hypothetical protein |
| ABUW_3037 | 2.73823452 | 8.81E-18 | *-* | hemerythrin/HHE cation-binding motif- containing protein |
| ABUW_1903 | 2.73519974 | 3.28E-11 | *-* | hypothetical protein |
| ABUW_2813 | 2.73141346 | 1.57E-07 | *-* | hypothetical protein |
| ABUW_0933 | 2.72944904 | 1.94E-11 | *-* | hypothetical protein |
| ABUW_1635 | 2.72609033 | 5.63E-12 | *-* | glutathione S-transferase |
| ABUW_0673 | 2.7153823 | 1.69E-11 | *-* | hypothetical protein |
| ABUW_1942 | 2.70346224 | 1.19E-09 | *-* | ribose-phosphate pyrophosphokinase |
| ABUW_4002 | 2.68822274 | 0.00012712 | *-* | hypothetical protein |
| ABUW_0433 | 2.68075162 | 3.18E-12 | *-* | flavin-containing monooxygenase FMO |
| ABUW_2131 | 2.67472637 | 2.20E-08 | *acoA* | acetoin:2,6-dichlorophenolindophenol oxidoreductase alpha subunit |
| ABUW_4084 | 2.67307208 | 9.0618E-06 | *-* | hypothetical protein |
| ABUW_2454 | 2.67256361 | 6.3978E-06 | *mgh* | 3-methylglutaconyl-CoA hydratase |
| ABUW_1066 | 2.66900065 | 2.44E-09 | *-* | hypothetical protein |
| ABUW_2875 | 2.66229141 | 3.52E-13 | *-* | hypothetical protein |
| ABUW_0809 | 2.64152349 | 4.9308E-06 | *-* | hypothetical protein |
| ABUW_3809 | 2.63682264 | 2.71E-10 | *-* | transcriptional regulator, GntR family |
| ABUW_3192 | 2.62699557 | 1.86E-11 | *-* | hypothetical protein |
| ABUW_2541 | 2.6112386 | 1.73E-08 | *-* | amino acid transporter |
| ABUW_2449 | 2.60436821 | 4.2734E-06 | *-* | putative SAM-dependent methyltransferase |
| ABUW_0448 | 2.60333966 | 3.18E-11 | *-* | HAD-superfamily subfamily IB hydrolase |
| ABUW_1288 | 2.60114299 | 3.04E-13 | *-* | hypothetical protein |
| ABUW_2091 | 2.59685642 | 1.00E-11 | *-* | transcriptional regulator, AraC family |
| ABUW_2900 | 2.58880644 | 1.90E-13 | *-* | hypothetical protein |
| ABUW_2543 | 2.5834236 | 6.85E-10 | *-* | shikimate transporter |
| ABUW_1786 | 2.58336268 | 4.39E-13 | *-* | hypothetical protein |
| ABUW_1753 | 2.58017948 | 4.13E-11 | *-* | hypothetical protein |
| ABUW_1674 | 2.57342272 | 1.62E-11 | *-* | acetyl-CoA acyltransferase |
| ABUW_2623 | 2.56837159 | 1.30E-08 | *-* | 3-hydroxyacyl-CoA dehydrogenase |
| ABUW_1597 | 2.55311895 | 6.69E-11 | *-* | hypothetical protein |
| ABUW_3121 | 2.54737782 | 3.25E-14 | *-* | biotin biosynthesis protein BioH |
| ABUW_1792 | 2.53984282 | 3.91E-11 | *-* | hypothetical protein |
| ABUW_2127 | 2.53927212 | 6.13E-08 | *budC* | acetoin dehydrogenase |
| ABUW_2143 | 2.53489821 | 1.73E-08 | *-* | hypothetical protein |
| ABUW_1290 | 2.5297814 | 1.40E-07 | *-* | hypothetical protein |
| ABUW_3214 | 2.52878089 | 6.33E-10 | *gcdH* | glutaryl-CoA dehydrogenase |
| ABUW_1752 | 2.51862734 | 1.89E-10 | *-* | hypothetical protein |
| ABUW_4010 | 2.51811687 | 1.0161E-05 | *-* | hypothetical protein |
| ABUW_1629 | 2.51336572 | 3.29E-07 | *-* | hypothetical protein |
| ABUW_0120 | 2.50772371 | 1.69E-13 | *-* | hypothetical protein |
| ABUW_2427 | 2.50489896 | 0.00025281 | *-* | putative two-component sensor kinase |
| ABUW_2530 | 2.50153459 | 7.8043E-05 | *caiD* | enoyl-coA hydratase |
| ABUW_1463 | 2.49916968 | 7.78E-08 | *-* | omega-amino acid--pyruvate aminotransferase |
| ABUW_1465 | 2.49882696 | 4.26E-09 | *-* | gamma-aminobutyrate permease |
| ABUW_2453 | 2.49776547 | 1.7153E-06 | *-* | methylcrotonoyl-CoA carboxylase beta chain |
| ABUW_2043 | 2.4819272 | 0.0267551 | *-* | hypothetical protein |
| ABUW_2319 | 2.47889903 | 5.8951E-06 | *-* | putative membrane protein |
| ABUW_2678 | 2.4716058 | 5.46E-12 | *-* | 17 kDa surface antigen |
| ABUW_2615 | 2.45733097 | 2.2373E-06 | *-* | hypothetical protein |
| ABUW_3122 | 2.45671148 | 0.00010437 | *otsB* | trehalose-phosphatase |
| ABUW_0672 | 2.45410188 | 0.02125244 | *-* | transposase inhibitor |
| ABUW_1775 | 2.45331991 | 4.43E-09 | *-* | hypothetical protein |
| ABUW_1192 | 2.45013922 | 1.34E-12 | *-* | hypothetical protein |
| ABUW_1462 | 2.44263905 | 1.62E-09 | *-* | transcriptional regulator, LysR family |
| ABUW_0567 | 2.4422722 | 2.05E-09 | *-* | phage tail sheath protein |
| ABUW_2686 | 2.43957171 | 1.14E-12 | *-* | hypothetical protein |
| ABUW_1516 | 2.43879586 | 0.01751859 | *-* | hypothetical protein |
| ABUW_1474 | 2.42774683 | 0.00218944 | *-* | threonine efflux protein |
| ABUW_0306 | 2.425034 | 2.71E-15 | *bfr1* | bacterioferritin |
| ABUW_1751 | 2.42488505 | 4.10E-09 | *-* | putative prophage protein |
| ABUW_0895 | 2.42061003 | 6.65E-10 | *-* | hypothetical protein |
| ABUW_3351 | 2.42018517 | 3.40E-11 | *-* | heme oxygenase-like protein |
| ABUW_1540 | 2.41859364 | 5.00E-11 | *-* | hypothetical protein |
| ABUW_1982 | 2.41538356 | 1.90E-11 | *-* | outer membrane protein E |
| ABUW_4015 | 2.40911225 | 2.38E-11 | *-* | hypothetical protein |
| ABUW_1794 | 2.40839757 | 3.98E-11 | *cydB* | cytochrome D ubiquinol oxidase, subunit II |
| ABUW_3194 | 2.40339284 | 0.00365277 | *-* | transcriptional regulator, TetR family |
| ABUW_3244 | 2.40181523 | 3.51E-08 |  |  |
| ABUW_3067 | 2.40016069 | 1.26E-10 | *-* | pirin domain protein |
| ABUW_1238 | 2.39727484 | 1.68E-09 | *-* | transcriptional regulator, AraC family |
| ABUW_0071 | 2.393815 | 2.30E-08 | *aroP1* | aromatic amino acid transport protein |
| ABUW_1692 | 2.39371973 | 1.65E-08 | *-* | transcriptional regulator, TetR family |
| ABUW_1561 | 2.39338362 | 1.73E-08 | *-* | hypothetical protein |
| ABUW_1241 | 2.39269198 | 9.30E-11 | *-* | hypothetical protein |
| ABUW_1486 | 2.39069781 | 5.21E-09 | *-* | transcriptional regulator, TetR family protein |
| ABUW_2611 | 2.38492311 | 1.01E-14 | *-* | hypothetical protein |
| ABUW_1636 | 2.38394647 | 8.28E-10 | *-* | short chain dehydrogenase |
| ABUW_2536 | 2.38351567 | 1.47E-08 | *paaN* | phenylacetic acid degradation protein PaaN |
| ABUW_3860 | 2.37781337 | 0.00026279 | *-* | C4-dicarboxylate transporter/malic acid transport protein |
| ABUW_3683 | 2.35619145 | 0.00014951 | *-* | hypothetical protein |
| ABUW_1661 | 2.35415305 | 2.87E-11 | *-* | universal stress protein family |
| ABUW_1913 | 2.34441077 | 2.212E-06 | *-* | hypothetical protein |
| ABUW_1601 | 2.33345291 | 5.26E-10 | *-* | hypothetical protein |
| ABUW_1675 | 2.33252981 | 7.23E-11 | *-* | short-chain dehydrogenase/reductase |
| ABUW_0070 | 2.33135195 | 8.72E-09 | *fahA* | fumarylacetoacetase |
| ABUW_1795 | 2.32355294 | 9.33E-12 | *cydA* | cytochrome D ubiquinol oxidase subunit I |
| ABUW_2333 | 2.31788084 | 1.47E-07 | *gltI* | glutamate/aspartate ABC transporter, periplasmic glutamate/aspartate-binding protein |
| ABUW_4122 | 2.31543704 | 1.25E-14 | *-* | hypothetical protein |
| ABUW_4082 | 2.31445912 | 1.82E-07 | *-* | hypothetical protein |
| ABUW_2702 | 2.30657113 | 3.4049E-06 | *-* | hypothetical protein |
| ABUW_3567 | 2.30498513 | 1.27E-10 | *-* | acyl-CoA thioesterase II |
| ABUW_3571 | 2.30172683 | 2.70E-10 | *-* | lipoprotein, putative |
| ABUW_2551 | 2.29795446 | 1.73E-08 | *-* | dihydrodipicolinate synthetase |
| ABUW_0077 | 2.29785538 | 1.6106E-05 | *hutU* | urocanate hydratase |
| ABUW_2106 | 2.29660994 | 4.68E-07 | *pcaT* | metabolite:H+ symporter |
| ABUW_3079 | 2.29443261 | 2.72E-07 | *-* | hypothetical protein |
| ABUW_3880 | 2.28928348 | 3.52E-08 | *-* | matrixin superfamily |
| ABUW_3012 | 2.27770852 | 0.00100639 | *-* | hypothetical protein |
| ABUW_1484 | 2.27092045 | 2.13E-10 | *-* | transcriptional regulator, GntR family |
| ABUW_1914 | 2.26792708 | 8.32E-09 | *-* | hypothetical protein |
| ABUW_2613 | 2.26198313 | 2.85E-11 | *-* | RHS family protein |
| ABUW_3143 | 2.26039673 | 3.16E-14 | *-* | amino acid efflux pump, RhtB family |
| ABUW_0439 | 2.25205573 | 4.67E-08 | *-* | rhomboid family peptidase |
| ABUW_1677 | 2.24802659 | 1.69E-13 | *-* | beta-lactamase |
| ABUW_3520 | 2.23984183 | 2.00E-10 | *-* | beta-lactamase |
| ABUW_2586 | 2.23481 | 0.00029494 | *atzF* | allophanate hydrolase |
| ABUW_2297 | 2.23400258 | 7.94E-08 | *-* | hypothetical protein |
| ABUW_0646 | 2.23289293 | 5.72E-08 | *-* | hypothetical protein |
| ABUW_2667 | 2.22807267 | 0.00239073 | *-* | putative bacteriophage protein |
| ABUW_3387 | 2.22802799 | 4.06E-10 | *-* | hypothetical protein |
| ABUW_4016 | 2.22548888 | 1.51E-07 | *-* | hypothetical protein |
| ABUW_0374 | 2.22447517 | 1.35E-09 | *-* | acyl-CoA dehydrogenase |
| ABUW_2531 | 2.22349065 | 0.0001792 | *paaK* | phenylacetate-CoA oxygenase/reductase, PaaK subunit |
| ABUW_1901 | 2.22146857 | 8.3749E-05 | *-* | hypothetical protein |
| ABUW_2833 | 2.2165016 | 3.12E-09 | *-* | hypothetical protein |
| ABUW_0225 | 2.20915866 | 1.99E-09 | *-* | hypothetical protein |
| ABUW_1467 | 2.2079918 | 1.0301E-06 | *-* | acyl-CoA dehydrogenase |
| ABUW_2784 | 2.20716671 | 0.01959814 | *-* | hypothetical protein |
| ABUW_2387 | 2.20361825 | 2.233E-05 | *-* | hypothetical protein |
| ABUW_4119 | 2.20349574 | 0.00956553 |  |  |
| ABUW_2455 | 2.1989403 | 1.99E-07 | *-* | methylcrotonoyl-CoA carboxylase subunit alpha |
| ABUW_1693 | 2.19818162 | 3.637E-05 | *-* | heme oxygenase-like protein |
| ABUW_0119 | 2.19563532 | 3.52E-08 | *-* | hypothetical protein |
| ABUW_2621 | 2.18100814 | 7.60E-09 | *-* | hypothetical protein |
| ABUW_4113 | 2.17613504 | 2.3258E-05 | *-* | hypothetical protein |
| ABUW_1705 | 2.17608855 | 0.00023837 | *-* | FAD-dependent pyridine nucleotide- disulfide oxidoreductase |
| ABUW_3018 | 2.172755 | 3.30E-07 | *ggt* | gamma-glutamyltransferase |
| ABUW_2487 | 2.17088804 | 0.00055138 | *pobA* | 4-hydroxybenzoate 3-monooxygenase |
| ABUW_0440 | 2.16863756 | 1.80E-09 | *-* | TRAP C4-dicarboxylate transport system permease |
| ABUW_1754 | 2.16665587 | 4.90E-11 | *-* | acetyltransferase gnat family |
| ABUW_3490 | 2.16439454 | 7.61E-10 | *rpmE2* | ribosomal protein L31 |
| ABUW_0130 | 2.15099122 | 0.00134963 | *uraH1* | transthyretin |
| ABUW_4014 | 2.14115756 | 1.17E-07 | *-* | hypothetical protein |
| ABUW_3757 | 2.13146039 | 8.38E-08 | *-* | DoxX family protein |
| ABUW_0921 | 2.12948046 | 1.93E-08 | *-* | glycerophosphoryl diester phosphodiesterase |
| ABUW_2129 | 2.12867313 | 2.65E-08 | *acoC* | dihydrolipoamide acetyltransferase |
| ABUW_3307 | 2.12420428 | 1.42E-10 | *-* | enoyl-CoA hydratase |
| ABUW_2720 | 2.11103821 | 8.6794E-05 | *-* | hypothetical protein |
| ABUW_0991 | 2.11028983 | 4.29E-07 | *-* | hypothetical protein |
| ABUW_2332 | 2.10887064 | 6.4122E-06 | *gltJ* | glutamate/aspartate transport system permease protein GltJ |
| ABUW_0068 | 2.10724341 | 3.64E-07 | *-* | glyoxalase/bleomycin resistance protein/dioxygenase |
| ABUW_2704 | 2.10699075 | 0.00589647 | *-* | hypothetical protein |
| ABUW_3459 | 2.10620469 | 1.50E-07 | *-* | hypothetical protein |
| ABUW_2425 | 2.10579848 | 2.73E-07 | *-* | acetyltransferase, gnat family |
| ABUW_0683 | 2.10461633 | 1.57E-09 | *-* | hypothetical protein |
| ABUW_2593 | 2.10286635 | 0.01270974 | *-* | urea carboxylase-associated protein 2 |
| ABUW_2329 | 2.09998204 | 0.00010208 | *-* | hypothetical protein |
| ABUW_2437 | 2.09599559 | 6.25E-12 | *-* | heme oxygenase-like protein |
| ABUW_4011 | 2.09456491 | 2.83E-07 | *-* | hypothetical protein |
| ABUW_2581 | 2.09413194 | 6.80E-10 | *-* | hypothetical protein |
| ABUW_2331 | 2.09397365 | 3.2034E-06 | *gltK* | glutamate/aspartate transport system permease protein GltK |
| ABUW_2388 | 2.09371658 | 0.00134781 | *-* | hypothetical protein |
| ABUW_2795 | 2.09209551 | 2.0048E-05 | *-* | hypothetical protein |
| ABUW_0069 | 2.08421227 | 1.89E-07 | *maiA* | maleylacetoacetate isomerase |
| ABUW_2628 | 2.0827039 | 2.849E-05 | *pcaK* | 4-hydroxybenzoate transporter |
| ABUW_0887 | 2.08231713 | 6.21E-15 | *-* | hypothetical protein |
| ABUW_2901 | 2.08227354 | 1.7153E-06 | *-* | activator of HSP90 ATPase |
| ABUW_0788 | 2.07948073 | 0.00135926 | *-* | hypothetical protein |
| ABUW_1902 | 2.07925813 | 6.95E-10 | *sndH2* | L-sorbosone dehydrogenase |
| ABUW_1773 | 2.07918691 | 2.32E-07 | *-* | alpha/beta hydrolase |
| ABUW_2773 | 2.07833137 | 1.30E-08 | *vanA* | vanillate O-demethylase oxygenase subunit |
| ABUW_2193 | 2.07490194 | 8.25E-09 | *-* | acyl-CoA dehydrogenase |
| ABUW_2128 | 2.07074112 | 2.00E-07 | *lpdA2* | dihydrolipoamide dehydrogenase |
| ABUW_2676 | 2.05942984 | 5.186E-05 | *-* | hypothetical protein |
| ABUW_0479 | 2.05652189 | 0.00025372 | *-* | hypothetical protein |
| ABUW_1493 | 2.05562378 | 2.4078E-06 | *-* | hypothetical protein |
| ABUW_1145 | 2.05421287 | 4.34E-08 | *-* | glycine cleavage system transcriptional activator |
| ABUW_1632 | 2.04680431 | 5.4857E-06 | *-* | hypothetical protein |
| ABUW_3462 | 2.04036552 | 1.77E-14 | *leuD* | 3-isopropylmalate dehydratase, small subunit |
| ABUW_3252 | 2.04011193 | 1.3757E-06 | *raiA* | ribosomal subunit interface protein |
| ABUW_2831 | 2.03738843 | 1.41E-13 | *-* | hypothetical protein |
| ABUW_0585 | 2.03704494 | 1.12E-09 | *-* | two-component system histidine kinase sensor component |
| ABUW_1899 | 2.03333851 | 6.0957E-05 | *-* | hypothetical protein |
| ABUW_3766 | 2.03306723 | 7.0135E-05 | *-* | phosphopantetheine-protein transferase |
| ABUW_2671 | 2.03282206 | 0.00023276 |  |  |
| ABUW_0888 | 2.0283612 | 7.36E-07 | *-* | metalloprotease |
| ABUW_1377 | 2.02796647 | 7.91E-14 | *-* | cold-shock domain protein |
| ABUW_2556 | 2.02667921 | 0.00255357 | *-* | hypothetical protein |
| ABUW_2500 | 2.00755166 | 9.1417E-05 | *-* | hypothetical protein |
| ABUW_1495 | 2.00472295 | 0.00014449 | *-* | hypothetical protein |
| ABUW_2456 | 2.00221735 | 6.3978E-06 | *-* | hydroxymethylglutaryl-CoA lyase |
| ABUW_2672 | 2.00141457 | 0.01172432 | *-* | hypothetical protein |
| ABUW_0937 | 2.00073932 | 2.4397E-05 | *-* | hypothetical protein |
| ABUW_1347 | 1.99662575 | 0.00021559 | *-* | phenazine biosynthesis protein PhzF family |
| ABUW_4068 | 1.98993719 | 0.00049225 | *-* | hypothetical protein |
| ABUW_2990 | 1.98810818 | 7.6237E-06 | *-* | major facilitator superfamily MFS_1 |
| ABUW_0645 | 1.98409288 | 0.00032771 | *-* | quaternary ammonium compound-resistance protein |
| ABUW_4001 | 1.98388246 | 3.14E-08 | *repAci6* | Replicase |
| ABUW_0298 | 1.98315075 | 4.7269E-06 | *gltB* | glutamate synthase, large subunit |
| ABUW_0889 | 1.97939923 | 8.82E-07 | *-* | methylated-DNA--protein-cysteine methyltransferase |
| ABUW_3615 | 1.97585634 | 1.07E-08 | *algR* | alginate biosynthesis regulatory protein |
| ABUW_1966 | 1.97313593 | 4.22E-08 | *-* | transcriptional regulator, LysR family |
| ABUW_0928 | 1.9708991 | 5.06E-08 | *-* | lytic transglycosylase, catalytic |
| ABUW_3026 | 1.96988372 | 1.80E-08 | *-* | glyoxalase |
| ABUW_3137 | 1.9655952 | 2.31E-08 | *-* | hypothetical protein |
| ABUW_1898 | 1.96091055 | 0.01339013 | *-* | putative acetoacetate decarboxylase |
| ABUW_1866 | 1.96032789 | 1.342E-05 | *feaR* | transcriptional regulator, AraC family |
| ABUW_0886 | 1.95850943 | 2.77E-10 | *-* | hypothetical protein |
| ABUW_1763 | 1.95838197 | 1.09E-07 | *-* | UspA domain protein |
| ABUW_1341 | 1.95611436 | 0.02379446 | *hisM* | histidine transport system permease protein HisM |
| ABUW_0129 | 1.95609973 | 0.00047718 | *-* | uracil-xanthine permease |
| ABUW_0066 | 1.95127288 | 4.7642E-06 | *hppD* | 4-hydroxyphenylpyruvate dioxygenase |
| ABUW_0566 | 1.95089623 | 6.09E-11 | *-* | phage tail tube protein |
| ABUW_0438 | 1.94994649 | 4.8454E-05 | *-* | hypothetical protein |
| ABUW_1938 | 1.94954976 | 1.5318E-05 | *-* | transporter, anion:cation symporter family |
| ABUW_0800 | 1.94839166 | 3.2669E-05 | *-* | hypothetical protein |
| ABUW_0606 | 1.94473636 | 1.41E-08 | *acdB1* | acyl-CoA dehydrogenase |
| ABUW_3759 | 1.94400146 | 2.11E-09 | *-* | hypothetical protein |
| ABUW_2151 | 1.94184061 | 1.57E-07 | *-* | poly(R)-hydroxyalkanoic acid synthase |
| ABUW_2521 | 1.93970512 | 4.90E-11 | *-* | UvrD/REP helicase |
| ABUW_1295 | 1.93649095 | 8.55E-07 | *-* | hypothetical protein |
| ABUW_2229 | 1.93237191 | 6.5559E-05 | *-* | TonB family protein |
| ABUW_0299 | 1.93217922 | 1.634E-05 | *gltD* | glutamate synthase, small subunit |
| ABUW_0274 | 1.93211927 | 6.64E-08 | *-* | hypothetical protein |
| ABUW_0577 | 1.92848648 | 0.02013804 | *-* | phage-related membrane protein |
| ABUW_4108 | 1.9247375 | 0.00069291 | *traW* | conjugative transfer protein |
| ABUW_4071 | 1.91886477 | 0.00027454 | *-* | hypothetical protein |
| ABUW_1619 | 1.91748058 | 0.00090718 | *eutB* | ethanolamine ammonia-lyase, large subunit |
| ABUW_1004 | 1.91265591 | 2.0162E-05 | *-* | hypothetical protein |
| ABUW_2906 | 1.91162973 | 0.00020933 | *-* | hypothetical protein |
| ABUW_2829 | 1.91054576 | 7.4396E-05 | *-* | hypothetical protein |
| ABUW_0394 | 1.91052218 | 5.61E-08 | *-* | putative RND type efflux pump involved in aminoglycoside resistance |
| ABUW_3291 | 1.90201814 | 3.20E-08 | *-* | acyl-CoA synthase |
| ABUW_2107 | 1.90037848 | 0.01503778 | *-* | hypothetical protein |
| ABUW_0984 | 1.89657944 | 3.37E-09 | *-* | hypothetical protein |
| ABUW_3021 | 1.89067336 | 1.17E-10 | *-* | hypothetical protein |
| ABUW_2596 | 1.88627261 | 0.01162179 | *-* | transcriptional regulator, TetR family |
| ABUW_2156 | 1.88087405 | 7.28E-07 | *-* | hypothetical protein |
| ABUW_0357 | 1.87840086 | 5.70E-07 | *-* | hypothetical protein |
| ABUW_1996 | 1.87501182 | 8.90E-11 | *-* | hypothetical protein |
| ABUW_0726 | 1.86925303 | 5.38E-09 | *-* | putative transcriptional regulator (AraC family) |
| ABUW_2499 | 1.86894302 | 2.4522E-05 | *-* | hydrolase |
| ABUW_0078 | 1.86680919 | 9.2805E-05 | *hutH* | histidine ammonia-lyase |
| ABUW_2810 | 1.86429371 | 3.40E-11 | *-* | hypothetical protein |
| ABUW_1768 | 1.85789401 | 0.00010517 | *-* | transcriptional regulator, MarR-family |
| ABUW_2580 | 1.85279938 | 3.84E-08 | *-* | type VI secretion protein, family |
| ABUW_1111 | 1.85058182 | 2.2436E-05 | *-* | aldehyde dehydrogenase |
| ABUW_3140 | 1.84988331 | 8.32E-08 | *-* | hypothetical protein |
| ABUW_3682 | 1.84772209 | 4.77E-08 | *-* | putative dihydrodipicolinate synthase |
| ABUW_2040 | 1.84616534 | 0.00285168 | *-* | hypothetical protein |
| ABUW_1129 | 1.845684 | 1.71E-07 | *-* | hypothetical protein |
| ABUW_0680 | 1.84506283 | 5.68E-09 | *pilI* | type IV pilus signal transduction protein PilI |
| ABUW_0565 | 1.84194529 | 2.74E-10 | *-* | phage tail protein |
| ABUW_3383 | 1.8416356 | 3.47E-07 | *-* | putative phosphatidylglycerophosphatase B |
| ABUW_3190 | 1.84107424 | 9.40E-08 | *-* | hypothetical protein |
| ABUW_1776 | 1.84082175 | 1.12E-09 | *-* | glycolate/propanediol utilization protein |
| ABUW_2819 | 1.83556084 | 0.00050184 | *-* | hypothetical protein |
| ABUW_2832 | 1.83551708 | 1.0884E-05 | *-* | hypothetical protein |
| ABUW_1882 | 1.83376738 | 0.00098924 |  |  |
| ABUW_1679 | 1.83291511 | 1.32E-07 | *smvA* | methyl viologen resistance protein |
| ABUW_1342 | 1.83170708 | 0.0025534 |  |  |
| ABUW_1438 | 1.82773912 | 0.00661464 | *-* | acyltransferase 3 domain-containing protein |
| ABUW_1709 | 1.82297106 | 3.0661E-06 | *moaA* | molybdenum cofactor biosynthesis protein A |
| ABUW_1633 | 1.82160201 | 5.83E-07 | *-* | hypothetical protein |
| ABUW_4035 | 1.81787964 | 9.7194E-06 | *-* | hypothetical protein |
| ABUW_0890 | 1.81760205 | 5.02E-09 | *-* | universal stress protein |
| ABUW_1823 | 1.81557695 | 1.0854E-06 | *-* | hypothetical protein |
| ABUW_4012 | 1.81351787 | 2.36E-07 | *-* | Thermonuclease protein |
| ABUW_0127 | 1.81327056 | 0.00146921 | *-* | monooxygenase, FAD-binding |
| ABUW_1879 | 1.81209411 | 1.32E-08 | *-* | hypothetical protein |
| ABUW_1622 | 1.8092891 | 1.54E-07 | *-* | putative transcriptional regulator |
| ABUW_2160 | 1.80812733 | 3.64E-08 | *-* | putative porin |
| ABUW_1543 | 1.80660181 | 4.44E-07 | *-* | hypothetical protein |
| ABUW_2213 | 1.8050497 | 2.8296E-06 | *-* | glutaredoxin |
| ABUW_4027 | 1.80376221 | 0.0004902 | *-* | hypothetical protein |
| ABUW_1948 | 1.7990577 | 7.0406E-05 | *-* | indoleacetamide hydrolase |
| ABUW_2880 | 1.79680217 | 0.00714415 | *ureF* | urease accessory protein F |
| ABUW_1928 | 1.79672426 | 0.02542755 | *-* | hypothetical protein |
| ABUW_3463 | 1.79444253 | 2.64E-12 | *leuC* | 3-isopropylmalate dehydratase, large subunit |
| ABUW_1704 | 1.79158656 | 0.00957068 | *nirB* | nitrite reductase [NAD(P)H], large subunit |
| ABUW_1062 | 1.79150912 | 1.065E-06 | *-* | hypothetical protein |
| ABUW_0605 | 1.78974156 | 4.36E-08 | *acdA* | acyl-CoA dehydrogenase |
| ABUW_0796 | 1.78675607 | 4.5239E-05 | *-* | hypothetical protein |
| ABUW_0300 | 1.78448871 | 9.54E-07 | *-* | hypothetical protein |
| ABUW_1332 | 1.78380537 | 4.9816E-05 | *-* | alkaline lipase |
| ABUW_0085 | 1.78232639 | 0.01476784 | *-* | hypothetical protein |
| ABUW_1076 | 1.77870746 | 4.961E-05 | *-* | hypothetical protein |
| ABUW_2629 | 1.77578745 | 0.01253011 | *-* | TetR family transcriptional regulator |
| ABUW_1995 | 1.77425892 | 7.01E-11 | *-* | hypothetical protein |
| ABUW_0604 | 1.77378408 | 2.0626E-05 | *-* | phosphate-starvation-inducible E |
| ABUW_2330 | 1.7736983 | 8.5737E-06 | *gltL* | glutamate/aspartate transport ATP-binding protein GltL |
| ABUW_2237 | 1.77291697 | 1.58E-08 | *-* | monooxygenase, flavin-binding family |
| ABUW_3395 | 1.76896643 | 3.95E-09 | *ackA* | acetate kinase |
| ABUW_2885 | 1.7661459 | 0.00315033 | *ureD* | urease accessory protein UreD |
| ABUW_3068 | 1.76556653 | 3.37E-07 | *-* | hypothetical protein |
| ABUW_1818 | 1.76351269 | 6.90E-07 | *-* | transcriptional regulator, PadR family |
| ABUW_2441 | 1.76221644 | 0.0012331 | *-* | hypothetical protein |
| ABUW_1114 | 1.76144121 | 0.00011912 | *aroP3* | aromatic amino acid transport protein |
| ABUW_0771 | 1.76079453 | 0.0001104 | *-* | hypothetical protein |
| ABUW_1701 | 1.76058196 | 6.3046E-05 | *-* | putative nitrate transport protein |
| ABUW_1958 | 1.75492661 | 3.39E-10 | *-* | type III restriction enzyme, res subunit |
| ABUW_0640 | 1.74990346 | 1.0691E-06 | *-* | alpha-methylacyl-CoA racemase |
| ABUW_1437 | 1.74417452 | 1.5788E-06 | *-* | hypothetical protein |
| ABUW_1367 | 1.74367464 | 7.49E-09 | *-* | hypothetical protein |
| ABUW_1385 | 1.73764099 | 0.0097764 | *-* | transposase, Mutator family |
| ABUW_0082 | 1.73370517 | 9.48E-08 | *-* | hypothetical protein |
| ABUW_1904 | 1.73104268 | 0.01416243 | *-* | antibiotic biosynthesis monooxygenase |
| ABUW_3301 | 1.72719467 | 8.4341E-06 | *-* | short chain dehydrogenase |
| ABUW_0377 | 1.72406855 | 4.95E-08 | *hemF* | coproporphyrinogen III oxidase, aerobic |
| ABUW_2341 | 1.723789 | 0.0043066 | *-* | D-methionine transport system permease protein MetI |
| ABUW_1470 | 1.72291448 | 0.00151101 | *-* | glycosyl transferase, family 2 |
| ABUW_1822 | 1.72255852 | 1.18E-10 | *-* | alpha/beta hydrolase |
| ABUW_1783 | 1.72126973 | 2.1011E-05 | *-* | CBS domain containing protein |
| ABUW_2051 | 1.71198766 | 0.00507795 | *-* | hypothetical protein |
| ABUW_3226 | 1.70972269 | 1.80E-08 | *-* | Co/Zn/Cd efflux system component |
| ABUW_4030 | 1.70618543 | 0.02884925 | *-* | hypothetical protein |
| ABUW_0368 | 1.7047838 | 7.21E-08 | *-* | tetracycline resistance protein TetA |
| ABUW_5009 | 1.70413875 | 7.36E-09 |  |  |
| ABUW_1886 | 1.70318974 | 7.58E-08 | *cpo* | alpha/beta hydrolase |
| ABUW_2152 | 1.70204793 | 1.6427E-05 | *-* | UbiE/COQ5 methyltransferase |
| ABUW_1956 | 1.69920269 | 0.00071444 | *-* | hypothetical protein |
| ABUW_4003 | 1.6968297 | 3.1325E-06 | *-* | Zeta toxin family protein |
| ABUW_1963 | 1.69186004 | 3.7026E-06 | *-* | hypothetical protein |
| ABUW_1042 | 1.68879301 | 2.3615E-05 | *-* | hypothetical protein |
| ABUW_1900 | 1.68486936 | 2.67E-08 | *-* | oxidoreductase, FAD/FMN-binding |
| ABUW_1440 | 1.68442962 | 7.4588E-05 | *-* | hypothetical protein |
| ABUW_0381 | 1.67953519 | 0.00053861 | *-* | DEAD/DEAH box helicase |
| ABUW_1710 | 1.67815466 | 0.00132107 | *-* | thiamine S/molybdopterin converting factor subunit 1 |
| ABUW_1284 | 1.67769093 | 2.09E-08 | *-* | hypothetical protein |
| ABUW_2735 | 1.67675907 | 7.3184E-06 | *-* | hypothetical protein |
| ABUW_3139 | 1.67299968 | 1.0761E-06 | *-* | hypothetical protein |
| ABUW_0985 | 1.6696675 | 2.07E-09 | *-* | hypothetical protein |
| ABUW_3217 | 1.6655226 | 7.2267E-05 | *add2* | adenosine deaminase |
| ABUW_1485 | 1.66336237 | 7.6767E-05 | *-* | hypothetical protein |
| ABUW_1915 | 1.66309324 | 3.2324E-06 | *-* | hypothetical protein |
| ABUW_2133 | 1.66075146 | 5.8502E-06 | *-* | hypothetical protein |
| ABUW_3352 | 1.65745064 | 0.0001203 | *-* | putative transcriptional regulator |
| ABUW_0328 | 1.65583362 | 1.03E-07 | *-* | hemerythrin |
| ABUW_1816 | 1.6557806 | 0.00031299 | *aro1* | 3-deoxy-7-phosphoheptulonate synthase |
| ABUW_3562 | 1.65137836 | 1.1841E-05 | *-* | putative fusaric acid resistance protein |
| ABUW_1676 | 1.64839889 | 7.27E-09 | *-* | MaoC domain protein dehydratase |
| ABUW_1747 | 1.64728036 | 2.2238E-06 | *recX* | regulatory protein RecX |
| ABUW_2607 | 1.64665462 | 4.8225E-06 | *-* | hypothetical protein |
| ABUW_1678 | 1.64462114 | 8.4566E-06 | *-* | transcriptional regulator, TetR family |
| ABUW_2194 | 1.64137673 | 6.0877E-06 | *-* | acyl-CoA dehydrogenase, middle domain protein |
| ABUW_2493 | 1.6391099 | 0.01564259 | *-* | hypothetical protein |
| ABUW_3507 | 1.63872166 | 2.5531E-06 | *csaA* | secretion chaperone |
| ABUW_1211 | 1.63738483 | 0.00082509 | *-* | hypothetical protein |
| ABUW_1075 | 1.63359978 | 0.00069726 |  |  |
| ABUW_0104 | 1.63245087 | 2.8528E-06 | *-* | hypothetical protein |
| ABUW_3141 | 1.63230667 | 1.46E-07 | *-* | hypothetical protein |
| ABUW_3193 | 1.63165544 | 1.3297E-06 | *-* | hypothetical protein |
| ABUW_4036 | 1.62867612 | 1.3962E-05 | *-* | hypothetical protein |
| ABUW_4099 | 1.62760066 | 0.00081259 | *traE* | conjugative transfer system protein TraE |
| ABUW_0271 | 1.62746175 | 4.8634E-06 | *-* | hypothetical protein |
| ABUW_2881 | 1.62395386 | 0.02780176 | *ureE* | urease accessory protein E |
| ABUW_2494 | 1.62313485 | 0.00186898 | *-* | permease |
| ABUW_3149 | 1.62116929 | 0.01679457 | *-* | hypothetical protein |
| ABUW_0160 | 1.6202699 | 3.242E-06 | *-* | metallo-beta-lactamase family protein |
| ABUW_0484 | 1.61711217 | 0.00011305 | *-* | hypothetical protein |
| ABUW_1959 | 1.61314381 | 0.00579633 | *-* | hypothetical protein |
| ABUW_1520 | 1.61286489 | 6.6074E-06 | *-* | hypothetical protein |
| ABUW_1313 | 1.61126505 | 1.8064E-06 | *-* | transcriptional regulator |
| ABUW_4013 | 1.61118481 | 3.44E-07 | *-* | Putative DNA binding protein |
| ABUW_3346 | 1.60989364 | 2.89E-08 | *acnA* | aconitate hydratase 1 |
| ABUW_1951 | 1.60801274 | 0.01258431 | *-* | hypothetical protein |
| ABUW_0255 | 1.60718466 | 6.83E-07 | *-* | succinyl-CoA:coenzyme A transferase |
| ABUW_2930 | 1.60668952 | 0.00018636 | *-* | 3-oxoacyl-[acyl-carrier-protein] reductase |
| ABUW_0514 | 1.60542247 | 1.10E-08 | *-* | hypothetical protein |
| ABUW_4079 | 1.60421222 | 3.5388E-05 | *-* | hypothetical protein |
| ABUW_1631 | 1.60261703 | 2.5244E-05 | *-* | hypothetical protein |
| ABUW_2477 | 1.60118034 | 0.00033508 | *caiB1* | L-carnitine dehydrogenase |
| ABUW_0118 | 1.60030671 | 2.3615E-05 | *-* | hypothetical protein |
| ABUW_2631 | 1.60021264 | 7.8567E-06 |  |  |
| ABUW_0651 | 1.59667275 | 2.9464E-05 | *-* | hypothetical protein |
| ABUW_2217 | 1.59576486 | 2.18E-08 | *-* | phosphoglycerate mutase |
| ABUW_0131 | 1.5957301 | 0.0022657 | *-* | 2-oxo-4-hydroxy-4-carboxy-5-ureidoimidazoline decarboxylase |
| ABUW_1509 | 1.59418481 | 0.01227618 | *desC* | delta-9 acyl-lipid desaturase 1 |
| ABUW_1726 | 1.58854601 | 5.0928E-06 | *-* | D-amino acid dehydrogenase 3 small subunit |
| ABUW_0667 | 1.58620108 | 3.8434E-06 | *-* | activator of HSP90 ATPase |
| ABUW_0733 | 1.58617956 | 0.00121628 | *-* | hypothetical protein |
| ABUW_2459 | 1.58407335 | 3.2735E-05 | *-* | hypothetical protein |
| ABUW_3243 | 1.58257512 | 6.38E-07 | *filC* | FilC |
| ABUW_1242 | 1.58164114 | 9.27E-07 | *rlpA* | rare lipoprotein A |
| ABUW_3384 | 1.58154294 | 2.0893E-06 | *-* | glycosyl transferase, group 1 family protein |
| ABUW_2317 | 1.57253056 | 0.00017462 | *-* | hypothetical protein |
| ABUW_3786 | 1.57069644 | 0.00090157 | *cycA2* | D-serine/D-alanine/glycine transporter |
| ABUW_1732 | 1.56979389 | 0.00701359 | *ntrC* | nitrogen metabolism transcriptional regulator, NtrC, Fis Family |
| ABUW_1289 | 1.56934989 | 0.00079958 | *-* | hypothetical protein |
| ABUW_2585 | 1.56864027 | 0.02422935 | *-* | ABC transport system substrate-binding protein |
| ABUW_4039 | 1.56315029 | 3.9118E-06 | *-* | hypothetical protein |
| ABUW_4034 | 1.56279274 | 0.02493335 |  |  |
| ABUW_0485 | 1.56074194 | 0.00035919 | *-* | oxidoreductase short-chain dehydrogenase/reductase family |
| ABUW_1713 | 1.5604618 | 4.3591E-06 | *-* | molybdopterin biosynthesis protein |
| ABUW_2921 | 1.55967748 | 3.7422E-05 | *-* | hypothetical protein |
| ABUW_3308 | 1.55394783 | 1.08E-07 | *-* | 3-hydroxyisobutyrate dehydrogenase |
| ABUW_4110 | 1.55272684 | 9.1417E-05 | *trbC* | conjugative transfer protein |
| ABUW_1328 | 1.55170909 | 0.00011587 |  |  |
| ABUW_0813 | 1.54884858 | 4.1199E-06 | *-* | hypothetical protein |
| ABUW_2316 | 1.54788564 | 2.3298E-05 | *-* | hypothetical protein |
| ABUW_0325 | 1.54689305 | 0.00010517 | *lifO* | lipase foldase |
| ABUW_1330 | 1.54665173 | 1.22E-05 | *-* | hypothetical protein |
| ABUW_3315 | 1.54579877 | 3.3723E-05 | *-* | integral membrane protein, DUF6 |
| ABUW_3138 | 1.54421845 | 2.8379E-06 | *-* | hypothetical protein |
| ABUW_2542 | 1.54373215 | 0.00061607 | *aldH* | aldehyde dehydrogenase |
| ABUW_3002 | 1.54312059 | 0.00099871 |  |  |
| ABUW_4040 | 1.54125556 | 0.02613776 | *-* | hypothetical protein |
| ABUW_3777 | 1.53845029 | 6.2716E-06 | *-* | shikimate transporter |
| ABUW_1796 | 1.53825788 | 8.3891E-06 | *-* | hypothetical protein |
| ABUW_2576 | 1.53342791 | 2.7043E-05 | *-* | hypothetical protein |
| ABUW_2882 | 1.53266037 | 0.02248498 | *ureC* | urease, alpha subunit |
| ABUW_0314 | 1.53150404 | 0.00446819 | *pilV* | type IV pilus modification protein PilV |
| ABUW_0914 | 1.53056201 | 1.81E-07 | *-* | hypothetical protein |
| ABUW_2579 | 1.52583104 | 3.77E-07 | *-* | type VI secretion protein, EvpB/family |
| ABUW_4072 | 1.52563581 | 6.9684E-05 | *-* | hypothetical protein |
| ABUW_3710 | 1.51980994 | 0.00095049 | *-* | putative acetyltransferase |
| ABUW_3616 | 1.51617481 | 5.8502E-06 | *algZ* | alginate biosynthesis protein |
| ABUW_1656 | 1.51533985 | 0.00824299 | *-* | OmpW family protein |
| ABUW_1086 | 1.51511605 | 0.00011732 | *-* | hypothetical protein |
| ABUW_1005 | 1.51481165 | 0.00021392 | *-* | hypothetical protein |
| ABUW_1483 | 1.51098509 | 4.3316E-05 | *-* | ATPase |
| ABUW_2529 | 1.51085225 | 0.00315053 | *paaB* | enoyl-CoA hydratase |
| ABUW_0263 | 1.51059737 | 4.82E-08 | *-* | hypothetical protein |
| ABUW_0437 | 1.51023985 | 0.00022847 | *-* | hypothetical protein |
| ABUW_0904 | 1.50997838 | 0.00119477 | *-* | alkaline phosphatase |
| ABUW_1634 | 1.50963106 | 0.00034142 | *-* | hypothetical protein |
| ABUW_0647 | 1.50795151 | 3.61E-08 | *-* | acetyl-CoA acetyltransferase |
| ABUW_1967 | 1.50683943 | 6.9281E-06 | *-* | short-chain dehydrogenase/reductase |
| ABUW_1916 | 1.50623875 | 2.0594E-05 | *-* | hypothetical protein |
| ABUW_2312 | 1.503416 | 2.7799E-05 | *-* | fimbrial biogenesis outer membrane usher protein |
| ABUW_2830 | 1.5029935 | 4.80E-07 | *-* | hypothetical protein |
| ABUW_0572 | 1.50289784 | 0.00162272 | *-* | phage baseplate assembly protein |
| ABUW_1952 | 1.50279515 | 0.001109 | *-* | auxin-responsive GH3-related protein |
| ABUW_0998 | 1.50016333 | 3.5155E-05 | *-* | Peptidase M20D, amidohydrolase |
| ABUW_2426 | 1.4998297 | 0.01514079 | *-* | two component transcriptional regulator, LuxR family |
| ABUW_1926 | 1.49325791 | 0.00035709 | *-* | transporter, major facilitator family |
| ABUW_1006 | 1.49305508 | 1.1245E-06 | *-* | enoyl-CoA hydratase/isomerase |
| ABUW_3573 | 1.49303754 | 2.1914E-05 | *fadA* | acetyl-CoA C-acyltransferase FadA |
| ABUW_2699 | 1.48496607 | 0.00137545 | *-* | hypothetical protein |
| ABUW_3502 | 1.48470751 | 8.97E-07 | *etfD* | electron transfer flavoprotein-ubiquinone oxidoreductase (EsvG) |
| ABUW_0317 | 1.48426027 | 4.38E-08 | *pilY* | pilus assembly protein tip-associated adhesin PilY1 |
| ABUW_2072 | 1.48258802 | 1.5745E-06 | *ppk1* | polyphosphate kinase |
| ABUW_3681 | 1.48082628 | 1.0576E-05 | *-* | class II aldolase/adducin domain protein |
| ABUW_4065 | 1.47794959 | 7.8043E-05 |  |  |
| ABUW_3801 | 1.47744922 | 0.00012235 | *-* | hypothetical protein |
| ABUW_2553 | 1.47363594 | 0.00034178 | *-* | hypothetical protein |
| ABUW_1733 | 1.47202559 | 0.00065958 | *ntrB* | signal transduction histidine kinase, nitrogen specific, NtrB |
| ABUW_2815 | 1.47106227 | 1.6599E-05 | *-* | hypothetical protein |
| ABUW_1910 | 1.46859474 | 0.00089087 | *-* | thermostable carboxypeptidase 1 |
| ABUW_1832 | 1.46856579 | 0.00476932 | *pcaJ* | 3-oxoadipate CoA-transferase subunit B |
| ABUW_4017 | 1.46676801 | 1.2837E-06 | *-* | hypothetical protein |
| ABUW_1875 | 1.46659817 | 0.00015372 | *catA* | catechol 1,2-dioxygenase |
| ABUW_2470 | 1.46649884 | 0.00020066 | *dcaH* | 3-oxoadipate CoA-transferase subunit A |
| ABUW_0020 | 1.466179 | 1.3297E-06 | *-* | glutathione S-transferase |
| ABUW_1651 | 1.46558929 | 0.03080121 | *-* | hypothetical protein |
| ABUW_1835 | 1.46361296 | 0.00322552 | *pcaD* | 3-oxoadipate enol-lactonase |
| ABUW_0970 | 1.46254687 | 0.00834602 | *-* | hypothetical protein |
| ABUW_1759 | 1.46083102 | 4.3924E-06 | *-* | extracellular serine protease |
| ABUW_3577 | 1.45807095 | 0.00041924 | *-* | hypothetical protein |
| ABUW_2377 | 1.45785797 | 0.0097764 | *-* | permease |
| ABUW_5014 | 1.4573446 | 0.00031631 | *-* | hypothetical protein |
| ABUW_1682 | 1.45282424 | 2.9339E-06 | *deb2* | phospholipase C, phosphocholine-specific |
| ABUW_1063 | 1.45175536 | 9.0723E-06 | *-* | hypothetical protein |
| ABUW_1161 | 1.45145266 | 0.00274588 | *-* | hypothetical protein |
| ABUW_1801 | 1.45079958 | 0.03539864 | *-* | hypothetical protein |
| ABUW_2155 | 1.45074452 | 5.34E-07 | *-* | hypothetical protein |
| ABUW_0721 | 1.450625 | 8.80E-07 | *wax-dgaT* | bifunctional protein wax ester synthase/acyl-CoA diacylglycerol acyltransferase |
| ABUW_1755 | 1.44297881 | 0.00175292 | *-* | transcriptional regulator, AsnC family |
| ABUW_4018 | 1.44297109 | 1.775E-05 |  |  |
| ABUW_2744 | 1.44083786 | 5.2027E-05 | *-* | putative membrane protein |
| ABUW_2591 | 1.43839058 | 0.01638482 | *uca* | urea amidolyase |
| ABUW_1949 | 1.43750984 | 0.0015747 | *-* | RND family drug transporter |
| ABUW_1932 | 1.4361394 | 1.1665E-05 | *-* | transcriptional regulator, TetR family |
| ABUW_2609 | 1.43540753 | 1.2363E-05 | *-* | transcriptional regulator, LysR-type |
| ABUW_0337 | 1.43268956 | 3.7026E-06 | *mdfA* | chloramphenicol resistance pump cmr |
| ABUW_0022 | 1.43211033 | 0.00015317 | *-* | sodium-and chloride-dependent transporter |
| ABUW_2090 | 1.43118499 | 0.0016543 | *-* | 4-hydroxybenzoate transporter |
| ABUW_2577 | 1.43053217 | 0.00433193 | *-* | type VI secretion system lysozyme-related protein |
| ABUW_4106 | 1.42791078 | 0.00080058 | *-* | hypothetical protein |
| ABUW_2157 | 1.42673768 | 0.00180825 | *-* | hypothetical protein |
| ABUW_2595 | 1.42620251 | 0.00125543 | *-* | hypothetical protein |
| ABUW_0009 | 1.42282144 | 0.00195021 | *-* | RND type efflux pump |
| ABUW_2414 | 1.42266886 | 0.01735216 | *-* | rhodanese domain protein |
| ABUW_4066 | 1.42258463 | 0.02286718 | *-* | hypothetical protein |
| ABUW_2313 | 1.4187074 | 2.6307E-05 | *-* | fimbrial protein |
| ABUW_0648 | 1.4182856 | 1.3778E-05 | *-* | type 4 fimbrial biogenesis protein FimT |
| ABUW_2575 | 1.41673175 | 2.6293E-05 | *-* | hypothetical protein |
| ABUW_2684 | 1.41479711 | 0.00301843 | *-* | phage putative head morphogenesis protein |
| ABUW_1917 | 1.41366289 | 6.4524E-05 | *-* | hypothetical protein |
| ABUW_0053 | 1.41247032 | 0.00634198 | *-* | UPF0391 membrane protein |
| ABUW_3179 | 1.41093849 | 7.74E-08 | *-* | hypothetical protein |
| ABUW_1065 | 1.4109159 | 3.5652E-05 | *-* | hypothetical protein |
| ABUW_1664 | 1.40915679 | 0.01351176 | *-* | hypothetical protein |
| ABUW_2339 | 1.40825301 | 0.00264567 | *metQ2* | D-methionine transport protein |
| ABUW_2659 | 1.40819873 | 0.00531549 | *-* | hypothetical protein |
| ABUW_0916 | 1.40717802 | 2.82E-07 | *-* | biofilm-associated protein |
| ABUW_5008 | 1.40480086 | 7.95E-07 | *-* | TonB-dependent receptor protein |
| ABUW_4045 | 1.40406708 | 0.0045851 | *-* | hypothetical protein |
| ABUW_2653 | 1.40325688 | 0.0090312 | *-* | shufflon-specific DNA recombinase |
| ABUW_1319 | 1.40286724 | 0.00016311 | *-* | hypothetical protein |
| ABUW_2159 | 1.40187184 | 2.0943E-05 | *-* | histidine triad protein |
| ABUW_0028 | 1.40160176 | 3.0172E-05 | *-* | hypothetical protein |
| ABUW_3482 | 1.39908071 | 0.00016517 | *-* | transcriptional regulator, HTH-type |
| ABUW_3458 | 1.39447326 | 4.7322E-05 | *-* | hypothetical protein |
| ABUW_1912 | 1.39164658 | 0.04308674 | *-* | hypothetical protein |
| ABUW_2939 | 1.3908466 | 1.6519E-05 | *-* | major facilitator family transporter |
| ABUW_2498 | 1.3905807 | 0.00070645 | *-* | flavoprotein oxidoreductase |
| ABUW_2420 | 1.39007973 | 0.00031362 | *-* | ABC transporter, periplasmic binding protein |
| ABUW_1817 | 1.38922308 | 6.8871E-06 | *-* | putative peroxidase |
| ABUW_0573 | 1.38271355 | 0.00081863 | *-* | phage tail completion protein |
| ABUW_0920 | 1.38187291 | 3.5832E-05 | *-* | metal-dependent hydrolase of the beta-lactamase superfamily |
| ABUW_3003 | 1.37915111 | 0.00080641 | *-* | hypothetical protein |
| ABUW_3585 | 1.37864316 | 0.00040522 | *-* | short chain dehydrogenase |
| ABUW_1960 | 1.37451269 | 2.4573E-05 | *-* | hypothetical protein |
| ABUW_3130 | 1.37282971 | 0.00068372 | *-* | transcriptional regulator, GntR family |
| ABUW_0671 | 1.37196734 | 0.00078009 | *-* | transposase |
| ABUW_4042 | 1.37048078 | 0.00104745 | *-* | hypothetical protein |
| ABUW_2560 | 1.36985916 | 0.00036672 | *-* | cyanate transport protein CynX |
| ABUW_2440 | 1.36945726 | 0.00136976 | *-* | surface antigen |
| ABUW_2806 | 1.36787816 | 0.00015673 | *-* | putrescine importer |
| ABUW_4004 | 1.36676185 | 1.0057E-05 | *-* | hypothetical protein |
| ABUW_0139 | 1.36478165 | 3.9118E-06 | *-* | hypothetical protein |
| ABUW_2396 | 1.36420098 | 0.00160783 | *-* | malonate decarboxylase, epsilon subunit |
| ABUW_2344 | 1.36367083 | 0.00030427 | *-* | transcriptional regulator, Crp/Fnr family |
| ABUW_1925 | 1.36284058 | 0.00022683 | *-* | senescence marker protein-30 |
| ABUW_0964 | 1.36035083 | 0.00431938 | *-* | hypothetical protein |
| ABUW_3654 | 1.36003253 | 0.00036038 | *amt* | ammonium transporter |
| ABUW_1476 | 1.357696 | 3.8445E-05 | *-* | alpha/beta hydrolase fold protein |
| ABUW_2679 | 1.3533579 | 0.00644392 | *-* | hypothetical protein |
| ABUW_2612 | 1.35052507 | 4.7743E-06 | *-* | hypothetical protein |
| ABUW_4116 | 1.34813418 | 0.00060997 | *-* | Lytic transglycosylase catalytic |
| ABUW_2402 | 1.34771998 | 0.00451052 | *mdcA* | malonate decarboxylase, alpha subunit |
| ABUW_2799 | 1.34723539 | 9.2671E-05 | *astA2* | arginine N-succinyltransferase |
| ABUW_1993 | 1.34089655 | 0.00511253 | *-* | hypothetical protein |
| ABUW_2969 | 1.33775164 | 0.00052175 | *-* | high-affinity choline transport protein |
| ABUW_0481 | 1.33579794 | 0.00025102 | *-* | acyl-CoA dehydrogenase |
| ABUW_2878 | 1.33529233 | 0.00877378 | *ureJ* | urease accessory protein J |
| ABUW_1954 | 1.32907954 | 0.0001108 |  |  |
| ABUW_0810 | 1.32716396 | 1.2643E-05 | *-* | hypothetical protein |
| ABUW_4096 | 1.32579564 | 0.00979451 | *-* | hypothetical protein |
| ABUW_0677 | 1.32560931 | 0.00193896 | *-* | hypothetical protein |
| ABUW_0811 | 1.32421234 | 7.7484E-06 | *-* | hypothetical protein |
| ABUW_2469 | 1.32281106 | 0.01873625 | *dcaC* | 3-oxoadipate CoA-transferase, subunit B |
| ABUW_2726 | 1.32021567 | 0.00072351 | *-* | endonuclease/exonuclease/phosphatase |
| ABUW_1643 | 1.31932382 | 0.04284536 | *-* | hypothetical protein |
| ABUW_2158 | 1.3189527 | 2.7043E-05 | *-* | hypothetical protein |
| ABUW_3775 | 1.31882085 | 0.00979451 | *-* | hypothetical protein |
| ABUW_0620 | 1.31797467 | 0.00026951 | *-* | hypothetical protein |
| ABUW_2378 | 1.31731643 | 0.00888045 | *hyu* | Asp/Glu racemase |
| ABUW_1052 | 1.31508863 | 0.00017253 | *-* | hypothetical protein |
| ABUW_1660 | 1.3146414 | 0.0005081 | *-* | ABC transporter, ATP-binding protein |
| ABUW_3242 | 1.31220094 | 3.9118E-06 | *fadL* | FilD |
| ABUW_1652 | 1.30592539 | 0.01913698 | *-* | hypothetical protein |
| ABUW_1909 | 1.30571478 | 0.00132642 | *-* | hypothetical protein |
| ABUW_0001 | 1.30444303 | 3.5506E-06 | *dnaA* | chromosomal replication initiator protein DnaA |
| ABUW_1121 | 1.30379038 | 0.00035919 | *-* | hypothetical protein |
| ABUW_0157 | 1.30178349 | 0.00019142 | *-* | hypothetical protein |
| ABUW_2733 | 1.30109839 | 0.01277444 | *-* | hypothetical protein |
| ABUW_3474 | 1.3003136 | 5.3727E-05 | *-* | transcriptional regulator, DeoR family |
| ABUW_2442 | 1.30008037 | 0.04423422 | *-* | hypothetical protein |
| ABUW_2677 | 1.29567997 | 7.9244E-05 | *-* | hypothetical protein |
| ABUW_3078 | 1.29390341 | 5.8134E-05 | *-* | transcriptional regulator, LysR family |
| ABUW_2475 | 1.2933464 | 0.00972579 | *-* | enoyl-CoA hydratase/isomerase |
| ABUW_0363 | 1.29286798 | 0.00264315 | *-* | YCII-related protein |
| ABUW_0117 | 1.29172877 | 4.7159E-06 | *-* | hypothetical protein |
| ABUW_1420 | 1.29110976 | 0.0203136 | *-* | hypothetical protein |
| ABUW_3572 | 1.29045002 | 4.5499E-05 | *fadB* | fatty oxidation complex, alpha subunit FadB |
| ABUW_2767 | 1.28997659 | 0.00806469 | *-* | monooxygenase |
| ABUW_0079 | 1.2871047 | 0.00516757 | *-* | proline-specific permease ProY |
| ABUW_0122 | 1.28595624 | 0.00028475 | *-* | PGAP1 family protein |
| ABUW_6001 | 1.28338849 | 1.6842E-05 | *-* | replication protein |
| ABUW_2626 | 1.28298565 | 3.5753E-05 | *-* | hypothetical protein |
| ABUW_3473 | 1.28113607 | 3.5388E-05 | *-* | glutathione S-transferase |
| ABUW_1836 | 1.28019003 | 0.00017828 | *pcaK* | 4-hydroxybenzoate transporter |
| ABUW_1939 | 1.27817092 | 0.0135878 | *-* | 2-hydroxy-3-oxopropionate reductase |
| ABUW_3027 | 1.27758277 | 0.00530786 | *-* | hypothetical protein |
| ABUW_2176 | 1.27689755 | 0.0159875 | *-* | hypothetical protein |
| ABUW_2394 | 1.27594873 | 0.00282839 | *madM* | malonate transporter, MadM subunit |
| ABUW_1955 | 1.27176235 | 0.01992485 | *-* | putative acid phosphatase |
| ABUW_2171 | 1.26935222 | 0.00922272 | *cobS* | cobalamin 5'-phosphate synthase |
| ABUW_3123 | 1.26349388 | 0.00016078 | *otsA* | trehalose-6-phosphate synthase |
| ABUW_4067 | 1.2628683 | 0.00486075 | *-* | hypothetical protein |
| ABUW_0038 | 1.26178645 | 0.0240187 | *-* | hypothetical protein |
| ABUW_0318 | 1.2607281 | 0.0007163 | *comE* | pilin like competence factor |
| ABUW_1695 | 1.25957284 | 0.00040065 | *-* | hypothetical protein |
| ABUW_1758 | 1.25948997 | 0.00190868 | *-* | hypothetical protein |
| ABUW_2774 | 1.25844393 | 0.00121628 | *vanB* | 2Fe-2S iron-sulfur cluster binding domain protein |
| ABUW_2656 | 1.25596799 | 9.4217E-05 | *-* | lipoprotein, putative |
| ABUW_2797 | 1.25505976 | 0.00051809 | *-* | hypothetical protein |
| ABUW_2669 | 1.25379603 | 0.03244311 | *-* | helicase domain-containing terminase-like protein |
| ABUW_1667 | 1.25319452 | 0.00548422 |  |  |
| ABUW_0516 | 1.25237559 | 0.00036733 | *-* | alpha/beta fold family hydrolase |
| ABUW_2583 | 1.25011392 | 0.00257343 | *-* | binding-protein-dependent transport systems inner membrane component |
| ABUW_1454 | 1.24886542 | 4.3409E-05 | *-* | hypothetical protein |
| ABUW_2084 | 1.24357176 | 0.0068551 | *-* | transcriptional regulator, AraC family |
| ABUW_2275 | 1.24339611 | 7.0272E-05 | *-* | transcriptional regulator, TetR family |
| ABUW_0294 | 1.24332268 | 8.6087E-06 | *comQ* | fimbrial assembly protein PilQ |
| ABUW_2949 | 1.24289322 | 0.02019393 | *-* | glyoxalase/bleomycin resistance protein/dioxygenase |
| ABUW_0814 | 1.24273523 | 0.0017406 | *-* | hypothetical protein |
| ABUW_3800 | 1.23934274 | 0.00530739 | *-* | transposase, Mutator family |
| ABUW_1994 | 1.23430586 | 1.8312E-05 | *-* | hypothetical protein |
| ABUW_2970 | 1.22621227 | 0.01106136 | *betI* | transcriptional repressor BetI |
| ABUW_1148 | 1.22287868 | 1.1728E-05 | *-* | glycine betaine transporter OpuD |
| ABUW_1944 | 1.21584857 | 4.6122E-05 | *-* | ADP-ribose pyrophosphatase |
| ABUW_1987 | 1.2130232 | 0.00031631 | *-* | hypothetical protein |
| ABUW_3311 | 1.212933 | 8.2556E-05 | *-* | short chain dehydrogenase |
| ABUW_0534 | 1.21279628 | 5.9733E-05 | *-* | LemA family |
| ABUW_2081 | 1.21231239 | 0.02836691 | *antA* | anthranilate 1,2-dioxygenase, large subunit |
| ABUW_0860 | 1.21104463 | 1.1938E-05 | *lipB* | lipoate-protein ligase B |
| ABUW_3058 | 1.20983934 | 0.00037529 | *-* | putative metal-dependent hydrolase |
| ABUW_1064 | 1.20884769 | 0.00085185 | *-* | glycosyltransferase |
| ABUW_0720 | 1.20650512 | 0.00016474 | *-* | hypothetical protein |
| ABUW_0770 | 1.20414635 | 0.00023115 | *-* | hypothetical protein |
| ABUW_4095 | 1.2037127 | 0.00246593 | *-* | Soluble lytic murein transglycosylase |
| ABUW_0498 | 1.20244512 | 0.0004104 | *-* | hypothetical protein |
| ABUW_0682 | 1.2015131 | 2.9876E-05 | *pilL* | type IV pilus hybrid sensor kinase/response regulator PilL |
| ABUW_1106 | 1.19930449 | 0.00117282 | *-* | hypothetical protein |
| ABUW_2439 | 1.19860783 | 0.02205858 | *-* | hypothetical protein |
| ABUW_0568 | 1.19677286 | 0.00012123 | *-* | phage tail fiber protein |
| ABUW_1287 | 1.19630814 | 0.01004002 | *-* | hypothetical protein |
| ABUW_1774 | 1.19476411 | 0.00020905 | *-* | hypothetical protein |
| ABUW_2138 | 1.19413128 | 4.5029E-06 | *trpB2* | tryptophan synthase, beta subunit |
| ABUW_2800 | 1.19403798 | 0.00026995 | *-* | succinylornithine transaminase |
| ABUW_1136 | 1.19052904 | 3.5386E-05 | *-* | sodium/bile acid symporter family protein |
| ABUW_1154 | 1.19026873 | 0.01105098 | *-* | hypothetical protein |
| ABUW_2200 | 1.18894679 | 0.00010082 | *-* | hypothetical protein |
| ABUW_0248 | 1.1886421 | 2.506E-05 | *-* | hypothetical protein |
| ABUW_2340 | 1.18859058 | 0.00162042 | *metN1* | D-and L-methionine transport protein |
| ABUW_2673 | 1.18624449 | 0.01041168 | *-* | hypothetical protein |
| ABUW_3492 | 1.18505527 | 0.000127 | *crcB* | CrcB protein |
| ABUW_0023 | 1.1833169 | 0.03013861 | *-* | hypothetical protein |
| ABUW_0482 | 1.18058919 | 0.00378831 | *-* | transcriptional regulator, TetR family |
| ABUW_0582 | 1.18013086 | 0.00753874 | *-* | capsid scaffolding protein |
| ABUW_0902 | 1.17824153 | 0.00379395 | *-* | hypothetical protein |
| ABUW_0725 | 1.17784183 | 0.00040504 | *-* | NAD(P)H dehydrogenase, quinone family |
| ABUW_1232 | 1.17606995 | 0.00261917 | *-* | MFS transporter, NNP family |
| ABUW_1953 | 1.17345597 | 0.00979451 | *-* | outer membrane efflux protein |
| ABUW_0480 | 1.17331684 | 0.00608066 | *-* | hypothetical protein |
| ABUW_2215 | 1.17318304 | 0.00071895 | *-* | nucleoside-diphosphate-sugar epimerase |
| ABUW_2479 | 1.1708845 | 0.00773645 | *mucK1* | cis-muconate transport protein |
| ABUW_1854 | 1.16811584 | 0.02216637 | *benP1* | benzoate transport porin BenP |
| ABUW_3372 | 1.16456011 | 0.00063545 | *-* | 4'-phosphopantetheinyl transferase |
| ABUW_3011 | 1.16413916 | 0.0499606 | *-* | hypothetical protein |
| ABUW_2947 | 1.16392309 | 0.01228975 | *-* | major facilitator superfamily MFS_1 |
| ABUW_0833 | 1.16066559 | 0.01455388 | *-* | hypothetical protein |
| ABUW_2768 | 1.16005187 | 0.02453534 | *-* | short chain dehydrogenase |
| ABUW_2879 | 1.15809277 | 0.04722218 | *ureG* | urease accessory protein G |
| ABUW_1291 | 1.15328867 | 0.03902066 | *-* | phage protein |
| ABUW_2228 | 1.14971992 | 0.00010686 | *-* | TonB-dependent receptor |
| ABUW_0795 | 1.14841995 | 0.00132144 | *-* | hypothetical protein |
| ABUW_0230 | 1.14827002 | 0.00280302 | *-* | acetyltransferase, gnat family |
| ABUW_1711 | 1.14794926 | 0.00785052 | *moaE* | molybdopterin converting factor, subunit 2 |
| ABUW_1464 | 1.14397425 | 0.0049824 | *mmsA2* | methylmalonate-semialdehyde dehydrogenase |
| ABUW_3253 | 1.14174088 | 0.00360743 | *rpoN* | RNA polymerase sigma-54 factor |
| ABUW_6003 | 1.14005979 | 1.8097E-05 | *-* | hypothetical protein |
| ABUW_2211 | 1.1376221 | 0.00127745 | *-* | hypothetical protein |
| ABUW_1908 | 1.13710125 | 6.4125E-05 | *-* | glutathione-regulated potassium-efflux system protein |
| ABUW_0812 | 1.13413301 | 2.9669E-05 | *-* | hypothetical protein |
| ABUW_1864 | 1.13407457 | 0.02988456 | *-* | 2Fe-2S iron-sulfur cluster binding domain protein |
| ABUW_2276 | 1.13203169 | 0.00099676 | *-* | transcriptional regulator, ArsR family |
| ABUW_0650 | 1.1297107 | 0.02562434 | *-* | hypothetical protein |
| ABUW_2328 | 1.12940085 | 4.7208E-05 | *-* | transcriptional regulator, AsnC family |
| ABUW_3103 | 1.127901 | 1.9207E-05 | *ribB2* | 3,4-dihydroxy-2-butanone 4-phosphate synthase |
| ABUW_1618 | 1.12776388 | 0.00735535 | *eutC* | ethanolamine ammonia-lyase, light chain |
| ABUW_2527 | 1.1267632 | 0.0024645 | *paaE* | beta-ketoadipyl CoA thiolase |
| ABUW_2349 | 1.12441013 | 0.00014656 | *-* | hypothetical protein |
| ABUW_1706 | 1.12428351 | 0.00470279 | *nasA* | molybdopterin oxidoreductase |
| ABUW_3798 | 1.12290291 | 0.00367302 | *-* | short-chain dehydrogenase/reductase SDR |
| ABUW_2951 | 1.12288543 | 0.00765954 | *-* | rieske (2Fe-2S) protein |
| ABUW_0058 | 1.11913661 | 0.00209539 | *-* | hypothetical protein |
| ABUW_3825 | 1.11837508 | 9.8369E-05 | *-* | hypothetical protein |
| ABUW_0175 | 1.11354873 | 0.01091598 | *acsA* | acetate--CoA ligase |
| ABUW_2716 | 1.11342734 | 0.00491843 | *benP2* | benzoate transport porin BenP |
| ABUW_3001 | 1.11231503 | 0.00109466 | *-* | hypothetical protein |
| ABUW_2195 | 1.11115426 | 0.00094221 | *alkM* | alkane 1-monooxygenase |
| ABUW_4033 | 1.11106741 | 0.00075218 | *-* | hypothetical protein |
| ABUW_2862 | 1.11073342 | 0.00491989 | *-* | hypothetical protein |
| ABUW_0158 | 1.11068364 | 4.7263E-05 | *-* | hypothetical protein |
| ABUW_3550 | 1.11022836 | 0.00120525 | *pilC* | pilin biogenesis protein |
| ABUW_2199 | 1.10909878 | 0.00042545 | *-* | hypothetical protein |
| ABUW_1957 | 1.10766524 | 0.00678801 | *-* | guanosine-3',5'-bis(diphosphate) 3'-pyrophosphohydrolase ((ppGpp)ase) |
| ABUW_2401 | 1.10649174 | 0.01748534 | *mdcB* | triphosphoribosyl-dephospho-CoA synthase MdcB |
| ABUW_3641 | 1.10399832 | 0.00026279 | *pilR* | type 4 fimbriae expression regulatory protein PilR |
| ABUW_3749 | 1.1036266 | 4.997E-05 | *-* | glutathione S-transferase |
| ABUW_3740 | 1.10120413 | 7.6508E-05 | *znuA* | high affinity Zn transport protein |
| ABUW_0945 | 1.10009921 | 0.00956937 | *fdhD* | formate dehydrogenase accessory protein FdhD |
| ABUW_2526 | 1.099533 | 0.00235265 | *paaF* | phenylacetate-CoA ligase |
| ABUW_4031 | 1.0970873 | 0.009191 | *-* | hypothetical protein |
| ABUW_0215 | 1.09257766 | 0.00027879 | *-* | permease |
| ABUW_2934 | 1.09095213 | 0.00026715 | *fsr* | fosmidomycin resistance protein |
| ABUW_1078 | 1.09014733 | 0.04069595 | *-* | major facilitator family transporter |
| ABUW_3787 | 1.08975988 | 0.01579226 | *-* | endoribonuclease L-PSP family protein |
| ABUW_2730 | 1.08773302 | 0.00011587 | *-* | outer membrane protein |
| ABUW_0291 | 1.08752689 | 0.01317453 | *comN* | type 4 fimbrial biogenesis protein PilN |
| ABUW_2932 | 1.08750653 | 0.01211179 | *-* | hypothetical protein |
| ABUW_1147 | 1.084623 | 0.0014183 | *-* | glycine betaine transporter OpuD |
| ABUW_3780 | 1.08393881 | 0.00263736 | *-* | acyl-CoA dehydrogenase |
| ABUW_1757 | 1.08389101 | 0.00275036 | *-* | proline-specific permease ProY |
| ABUW_2492 | 1.0822295 | 0.00119131 | *gabD2* | succinate-semialdehyde dehydrogenase (NADP+) |
| ABUW_2353 | 1.08169861 | 0.00608451 | *-* | lysine exporter protein |
| ABUW_1896 | 1.07868859 | 0.00101076 | *-* | transcriptional regulator, AraC family |
| ABUW_1870 | 1.0732412 | 0.00395572 | *-* | peptidase S45, penicillin amidase |
| ABUW_1051 | 1.07314904 | 0.00091815 | *-* | hypothetical protein |
| ABUW_0963 | 1.07274863 | 0.02272323 | *-* | glycosyltransferase |
| ABUW_2750 | 1.07056594 | 0.00886631 | *csrA* | carbon storage regulator |
| ABUW_2655 | 1.06993391 | 0.00010804 | *-* | hypothetical protein |
| ABUW_3564 | 1.06985933 | 0.0003609 | *alkB* | DNA repair system |
| ABUW_0664 | 1.06900874 | 0.00011991 | *-* | hypothetical protein |
| ABUW_1855 | 1.06894161 | 0.04758265 | *-* | general substrate transporter:Major facilitator superfamily |
| ABUW_3207 | 1.06891739 | 0.00787318 | *citA* | citrate-proton symporter |
| ABUW_1525 | 1.0685957 | 0.00056987 | *-* | C4-dicarboxylate transport protein |
| ABUW_0728 | 1.06808166 | 8.4428E-05 | *-* | hypothetical protein |
| ABUW_1574 | 1.06771118 | 0.01780112 | *-* | acyl-CoA ligase |
| ABUW_0372 | 1.06572352 | 0.00014591 | *-* | phage shock protein C |
| ABUW_2175 | 1.06283694 | 0.00068281 | *cobU* | bifunctional adenosylcobalamin biosynthesis protein CobP |
| ABUW_2062 | 1.06191417 | 0.00014604 | *-* | phospholipase D/Transphosphatidylase |
| ABUW_3784 | 1.05914026 | 0.00336097 | *-* | transcriptional regulator, LysR family |
| ABUW_1060 | 1.05848546 | 0.00042903 | *-* | hypothetical protein |
| ABUW_0227 | 1.05366268 | 0.00097545 | *-* | transcriptional regulator, MarR family |
| ABUW_2172 | 1.0513522 | 0.0011973 | *-* | histidine acid phosphatase family protein |
| ABUW_4073 | 1.05049129 | 0.00515159 | *-* | hypothetical protein |
| ABUW_1654 | 1.05012341 | 0.02155671 | *-* | acetyltransferase, gnat family |
| ABUW_0098 | 1.04692722 | 9.0993E-05 | *-* | hypothetical protein |
| ABUW_3583 | 1.04630973 | 0.01281234 | *-* | putative outer membrane protein W |
| ABUW_3288 | 1.04496015 | 0.00012825 | *-* | glutathione S-transferase |
| ABUW_3728 | 1.04470493 | 0.00047492 | *-* | transcriptional regulator AraC family |
| ABUW_1712 | 1.04460156 | 0.00558996 | *moaCB* | molybdenum cofactor biosynthesis protein C |
| ABUW_0055 | 1.04271937 | 0.00059563 | *-* | glucose sorbosone dehydrogenase |
| ABUW_3399 | 1.03931663 | 0.00141147 | *-* | hypothetical protein |
| ABUW_0638 | 1.03894502 | 0.00193896 | *-* | acetyl-CoA acetyltransferase |
| ABUW_2842 | 1.03868353 | 6.123E-05 | *-* | hypothetical protein |
| ABUW_2835 | 1.03444833 | 0.00144728 | *-* | oxidoreductase, FAD/FMN-binding |
| ABUW_2902 | 1.03418987 | 0.00197704 | *rubA* | rubredoxin |
| ABUW_2374 | 1.03382425 | 0.02392406 | *-* | isochorismatase hydrolase |
| ABUW_1867 | 1.02929222 | 0.0068551 | *tynA* | copper amine oxidase |
| ABUW_4076 | 1.02885658 | 0.00029453 | *trwC* | TrwC protein |
| ABUW_2594 | 1.02685568 | 0.00974786 | *-* | glutathione-dependent formaldehyde dehydrogenase |
| ABUW_0919 | 1.02679322 | 0.00530786 | *dgkA* | diacylglycerol kinase |
| ABUW_0290 | 1.02639104 | 0.00033418 | *-* | type IV pilus assembly protein PilM |
| ABUW_3779 | 1.02456046 | 0.00283093 | *-* | short-chain enoyl-CoA hydratase |
| ABUW_3336 | 1.0238236 | 0.01339583 | *-* | hypothetical protein |
| ABUW_0064 | 1.0202839 | 0.00486459 | *-* | MATE family drug transporter |
| ABUW_1637 | 1.01945688 | 0.00471091 | *-* | oxidoreductase short-chain dehydrogenase/reductase family |
| ABUW_3287 | 1.0187596 | 0.0043675 | *-* | hypothetical protein |
| ABUW_3891 | 1.01757129 | 0.00098215 | *-* | integral membrane protein TerC |
| ABUW_1212 | 1.016715 | 0.01422475 | *-* | hypothetical protein |
| ABUW_1764 | 1.01415617 | 8.5705E-05 | *-* | GGDEF family protein |
| ABUW_2385 | 1.0139567 | 0.00405059 | *-* | monooxygenase, NtaA/SnaA/SoxA family |
| ABUW_2462 | 1.01259116 | 0.04724038 | *-* | major facilitator superfamily MFS_1 |
| ABUW_2610 | 1.01237033 | 0.00048811 | *-* | hypothetical protein |
| ABUW_1061 | 1.01174915 | 0.00077708 | *wecB* | UDP-N-acetylglucosamine 2-epimerase |
| ABUW_0358 | 1.0087646 | 0.00240776 | *-* | peptidase S8 and S53 subtilisin |
| ABUW_3560 | 1.00631732 | 0.00073768 | *-* | HlyD family secretion protein |
| ABUW_1616 | 1.00325963 | 0.00175033 | *-* | hypothetical protein |
| ABUW_3778 | 1.00322464 | 0.00047954 | *-* | enoyl-CoA hydratase/isomerase family protein |
| ABUW_3378 | -1.0007815 | 0.00043396 | *-* | hypothetical protein |
| ABUW_2150 | -1.0009422 | 0.0001531 | *metZ* | O-succinylhomoserine sulfhydrylase |
| ABUW_3409 | -1.0030895 | 7.8056E-05 | *rdgB* | non-canonical purine NTP pyrophosphatase, HAM1 family |
| ABUW_1547 | -1.0039965 | 0.01368099 | *rpsR* | ribosomal protein S18 |
| ABUW_3043 | -1.0044752 | 0.00092004 | *rep* | ATP-dependent DNA helicase |
| ABUW_2285 | -1.0047148 | 7.8506E-05 | *putP* | sodium/proline symporter |
| ABUW_2269 | -1.0052162 | 0.00127774 | *fba* | fructose-bisphosphate aldolase, class II, Calvin cycle subtype |
| ABUW_0426 | -1.0067233 | 0.00153651 | *secY* | preprotein translocase, SecY subunit |
| ABUW_0641 | -1.0073781 | 0.00034045 | *-* | porin B |
| ABUW_0040 | -1.0078014 | 0.00035977 | *-* | hypothetical protein |
| ABUW_1012 | -1.0100809 | 0.00193896 | *-* | ExsB protein |
| ABUW_0389 | -1.0118737 | 0.00012761 | *-* | hypothetical protein |
| ABUW_1744 | -1.0127268 | 0.00083327 | *fabZ* | beta-hydroxyacyl-(acyl-carrier-protein) dehydratase |
| ABUW_3382 | -1.014024 | 0.00085513 | *-* | hypothetical protein |
| ABUW_3732 | -1.0165437 | 0.0012331 | *atpD* | ATP synthase F1, beta subunit |
| ABUW_1559 | -1.0170903 | 0.00165208 | *pgaC* | glycosyl transferase, family 2 protein |
| ABUW_3038 | -1.0171957 | 0.00233328 | *-* | glutamine amidotransferase, class-II |
| ABUW_0056 | -1.0180263 | 0.00015004 | *-* | ATPase, AAA family |
| ABUW_0331 | -1.0188385 | 0.00272541 | *serA* | D-3-phosphoglycerate dehydrogenase |
| ABUW_0422 | -1.0203418 | 0.00306725 | *rplR* | ribosomal protein L18 |
| ABUW_3829 | -1.0207205 | 0.00040522 | *-* | hypothetical protein |
| ABUW_0305 | -1.0217953 | 0.00020226 | *-* | O-antigen polymerase family |
| ABUW_3304 | -1.0239579 | 0.00167797 | *-* | hypothetical protein |
| ABUW_1244 | -1.0240027 | 0.00103808 | *mrdB* | rod shape-determining protein RodA (EsvE3) |
| ABUW_3250 | -1.0247192 | 0.0002851 | *murA* | UDP-N-acetylglucosamine 1-carboxyvinyltransferase |
| ABUW_2755 | -1.0265276 | 0.00108323 | *-* | xanthine/uracil permease |
| ABUW_3746 | -1.0277873 | 0.00013228 | *sfcA* | NAD-dependent malic enzyme |
| ABUW_3116 | -1.0283265 | 0.00069291 | *-* | ribosomal large subunit pseudouridine synthase B |
| ABUW_0268 | -1.0289908 | 0.0001789 | *czcA* | cation efflux system protein |
| ABUW_0938 | -1.0297102 | 0.03782928 |  |  |
| ABUW_1568 | -1.0372746 | 0.00382973 | *hemK* | protein methylase, HemK family |
| ABUW_0124 | -1.0399441 | 5.335E-05 | *parE* | DNA topoisomerase IV, B subunit |
| ABUW_1639 | -1.0422918 | 0.00142968 | *-* | transcriptional regulator, AsnC family |
| ABUW_3466 | -1.0437684 | 0.00014985 | *-* | HAD-superfamily subfamily IB hydrolase |
| ABUW_3040 | -1.0452509 | 3.2078E-05 | *argB* | acetylglutamate kinase |
| ABUW_0425 | -1.0484959 | 0.00100116 | *rplO* | ribosomal protein L15 |
| ABUW_3048 | -1.0486843 | 2.424E-05 | *minC* | septum site-determining protein MinC |
| ABUW_0491 | -1.0488269 | 0.00024596 | *sspB* | stringent starvation protein B |
| ABUW_2660 | -1.049927 | 0.00030247 | *-* | hypothetical protein |
| ABUW_1110 | -1.0507342 | 0.0033903 | *ddc* | diaminobutyrate decarboxylase |
| ABUW_1615 | -1.0509321 | 0.0001792 | *glnS* | glutaminyl-tRNA synthetase |
| ABUW_2801 | -1.0522775 | 0.00597793 | *-* | transcriptional regulator, AsnC family |
| ABUW_0467 | -1.0539446 | 0.00067214 | *mdh* | malate dehydrogenase |
| ABUW_2148 | -1.0572845 | 0.00028458 | *-* | hypothetical protein |
| ABUW_2136 | -1.0573918 | 0.00185573 | *aroC* | chorismate synthase |
| ABUW_3755 | -1.0589761 | 0.00278563 | *guaA* | GMP synthase |
| ABUW_2274 | -1.0596006 | 8.7111E-05 | *-* | cupin family protein |
| ABUW_0547 | -1.059757 | 0.0002383 | *-* | hypothetical protein |
| ABUW_0989 | -1.0602256 | 0.00208991 | *-* | tRNA/rRNA methyltransferase |
| ABUW_3134 | -1.0619796 | 0.00011226 | *ffh* | signal recognition particle protein |
| ABUW_1611 | -1.0627594 | 0.00263736 | *nfnB* | oxygen-insensitive NAD(P)H nitroreductase |
| ABUW_1073 | -1.0651678 | 4.7276E-05 | *-* | pseudouridine synthase, Rsu |
| ABUW_3296 | -1.0652126 | 4.4611E-05 | *panB* | 3-methyl-2-oxobutanoate hydroxymethyltransferase |
| ABUW_3555 | -1.0671413 | 0.00036485 | *-* | hypothetical protein |
| ABUW_0612 | -1.0671714 | 4.6066E-05 | *-* | hypothetical protein |
| ABUW_3028 | -1.0707591 | 0.00020445 | *sbcD* | ATP-dependent dsDNA exonuclease |
| ABUW_3596 | -1.0736722 | 0.00513951 | *secE* | preprotein translocase, SecE subunit |
| ABUW_3376 | -1.0741058 | 0.00053582 | *hisS* | histidyl-tRNA synthetase |
| ABUW_1370 | -1.0747214 | 0.00010437 | *-* | hydrolase |
| ABUW_3148 | -1.0747865 | 0.00102594 | *metG* | methionyl-tRNA synthetase |
| ABUW_3862 | -1.0765125 | 0.00025486 | *ileS* | isoleucyl-tRNA synthetase |
| ABUW_1813 | -1.0794714 | 3.9794E-05 | *clpS* | ATP-dependent Clp protease adaptor protein |
| ABUW_1354 | -1.084151 | 0.00029254 | *-* | two-component system sensor protein |
| ABUW_1797 | -1.0846014 | 0.00058569 | *-* | hypothetical protein |
| ABUW_2262 | -1.0861323 | 6.823E-05 | *spoOJ* | chromosome partitioning protein ParB |
| ABUW_0633 | -1.0917788 | 7.9403E-05 | *-* | putative methyltransferase |
| ABUW_3173 | -1.092748 | 0.00013514 | *nuoF* | NADH dehydrogenase I chain F |
| ABUW_1218 | -1.0947961 | 1.109E-05 | *algW* | peptidase S1 and S6 |
| ABUW_0741 | -1.0959053 | 0.00099013 | *-* | hypothetical protein |
| ABUW_3363 | -1.0961884 | 0.00020599 | *macB* | macrolide export ATP-binding/permease protein MacB |
| ABUW_3084 | -1.0961982 | 0.00064782 | *-* | NADPH-dependent fmn reductase |
| ABUW_3885 | -1.0970681 | 0.00446028 | *purK* | phosphoribosylaminoimidazole carboxylase, ATPase subunit |
| ABUW_1010 | -1.1017647 | 5.0453E-05 | *-* | ATPase |
| ABUW_2277 | -1.102386 | 0.00067944 | *sstT* | inner membrane symporter YgjU |
| ABUW_0266 | -1.1034468 | 0.00045372 | *czcC* | cation efflux system protein |
| ABUW_2268 | -1.1043018 | 1.2363E-05 | *surA* | PpiC-type peptidyl-prolyl cis-trans isomerase |
| ABUW_0025 | -1.1051661 | 1.6125E-05 | *fmt* | methionyl-tRNA formyltransferase |
| ABUW_1247 | -1.1068523 | 0.00016378 | *-* | ABC transporter permease protein |
| ABUW_3174 | -1.107077 | 8.4486E-05 | *nuoE* | NADH dehydrogenase I chain E |
| ABUW_0423 | -1.1076369 | 0.00088674 | *rpsE* | ribosomal protein S5 |
| ABUW_3176 | -1.1081787 | 6.27E-05 | *nuoB* | NADH dehydrogenase I chain B |
| ABUW_0326 | -1.1128792 | 7.8043E-05 | *truB* | tRNA pseudouridine synthase B |
| ABUW_0853 | -1.114602 | 0.00027688 | *cobA* | uroporphyrin-III C-methyltransferase |
| ABUW_0625 | -1.1160059 | 9.4559E-06 | *-* | hypothetical protein |
| ABUW_3168 | -1.1164246 | 2.1112E-05 | *nuoK* | NADH dehydrogenase I chain K |
| ABUW_1698 | -1.1224871 | 0.00316118 | *aroQ* | 3-dehydroquinate dehydratase, type II |
| ABUW_0332 | -1.1244272 | 2.3258E-05 | *-* | FAD linked oxidase domain protein |
| ABUW_1781 | -1.1255199 | 0.00019293 | *-* | glutaredoxin-related protein |
| ABUW_0893 | -1.1261031 | 0.00080085 | *carB* | carbamoyl-phosphate synthase, large subunit |
| ABUW_3169 | -1.1291717 | 3.2078E-05 | *nuoJ* | NADH dehydrogenase I chain J |
| ABUW_3109 | -1.1295206 | 0.00089708 | *fabD* | malonyl CoA-acyl carrier protein transacylase |
| ABUW_3417 | -1.1300816 | 6.1568E-06 | *lgt* | prolipoprotein diacylglyceryl transferase |
| ABUW_0531 | -1.1318219 | 0.00012846 | *secD* | protein-export membrane protein SecD |
| ABUW_3177 | -1.1323378 | 1.7845E-05 | *nuoA* | NADH dehydrogenase I chain A |
| ABUW_2246 | -1.1340909 | 2.0761E-05 | *-* | putative enoyl-CoA hydratase/isomerase |
| ABUW_2190 | -1.1351784 | 0.00133318 | *-* | hypothetical protein |
| ABUW_0289 | -1.1373628 | 2.0801E-05 | *-* | penicillin-binding protein 1A |
| ABUW_1553 | -1.1378429 | 8.4486E-05 | *cyoA* | cytochrome O ubiquinol oxidase subunit II |
| ABUW_1539 | -1.1390428 | 3.055E-05 | *-* | transcriptional factor |
| ABUW_1596 | -1.1398608 | 0.00036886 | *-* | MFS superfamily permease |
| ABUW_0369 | -1.1403383 | 0.00019051 | *-* | hypothetical protein |
| ABUW_0390 | -1.1404781 | 1.1244E-05 | *-* | hydrolase, NUDIX family protein |
| ABUW_1592 | -1.1409723 | 0.00010805 | *-* | hypothetical protein |
| ABUW_0141 | -1.1431084 | 1.6673E-06 | *xth* | exodeoxyribonuclease III |
| ABUW_0418 | -1.1431479 | 0.0004415 | *rplE* | ribosomal protein L5 |
| ABUW_3030 | -1.1451935 | 0.00101431 | *-* | alanine racemase domain-containing protein |
| ABUW_3175 | -1.1454567 | 0.0001412 | *nuoCD* | NADH dehydrogenase I chain CD |
| ABUW_0424 | -1.1478353 | 0.00097387 | *rpmD* | ribosomal protein L30 |
| ABUW_0430 | -1.1505461 | 0.00076989 | *rpsD* | ribosomal protein S4 |
| ABUW_2352 | -1.1519458 | 5.8502E-06 | *-* | aminotransferase |
| ABUW_3837 | -1.1523137 | 4.9987E-05 | *ampD* | N-acetylmuramoyl-L-alanine amidase, family 2 |
| ABUW_2743 | -1.1523156 | 2.94E-06 | *-* | peptidase M48 family protein |
| ABUW_3690 | -1.1525763 | 3.0705E-06 | *parC* | DNA topoisomerase IV, A subunit |
| ABUW_1352 | -1.1534414 | 0.00283932 | *-* | hypothetical protein |
| ABUW_2432 | -1.1536882 | 0.02001227 | *-* | hypothetical protein |
| ABUW_2729 | -1.1548427 | 0.00025306 | *-* | hypothetical protein |
| ABUW_2383 | -1.1576039 | 8.3763E-05 | *-* | hypothetical protein |
| ABUW_0388 | -1.1578313 | 1.919E-05 | *corA* | magnesium and cobalt transport protein |
| ABUW_0538 | -1.1588986 | 0.00423929 | *-* | integrase |
| ABUW_1119 | -1.1590814 | 0.00025722 | *-* | peptidase S49 |
| ABUW_1124 | -1.1592296 | 0.00020796 | *trmU* | tRNA (5-methylaminomethyl-2-thiouridylate)-methyltransferase |
| ABUW_1365 | -1.1625698 | 1.9715E-05 | *pheA* | chorismate mutase |
| ABUW_0470 | -1.162729 | 0.00011912 | *-* | oxygenase |
| ABUW_3362 | -1.1632887 | 6.3012E-05 | *-* | efflux transporter, RND family, MFP subunit |
| ABUW_2287 | -1.1651128 | 0.01387638 | *putA* | delta-1-pyrroline-5-carboxylate dehydrogenase |
| ABUW_0841 | -1.1668596 | 0.00061632 | *-* | hypothetical protein |
| ABUW_0163 | -1.1695084 | 2.0361E-05 | *glmM* | phosphoglucosamine mutase |
| ABUW_1777 | -1.1713834 | 0.00048984 | *brnQ* | branched-chain amino acid transport system II carrier protein |
| ABUW_0442 | -1.1725601 | 0.0003601 | *-* | hypothetical protein |
| ABUW_0386 | -1.1725973 | 1.0297E-05 | *mlaC* | toluene tolerance protein |
| ABUW_3445 | -1.1732989 | 9.7402E-06 | *uup* | ABC transporter ATP-binding protein uup |
| ABUW_0041 | -1.1733575 | 2.7421E-05 | *-* | major facilitator family transporter |
| ABUW_2776 | -1.173553 | 0.00480167 | *hcaR* | transcriptional regulator, MarR-family |
| ABUW_1856 | -1.174154 | 0.00043269 | *-* | transcriptional regulator, MarR-family |
| ABUW_2358 | -1.1747112 | 0.00071993 | *-* | proton/sodium-glutamate symport protein |
| ABUW_2867 | -1.1754255 | 2.0496E-05 | *-* | extradiol ring-cleavage dioxygenase, class III enzyme, subunit B |
| ABUW_3841 | -1.1790309 | 8.3148E-06 | *rph* | ribonuclease PH |
| ABUW_0715 | -1.1795069 | 4.7208E-05 | *mreB* | rod shape-determining protein MreB |
| ABUW_3428 | -1.1795893 | 8.876E-06 | *-* | transcriptional regulator, TetR family |
| ABUW_3737 | -1.1805193 | 0.0005504 | *atpE* | ATP synthase F0, C subunit |
| ABUW_3172 | -1.1847712 | 4.7208E-05 | *nuoG* | NADH dehydrogenase I chain G |
| ABUW_3513 | -1.186782 | 1.9685E-05 | *hslO* | heat shock protein 33 |
| ABUW_3897 | -1.187794 | 1.9603E-05 | *hpt* | hypoxanthine phosphoribosyltransferase |
| ABUW_3278 | -1.1901352 | 7.0854E-05 | *-* | hypothetical protein |
| ABUW_3062 | -1.1910933 | 0.00027318 | *rpsG* | ribosomal protein S7 |
| ABUW_3617 | -1.1913242 | 4.5345E-06 | *argH* | argininosuccinate lyase |
| ABUW_0911 | -1.1919816 | 6.8427E-05 | *glpK* | glycerol kinase |
| ABUW_1530 | -1.1940746 | 1.2643E-05 | *-* | ABC transporter, methionine-binding protein |
| ABUW_0432 | -1.1947623 | 0.00024462 | *rplQ* | ribosomal protein L17 |
| ABUW_3747 | -1.1954219 | 3.2221E-05 | *argS* | arginyl-tRNA synthetase |
| ABUW_3584 | -1.1964297 | 1.39E-05 | *-* | Na+/H+ antiporter NhaC |
| ABUW_3570 | -1.1978092 | 3.055E-05 | *lpxO* | beta-hydroxylase |
| ABUW_3734 | -1.1980698 | 0.0001986 | *atpA* | ATP synthase F1, alpha subunit |
| ABUW_1234 | -1.1992885 | 1.0832E-05 | *map1* | methionine aminopeptidase, type I |
| ABUW_0654 | -1.1998107 | 0.0011333 | *-* | uracil-xanthine permease |
| ABUW_3427 | -1.201051 | 3.6361E-05 | *purU* | formyltetrahydrofolate deformylase |
| ABUW_2263 | -1.2010837 | 1.8445E-06 | *soj* | sporulation initiation inhibitor protein soj |
| ABUW_3557 | -1.201122 | 2.9378E-06 | *recN* | DNA repair protein RecN |
| ABUW_1734 | -1.2027033 | 3.3177E-05 | *-* | hypothetical protein |
| ABUW_0237 | -1.2049262 | 2.2946E-06 | *aqpZ* | aquaporin Z |
| ABUW_1246 | -1.2070992 | 1.747E-05 | *queF* | NADPH-dependent 7-cyano-7-deazaguanine reductase (EsvE1) |
| ABUW_0427 | -1.2079078 | 0.00011222 | *rpmJ* | ribosomal protein L36 |
| ABUW_0992 | -1.2106964 | 1.0372E-06 | *pal* | peptidoglycan-associated lipoprotein |
| ABUW_1126 | -1.2132174 | 0.00040333 | *-* | putative transferase |
| ABUW_3708 | -1.2142745 | 0.00450573 | *-* | transglycosylase SLT domain protein |
| ABUW_2265 | -1.2151611 | 4.7704E-05 | *-* | nucleotidyl transferase family protein |
| ABUW_0024 | -1.2164423 | 1.7839E-05 | *sun* | ribosomal RNA small subunit methyltransferase B |
| ABUW_3697 | -1.2179486 | 5.8363E-06 | *-* | inner membrane transport protein YieO |
| ABUW_3828 | -1.2187683 | 4.0052E-05 | *-* | hypothetical protein |
| ABUW_0643 | -1.2196951 | 0.00342777 | *cysI* | sulfite reductase |
| ABUW_3145 | -1.221087 | 2.3752E-06 | *mtnN* | 5'-methylthioadenosine/S-adenosylhomocysteine nucleosidase |
| ABUW_3736 | -1.222856 | 0.00031588 | *atpF* | ATP synthase F0, B subunit |
| ABUW_3274 | -1.2235921 | 8.46E-07 | *accA* | acetyl-CoA carboxylase, carboxyl transferase, alpha subunit |
| ABUW_1799 | -1.2237826 | 1.3848E-05 | *-* | sugar kinase, ribokinase family |
| ABUW_0686 | -1.2247496 | 2.8819E-05 | *dapE* | succinyl-diaminopimelate desuccinylase |
| ABUW_0716 | -1.2252485 | 0.00033606 | *mreC* | rod shape-determining protein MreC |
| ABUW_0097 | -1.2272909 | 2.7646E-06 | *serB* | phosphoserine phosphatase |
| ABUW_2296 | -1.2275785 | 7.2084E-05 | *metK* | methionine adenosyltransferase |
| ABUW_0563 | -1.2292978 | 7.8056E-05 | *-* | beta-lactamase OXA-23 |
| ABUW_0994 | -1.2293604 | 0.00032373 | *tolA* | tolerance to group A colicins single-stranded filamentous DNA phage |
| ABUW_0173 | -1.2305775 | 1.3679E-06 | *-* | MscS Mechanosensitive ion channel |
| ABUW_0742 | -1.2325721 | 0.00014591 | *-* | hypothetical protein |
| ABUW_3128 | -1.2378031 | 0.00015722 | *zipA* | cell division protein ZipA |
| ABUW_0865 | -1.2385158 | 0.00089961 | *-* | rhodanese domain protein |
| ABUW_3630 | -1.2385409 | 4.04E-07 | *murD* | UDP-N-acetylmuramoylalanine--D-glutamate ligase |
| ABUW_2116 | -1.239408 | 1.4761E-05 | *-* | fmn-dependent NADH-azoreductase |
| ABUW_3267 | -1.2394587 | 6.6007E-06 | *-* | hypothetical protein |
| ABUW_3170 | -1.2408272 | 3.0531E-06 | *nuoI* | NADH dehydrogenase I chain I 2Fe-2S ferredoxin-related |
| ABUW_0416 | -1.2439256 | 8.6794E-05 | *rplN* | ribosomal protein L14 |
| ABUW_2896 | -1.2451754 | 0.00338565 | *cysD* | sulfate adenylyltransferase subunit 2 |
| ABUW_0981 | -1.2488845 | 0.00013058 | *purM* | phosphoribosylformylglycinamidine cyclo-ligase |
| ABUW_0431 | -1.250913 | 0.00022226 | *rpoA* | DNA-directed RNA polymerase, alpha subunit |
| ABUW_3733 | -1.253707 | 3.2221E-05 | *atpG* | ATP synthase F1, gamma subunit AtpG |
| ABUW_1023 | -1.2582706 | 6.59E-07 | *-* | aminodeoxychorismate lyase |
| ABUW_3151 | -1.2598348 | 0.00019371 | *-* | transcriptional regulator TetR/AcrR family |
| ABUW_2788 | -1.2629705 | 0.00013929 | *kdgD* | 5-dehydro-4-deoxyglucarate dehydratase |
| ABUW_3667 | -1.2659441 | 2.103E-05 | *arsC* | arsenate reductase |
| ABUW_3046 | -1.2676829 | 1.5469E-06 | *-* | phospholipid/glycerol acyltransferase |
| ABUW_2363 | -1.2705037 | 3.4037E-06 | *-* | hypothetical protein |
| ABUW_0089 | -1.2706556 | 4.5643E-05 | *glmS* | glutamine-fructose-6-phosphate transaminase (isomerizing) |
| ABUW_2965 | -1.2742638 | 0.00207838 | *-* | hypothetical protein |
| ABUW_0941 | -1.2783223 | 4.5932E-05 | *-* | oxidoreductase short chain dehydrogenase/reductase family |
| ABUW_3748 | -1.2820005 | 6.22E-07 | *-* | hypothetical protein |
| ABUW_0525 | -1.2822928 | 2.9194E-06 | *ispZ* | intracellular septation protein A |
| ABUW_0407 | -1.2840503 | 0.00253572 | *rplD* | ribosomal protein L4/L1e |
| ABUW_1002 | -1.2850954 | 0.00196111 | *purL* | phosphoribosylformylglycinamidine synthase |
| ABUW_0327 | -1.2859275 | 3.8661E-05 | *-* | hypothetical protein |
| ABUW_3295 | -1.2862135 | 1.60E-07 | *panC* | pantoate--beta-alanine ligase |
| ABUW_0164 | -1.2862968 | 4.04E-07 | *pdxH* | pyridoxamine 5'-phosphate oxidase |
| ABUW_3060 | -1.2877202 | 7.3812E-06 | *tuf* | translation elongation factor Tu |
| ABUW_0995 | -1.2889687 | 0.00010004 | *tolR* | protein TolR |
| ABUW_2899 | -1.2918267 | 3.1195E-05 | *lysS* | lysyl-tRNA synthetase |
| ABUW_2206 | -1.2938116 | 0.00013837 | *hscA* | Fe-S protein assembly chaperone HscA |
| ABUW_0852 | -1.2942988 | 1.2613E-06 | *serS* | seryl-tRNA synthetase |
| ABUW_0714 | -1.2964638 | 1.61E-07 | *gatC* | glutamyl-tRNA(Gln) amidotransferase, C subunit |
| ABUW_0429 | -1.2974858 | 0.00010347 | *rpsK* | ribosomal protein S11 |
| ABUW_2271 | -1.2977714 | 0.00093316 | *pgk* | phosphoglycerate kinase |
| ABUW_1826 | -1.2982334 | 7.2693E-05 | *kdsA* | 2-dehydro-3-deoxyphosphooctonate aldolase |
| ABUW_2936 | -1.2994264 | 9.9838E-06 | *-* | glutaminase |
| ABUW_0249 | -1.3037282 | 1.4517E-06 | *hisH* | imidazole glycerol phosphate synthase, glutamine amidotransferase subunit |
| ABUW_0940 | -1.305396 | 4.8225E-06 | *-* | nitroreductase family protein |
| ABUW_0395 | -1.3062912 | 3.4545E-06 | *plsB* | glycerol-3-phosphate acyltransferase |
| ABUW_0259 | -1.3069697 | 2.3347E-05 | *-* | sulfate transporter |
| ABUW_3061 | -1.307012 | 6.7507E-05 | *fusA* | translation elongation factor G |
| ABUW_0146 | -1.307269 | 6.33E-07 | *murG* | undecaprenyldiphospho-muramoylpentapeptide beta-N-acetylglucosaminyltransferase |
| ABUW_2825 | -1.3086176 | 3.64E-07 | *pyrC* | dihydroorotase, homodimeric type |
| ABUW_3890 | -1.3108126 | 1.8304E-05 | *-* | PAP2 superfamily protein |
| ABUW_2866 | -1.312797 | 0.00015055 | *-* | SPOUT methyltransferase |
| ABUW_1248 | -1.3154004 | 1.1821E-05 | *-* | ABC transporter ATP-binding protein |
| ABUW_3063 | -1.3154094 | 5.8061E-05 | *rpsL* | ribosomal protein S12 |
| ABUW_3672 | -1.3162042 | 1.34E-07 | *trxB2* | thioredoxin-disulfide reductase |
| ABUW_3676 | -1.3224897 | 1.1715E-06 | *tniC* | transposition helper protein C |
| ABUW_1122 | -1.3234013 | 0.00029769 | *purB* | adenylosuccinate lyase |
| ABUW_1390 | -1.3295477 | 0.02263331 | *-* | hypothetical protein |
| ABUW_2278 | -1.3333008 | 0.00020862 | *-* | permease |
| ABUW_1013 | -1.3349568 | 0.00094472 | *-* | radical SAM domain protein |
| ABUW_2682 | -1.3352498 | 0.00320517 | *-* | transcriptional regulator, MarR-family |
| ABUW_0347 | -1.3357558 | 2.9193E-05 | *-* | amino-acid permease |
| ABUW_0630 | -1.3368275 | 6.45E-07 | *-* | hypothetical protein |
| ABUW_2114 | -1.3377243 | 0.0328646 | *-* | transcriptional regulator, GntR family |
| ABUW_0443 | -1.3386428 | 3.4545E-06 | *prlC* | oligopeptidase A |
| ABUW_0421 | -1.342946 | 3.9287E-05 | *rplF* | ribosomal protein L6 |
| ABUW_0420 | -1.3448965 | 2.3753E-05 | *rpsH* | ribosomal protein S8 |
| ABUW_0823 | -1.3471962 | 9.96E-07 | *-* | hypothetical protein |
| ABUW_1736 | -1.3499582 | 2.524E-06 | *frr* | ribosome recycling factor |
| ABUW_0628 | -1.3572043 | 0.00018797 | *-* | peroxidase |
| ABUW_3699 | -1.3583232 | 5.3768E-06 | *dinP* | DNA polymerase IV |
| ABUW_0712 | -1.3672857 | 1.5592E-05 | *gatB* | glutamyl-tRNA(Gln) amidotransferase, B subunit |
| ABUW_3300 | -1.368122 | 1.5896E-05 | *-* | MazG family protein |
| ABUW_4053 | -1.3682011 | 0.0420443 |  |  |
| ABUW_0527 | -1.369224 | 2.4366E-06 | *-* | hypothetical protein |
| ABUW_0458 | -1.3714565 | 1.8488E-06 | *xpt* | xanthine phosphoribosyltransferase |
| ABUW_0419 | -1.3722997 | 1.6246E-05 | *rpsN* | ribosomal protein S14 |
| ABUW_2870 | -1.372951 | 8.7111E-05 | *-* | electron transport complex, rnfaBcdge type, B subunit |
| ABUW_0996 | -1.3730564 | 1.62E-07 | *tolQ* | protein TolQ |
| ABUW_1973 | -1.3739759 | 0.00471664 | *adeR* | AdeR |
| ABUW_0366 | -1.373986 | 0.00020933 | *glyS* | glycyl-tRNA synthetase, beta subunit |
| ABUW_1825 | -1.3739862 | 6.4122E-06 | *pyrG* | CTP synthase |
| ABUW_3391 | -1.3804457 | 4.58E-08 | *gntK* | shikimate kinase |
| ABUW_0840 | -1.3809597 | 0.00014824 | *-* | hypothetical protein |
| ABUW_0406 | -1.3821541 | 0.00132107 | *rplC* | ribosomal protein L3 |
| ABUW_3129 | -1.3852624 | 1.33E-07 | *smc* | chromosome segregation protein SMC |
| ABUW_1224 | -1.3852934 | 1.433E-05 | *ahcY* | adenosylhomocysteinase |
| ABUW_0910 | -1.3915206 | 2.24E-07 | *kgtP* | MFS permease |
| ABUW_1567 | -1.3922916 | 2.43E-07 | *prfA* | peptide chain release factor 1 |
| ABUW_1400 | -1.3927869 | 2.5712E-05 | *-* | beta-ribbon domain peptidase S24/S26A/S26B/S26C |
| ABUW_3815 | -1.394055 | 1.87E-07 | *manB* | phosphomannomutase |
| ABUW_3735 | -1.3941132 | 1.8924E-05 | *atpH* | ATP synthase F1, delta subunit |
| ABUW_3686 | -1.3950806 | 6.22E-07 | *ppa* | inorganic diphosphatase |
| ABUW_3886 | -1.3969256 | 0.00020862 | *purE* | phosphoribosylaminoimidazole carboxylase, catalytic subunit |
| ABUW_3127 | -1.3972243 | 5.45E-08 | *ligA* | DNA ligase, NAD-dependent |
| ABUW_0622 | -1.4001136 | 2.81E-08 | *trpA* | tryptophan synthase, alpha subunit |
| ABUW_0855 | -1.4005423 | 0.00178959 | *-* | transcriptional regulator, PadR family |
| ABUW_0147 | -1.4037332 | 6.4122E-06 | *murC* | UDP-N-acetylmuramate--alanine ligase |
| ABUW_0409 | -1.4053967 | 0.00014825 | *rplB* | ribosomal protein L2 |
| ABUW_1529 | -1.4057409 | 0.00041701 | *-* | ABC transporter, ATP-binding protein |
| ABUW_1523 | -1.4062759 | 0.00058754 | *-* | transporter, major facilitator family |
| ABUW_2205 | -1.4062877 | 2.9768E-05 | *hscB* | Fe-S protein assembly co-chaperone HscB |
| ABUW_3386 | -1.4070054 | 2.80E-07 | *nagZ* | N-acetyl-beta-glucosaminidase |
| ABUW_0346 | -1.4145431 | 2.05E-08 | *-* | hypothetical protein |
| ABUW_2961 | -1.4158313 | 3.937E-06 | *-* | peptidyl-prolyl cis-trans isomerase |
| ABUW_3314 | -1.418139 | 8.0934E-06 | *pntA1* | NAD(P) transhydrogenase subunit alpha |
| ABUW_0549 | -1.4241666 | 3.6239E-05 | *-* | hypothetical protein |
| ABUW_0311 | -1.4260894 | 4.81E-07 | *gmk* | guanylate kinase |
| ABUW_0603 | -1.4291161 | 3.9744E-06 | *-* | hypothetical protein |
| ABUW_3364 | -1.4298857 | 2.9714E-06 | *-* | RND efflux system, outer membrane lipoprotein, NodT family |
| ABUW_3701 | -1.431479 | 3.93E-07 | *-* | hypothetical protein |
| ABUW_1300 | -1.4331722 | 0.00740236 | *-* | hypothetical protein |
| ABUW_0713 | -1.4333038 | 7.36E-07 | *gatA* | glutamyl-tRNA(Gln) amidotransferase, A subunit |
| ABUW_0412 | -1.4339044 | 2.4075E-05 | *rpsC* | ribosomal protein S3 |
| ABUW_1009 | -1.4339405 | 3.35E-07 | *bcp* | bacterioferritin comigratory protein |
| ABUW_1220 | -1.435048 | 7.36E-07 | *-* | malate dehydrogenase |
| ABUW_3349 | -1.4355095 | 3.9531E-06 | *-* | hypothetical protein |
| ABUW_1770 | -1.4367907 | 7.3041E-06 | *fruA* | PTS system fructose-specific EIIBC component |
| ABUW_0523 | -1.4395437 | 9.96E-08 | *-* | hypothetical protein |
| ABUW_3185 | -1.443233 | 7.36E-07 | *nrdB* | ribonucleoside-diphosphate reductase, beta subunit |
| ABUW_0428 | -1.4437539 | 1.45E-05 | *rpsM* | ribosomal protein S13 |
| ABUW_0413 | -1.4448108 | 4.8223E-06 | *rplP* | ribosomal protein L16 |
| ABUW_1097 | -1.4450917 | 8.7748E-06 | *thiE* | thiamine-phosphate pyrophosphorylase |
| ABUW_2845 | -1.4455377 | 7.67E-09 | *-* | hypothetical protein |
| ABUW_0524 | -1.4496319 | 1.73E-08 | *-* | YCII-related protein |
| ABUW_2207 | -1.453892 | 4.6575E-05 | *fdx* | ferredoxin, 2Fe-2S type |
| ABUW_0043 | -1.4561364 | 0.00028449 | *-* | transcriptional regulator, LysR family |
| ABUW_0223 | -1.4603588 | 4.741E-05 | *-* | hypothetical protein |
| ABUW_0907 | -1.4618763 | 2.874E-06 | *folD* | methenyltetrahydrofolate cyclohydrolase |
| ABUW_0876 | -1.4637437 | 6.3557E-06 | *sucD* | succinyl-CoA synthetase, alpha subunit |
| ABUW_3678 | -1.4651386 | 9.40E-08 | *tniA* | transposase protein A |
| ABUW_0405 | -1.468387 | 0.00061721 | *rpsJ* | ribosomal protein S10 |
| ABUW_2634 | -1.4712514 | 6.14E-07 | *mutS* | DNA mismatch repair protein MutS |
| ABUW_3738 | -1.4717221 | 2.5902E-05 | *atpB* | ATP synthase F0, A subunit |
| ABUW_1098 | -1.4744492 | 7.62E-08 | *hemL* | glutamate-1-semialdehyde-2,1-aminomutase |
| ABUW_3350 | -1.4755559 | 2.4208E-05 | *prfC* | peptide chain release factor 3 |
| ABUW_0408 | -1.4764359 | 0.00022847 | *rplW* | ribosomal protein L23 |
| ABUW_3578 | -1.4776778 | 2.72E-08 | *-* | DSBA oxidoreductase |
| ABUW_2979 | -1.4779674 | 0.0068551 | *-* | hypothetical protein |
| ABUW_0926 | -1.4833946 | 3.0202E-06 | *-* | PP-loop domain protein |
| ABUW_0623 | -1.4846943 | 1.37E-08 | *accD* | acetyl-CoA carboxylase, carboxyl transferase, beta subunit |
| ABUW_1735 | -1.4866166 | 5.1372E-06 | *pyrH* | Uridylate kinase |
| ABUW_0039 | -1.4869573 | 6.19E-09 | *dapB* | dihydrodipicolinate reductase |
| ABUW_0162 | -1.4870988 | 2.26E-07 | *guaB* | inosine-5'-monophosphate dehydrogenase |
| ABUW_3392 | -1.4877989 | 6.77E-08 | *gntT* | high affinity gluconate permease |
| ABUW_0411 | -1.488076 | 1.5943E-05 | *rplV* | ribosomal protein L22 |
| ABUW_0598 | -1.497544 | 4.68E-07 | *aspS* | aspartyl-tRNA synthetase |
| ABUW_0334 | -1.4998035 | 1.4043E-05 | *-* | hypothetical protein |
| ABUW_3832 | -1.5033324 | 1.0246E-06 | *ptp* | protein-tyrosine-phosphatase ptp |
| ABUW_2731 | -1.5079712 | 1.22E-07 | *pyrX* | dihydroorotase |
| ABUW_1253 | -1.5117416 | 0.03051459 | *-* | hypothetical protein |
| ABUW_0278 | -1.5124187 | 8.44E-07 | *gltX* | glutamyl-tRNA synthetase |
| ABUW_3833 | -1.5131988 | 5.07E-07 | *ptk* | tyrosine-protein kinase ptk |
| ABUW_3670 | -1.5158792 | 1.01E-07 | *arsB* | arsenical-resistance protein |
| ABUW_2264 | -1.5179385 | 3.49E-09 | *gidB* | methyltransferase GidB |
| ABUW_2279 | -1.5202705 | 5.3405E-06 | *-* | nucleoprotein/polynucleotide-associated enzyme |
| ABUW_0042 | -1.5242848 | 2.50E-07 | *alrA* | aldo/keto reductase |
| ABUW_3465 | -1.5271513 | 7.36E-07 | *-* | transcriptional regulator, LysR family |
| ABUW_0980 | -1.5282151 | 1.6125E-05 | *purN* | phosphoribosylglycinamide formyltransferase |
| ABUW_2295 | -1.5325519 | 2.4148E-05 | *tkt* | transketolase |
| ABUW_3720 | -1.5370371 | 3.94E-07 | *-* | hypothetical protein |
| ABUW_1043 | -1.5375549 | 1.93E-07 | *aspC* | aspartate aminotransferase A |
| ABUW_2202 | -1.5390487 | 5.18E-08 | *iscS* | cysteine desulfurase IscS |
| ABUW_2917 | -1.5417173 | 8.68E-08 | *yhgI* | IscR-regulated protein YhgI |
| ABUW_1134 | -1.5419959 | 6.76E-10 | *-* | major facilitator superfamily MFS_1 |
| ABUW_2850 | -1.5427406 | 0.00025787 | *-* | lysine exporter protein ArgO |
| ABUW_2925 | -1.5431618 | 7.14E-08 | *pit* | phosphate transporter |
| ABUW_2289 | -1.5445071 | 2.06E-07 | *trmA* | tRNA (uracil-5-)-methyltransferase |
| ABUW_0410 | -1.5458946 | 2.2313E-05 | *rpsS* | ribosomal protein S19 |
| ABUW_0548 | -1.5468455 | 0.0088291 | *-* | hypothetical protein |
| ABUW_0993 | -1.5499833 | 2.70E-09 | *tolB* | Tol-Pal system beta propeller repeat protein TolB |
| ABUW_0351 | -1.5510033 | 3.08E-09 | *-* | bifunctional succinylornithine transaminase/acetylornithine transaminase |
| ABUW_2267 | -1.5531215 | 1.03E-08 | *ostA* | organic solvent tolerance protein |
| ABUW_1824 | -1.5570726 | 9.84E-07 | *-* | hypothetical protein |
| ABUW_2424 | -1.5627101 | 0.00170794 | *-* | ABC-type amino acid transport system |
| ABUW_2913 | -1.5627157 | 1.27E-08 | *-* | carbonate dehydratase |
| ABUW_3313 | -1.5653078 | 5.3727E-05 | *pntA2* | NAD(P) transhydrogenase subunit alpha |
| ABUW_1304 | -1.5742395 | 0.02280182 | *-* | hypothetical protein |
| ABUW_3359 | -1.5760854 | 2.23E-07 | *leuS* | leucyl-tRNA synthetase |
| ABUW_0874 | -1.581954 | 2.90E-07 | *lpdA1* | dihydrolipoamide dehydrogenase |
| ABUW_0918 | -1.5831007 | 1.6333E-05 | *groL* | chaperonin GroL |
| ABUW_0836 | -1.5882063 | 1.7996E-05 | *valS* | valyl-tRNA synthetase |
| ABUW_3022 | -1.5963163 | 5.08E-09 | *hemB* | delta-aminolevulinic acid dehydratase |
| ABUW_3677 | -1.5987205 | 1.91E-09 | *tniB* | transposition protein B |
| ABUW_0352 | -1.6020765 | 3.28E-11 | *astA1* | arginine N-succinyltransferase |
| ABUW_1575 | -1.6022208 | 2.76E-08 | *-* | FMN-binding protein |
| ABUW_0873 | -1.604376 | 1.0016E-06 | *sucB* | 2-oxoglutarate dehydrogenase, E2 component, dihydrolipoamide succinyltransferase |
| ABUW_1771 | -1.6054787 | 1.3679E-06 | *pfkB* | 1-phosphofructokinase |
| ABUW_3312 | -1.6063935 | 2.9306E-06 | *pntB* | NAD(P) transhydrogenase subunit beta |
| ABUW_3669 | -1.6103961 | 7.62E-08 | *arsC* | arsenate reductase |
| ABUW_2749 | -1.6107868 | 7.0454E-06 | *lysC* | aspartate kinase |
| ABUW_0007 | -1.6113286 | 3.37E-08 | *-* | ABC transporter, ATP-binding protein |
| ABUW_2486 | -1.611882 | 0.0007045 | *-* | transcriptional regulator, GntR family |
| ABUW_2975 | -1.6153043 | 0.00022997 | *-* | homocysteine S-methyltransferase family protein |
| ABUW_2732 | -1.6218044 | 1.06E-10 | *pyrB* | aspartate carbamoyltransferase |
| ABUW_1166 | -1.6247801 | 5.71E-07 | *-* | threonine ammonia-lyase |
| ABUW_2823 | -1.6247919 | 1.57E-07 | *argG* | argininosuccinate synthase |
| ABUW_1778 | -1.6266097 | 8.30E-08 | *map2* | methionine aminopeptidase, type I |
| ABUW_3851 | -1.6275686 | 6.0708E-05 | *-* | hypothetical protein |
| ABUW_2898 | -1.6312288 | 3.12E-09 | *-* | hypothetical protein |
| ABUW_0415 | -1.6380906 | 5.45E-07 | *rpsQ* | ribosomal protein S17 |
| ABUW_1746 | -1.6386679 | 1.23E-08 | *-* | hypothetical protein |
| ABUW_1564 | -1.6406862 | 4.63E-09 | *-* | hypothetical protein |
| ABUW_1533 | -1.6429398 | 7.9958E-05 | *fis* | transcriptional regulator, Fis-type |
| ABUW_0417 | -1.6467431 | 6.30E-07 | *rplX* | ribosomal protein L24 |
| ABUW_1127 | -1.6479607 | 2.09E-08 | *dacC* | penicillin-binding protein 6 (D-alanyl-D-alanine carboxypeptidase) |
| ABUW_3719 | -1.6503254 | 5.0451E-05 | *-* | TonB-dependent copper receptor |
| ABUW_0090 | -1.6562762 | 2.32E-08 | *glmU* | UDP-N-acetylglucosamine diphosphorylase/glucosamine-1- phosphate N-acetyltransferase |
| ABUW_1333 | -1.6611091 | 1.6673E-06 | *glyA* | glycine hydroxymethyltransferase |
| ABUW_0703 | -1.6625652 | 6.91E-08 | *-* | hypothetical protein |
| ABUW_1254 | -1.6761967 | 0.00516592 | *-* | hypothetical protein |
| ABUW_3305 | -1.6828307 | 4.10E-09 | *cysM* | cysteine synthase B |
| ABUW_2852 | -1.6839971 | 8.10E-07 | *-* | transcriptional regulator, MerR family |
| ABUW_0917 | -1.68695 | 4.29E-07 | *groS* | chaperonin GroS |
| ABUW_3393 | -1.6911649 | 1.64E-08 | *eda* | khg/kdpg aldolase |
| ABUW_0997 | -1.6947535 | 6.63E-08 | *ybgC* | tol-pal system-associated acyl-CoA thioesterase |
| ABUW_3065 | -1.7000284 | 6.99E-10 | *fabB* | 3-oxoacyl-[acyl-carrier-protein] synthase 1 |
| ABUW_0704 | -1.702364 | 3.94E-10 | *agcS* | amino acid transport protein |
| ABUW_0367 | -1.7070798 | 5.70E-08 | *glyQ* | glycyl-tRNA synthetase, alpha subunit |
| ABUW_2327 | -1.7099511 | 3.5282E-06 | *-* | hypothetical protein |
| ABUW_1050 | -1.7125847 | 1.5941E-06 | *gap* | glyceraldehyde-3-phosphate dehydrogenase |
| ABUW_3198 | -1.7146891 | 1.184E-05 | *-* | hypothetical protein |
| ABUW_0881 | -1.7254879 | 2.63E-10 | *trpS* | tryptophanyl-tRNA synthetase |
| ABUW_0354 | -1.7505056 | 5.55E-11 | *astB* | succinylarginine dihydrolase |
| ABUW_0875 | -1.75437 | 9.73E-10 | *sucC* | succinyl-CoA synthase, beta subunit |
| ABUW_0520 | -1.7590519 | 1.75E-09 | *-* | nucleotide-binding protein |
| ABUW_3671 | -1.7606394 | 6.32E-10 | *arsH* | arsenical resistance protein ArsH |
| ABUW_0414 | -1.76821 | 2.48E-07 | *rpmC* | ribosomal protein L29 |
| ABUW_3668 | -1.7685218 | 3.86E-07 | *arsR* | arsenical resistance operon repressor |
| ABUW_0014 | -1.7686834 | 1.74E-10 | *tyrS* | tyrosyl-tRNA synthetase |
| ABUW_0241 | -1.7742987 | 5.88E-11 | *-* | homoserine kinase |
| ABUW_1931 | -1.781723 | 1.3433E-05 | *-* | transporter, major facilitator family |
| ABUW_3471 | -1.7992047 | 1.2837E-06 | *-* | Hca operon transcriptional activator |
| ABUW_3838 | -1.8106694 | 4.99E-13 | *nadC* | nicotinate-nucleotide diphosphorylase |
| ABUW_1070 | -1.820353 | 8.86E-10 | *-* | D-ala-D-ala-carboxypeptidase penicillin-binding protein |
| ABUW_3588 | -1.8207192 | 1.81E-09 | *-* | lipoprotein, putative |
| ABUW_2423 | -1.8250959 | 0.00921125 | *-* | ArtM protein |
| ABUW_2342 | -1.8306837 | 5.48E-08 | *ychF* | GTP-binding protein YchF |
| ABUW_1131 | -1.8351959 | 9.71E-13 | *nlpD* | peptidase M23B |
| ABUW_0536 | -1.8407633 | 1.69E-13 | *queA* | S-adenosylmethionine:tRNA ribosyltransferase-isomerase |
| ABUW_3622 | -1.8584318 | 2.80E-07 | *-* | transcriptional regulator |
| ABUW_1141 | -1.8636728 | 6.51E-09 | *efp* | translation elongation factor P |
| ABUW_3631 | -1.8649292 | 1.14E-08 | *-* | hypothetical protein |
| ABUW_2727 | -1.8669868 | 3.35E-07 | *trmB* | tRNA (guanine-N(7)-)-methyltransferase |
| ABUW_1648 | -1.8880689 | 9.33E-12 | *-* | mechanosensitive ion channel family protein |
| ABUW_2602 | -1.888554 | 8.10E-07 | *-* | voltage-gated chloride channel |
| ABUW_3404 | -1.9288429 | 1.34E-12 | *piuC* | iron uptake factor |
| ABUW_1348 | -1.9318368 | 1.39E-13 | *-* | ferredoxin--NADP(+) reductase |
| ABUW_0353 | -1.9354253 | 7.08E-14 | *astD* | succinylglutamic semialdehyde dehydrogenase |
| ABUW_2853 | -1.9379244 | 1.30E-11 | *-* | hypothetical protein |
| ABUW_3796 | -1.9414737 | 3.2683E-06 | *-* | hypothetical protein |
| ABUW_2169 | -1.9440446 | 2.2086E-05 | *-* | putative membrane protein |
| ABUW_1249 | -1.9480319 | 1.61E-10 | *-* | integrase |
| ABUW_0142 | -1.9483011 | 3.82E-14 | *pyrE* | orotate phosphoribosyltransferase |
| ABUW_0607 | -1.9604174 | 1.08E-10 | *-* | hypothetical protein |
| ABUW_1531 | -1.962402 | 1.73E-08 | *purD* | phosphoribosylamine--glycine ligase |
| ABUW_2201 | -1.9692314 | 2.82E-11 | *iscR* | iscRSUA operon repressor |
| ABUW_0258 | -1.9741537 | 2.68E-08 | *-* | RNA binding S1 domain protein |
| ABUW_3852 | -2.0096827 | 1.74E-08 | *argA* | amino-acid N-acetyltransferase |
| ABUW_2179 | -2.0135601 | 4.22E-08 | *-* | hypothetical protein |
| ABUW_3797 | -2.0287252 | 0.00030427 | *-* | hypothetical protein |
| ABUW_0208 | -2.0332995 | 4.13E-12 | *-* | hypothetical protein |
| ABUW_0977 | -2.0383758 | 8.90E-12 | *-* | lipid A biosynthesis acyltransferase |
| ABUW_1772 | -2.074462 | 2.04E-13 | *fruB* | multiphosphoryl transfer protein |
| ABUW_3183 | -2.1026833 | 2.05E-10 | *nrdA* | ribonucleoside-diphosphate reductase alpha subunit |
| ABUW_2962 | -2.1234 | 2.63E-10 | *-* | YaeQ protein |
| ABUW_0872 | -2.1756375 | 4.42E-12 | *sucA* | 2-oxoglutarate dehydrogenase, E1 component |
| ABUW_3365 | -2.1849277 | 1.66E-13 | *fabI* | NADH-dependent enoyl-ACP reductase |
| ABUW_0990 | -2.1970137 | 8.92E-12 | *fbp1* | fructose-1,6-bisphosphatase |
| ABUW_0913 | -2.2016921 | 2.07E-07 | *pckG* | phosphoenolpyruvate carboxykinase |
| ABUW_2868 | -2.2166801 | 1.52E-14 | *-* | hypothetical protein |
| ABUW_3394 | -2.2234323 | 3.16E-15 | *edd* | phosphogluconate dehydratase |
| ABUW_0666 | -2.2326103 | 3.93E-15 | *-* | hypothetical protein |
| ABUW_0639 | -2.2331558 | 8.87E-14 | *-* | transcriptional regulator, AraC family |
| ABUW_3454 | -2.2573336 | 1.91E-17 | *-* | putative L-asparaginase I |
| ABUW_0030 | -2.2589131 | 2.13E-14 | *-* | uracil-xanthine permease |
| ABUW_2557 | -2.2901273 | 2.38E-11 | *-* | hypothetical protein |
| ABUW_3598 | -2.292788 | 9.87E-08 | *tuf2* | translation elongation factor Tu |
| ABUW_0233 | -2.3014883 | 5.20E-11 | *-* | hypothetical protein |
| ABUW_1532 | -2.3093017 | 1.07E-08 | *purH* | phosphoribosylaminoimidazolecarboxamide formyltransferase/IMP cyclohydrolase |
| ABUW_0356 | -2.3837181 | 2.29E-22 | *-* | hypothetical protein |
| ABUW_2982 | -2.3960321 | 2.60E-11 | *-* | TonB family protein |
| ABUW_1104 | -2.4156783 | 2.12E-12 | *-* | oxidoreductase |
| ABUW_1105 | -2.4490913 | 2.39E-18 | *-* | fatty acid desaturase |
| ABUW_1696 | -2.4640588 | 1.44E-11 | *accC* | acetyl-CoA carboxylase, biotin carboxylase |
| ABUW_3581 | -2.4767229 | 4.47E-15 | *htpG* | chaperone protein HtpG |
| ABUW_1697 | -2.6217611 | 6.24E-17 | *accB* | acetyl-CoA carboxylase, biotin carboxyl carrier protein |
| ABUW_0350 | -2.6283805 | 2.56E-18 | *gdh* | glutamate dehydrogenase |
| ABUW_2382 | -2.7882126 | 1.407E-05 | *tauA* | taurine ABC transporter, periplasmic binding protein |
| ABUW_3632 | -2.8664333 | 1.12E-17 | *feoB* | ferrous iron transport protein B |
| ABUW_2828 | -2.9753949 | 5.80E-24 | *-* | TonB-dependent siderophore receptor |
| ABUW_1563 | -2.9832934 | 3.60E-25 | *-* | hypothetical protein |
| ABUW_3875 | -3.0098493 | 2.49E-22 | *-* | lipoprotein, putative |
| ABUW_2980 | -3.061256 | 1.85E-12 | *-* | hypothetical protein |
| ABUW_3633 | -3.1880159 | 1.18E-21 | *feoA* | iron transporter |
| ABUW_3424 | -3.2099623 | 4.93E-16 | *-* | biopolymer transport protein ExbD/TolR |
| ABUW_2183 | -3.2671843 | 1.76E-22 | *-* | dimethylmenaquinone methyltransferase |
| ABUW_2180 | -3.3852727 | 1.30E-25 | *-* | hypothetical protein |
| ABUW_1167 | -3.434355 | 1.99E-09 | *-* | hypothetical protein |
| ABUW_1800 | -3.4369342 | 5.17E-28 | *-* | ferrichrome-iron receptor |
| ABUW_1976 | -3.447048 | 1.85E-27 | *adeC* | multidrug efflux protein AdeC |
| ABUW_0698 | -3.4957484 | 8.72E-31 | *-* | flavodoxin/nitric oxide synthase |
| ABUW_1646 | -3.5478686 | 9.54E-20 | *-* | TonB-dependent siderophore receptor |
| ABUW_2104 | -3.5597624 | 7.09E-15 | *aspA* | fumarate lyase |
| ABUW_2074 | -3.5902833 | 7.08E-13 | *-* | transcriptional regulator, fur family |
| ABUW_2181 | -3.7864402 | 1.30E-17 | *-* | hypothetical protein |
| ABUW_3464 | -3.8324501 | 4.37E-15 | *-* | hypothetical protein |
| ABUW_2983 | -3.8499375 | 6.55E-19 | *-* | hypothetical protein |
| ABUW_2184 | -3.8611947 | 3.84E-29 | *-* | hypothetical protein |
| ABUW_3425 | -3.8882997 | 1.04E-19 | *-* | MotA/TolQ/ExbB proton channel |
| ABUW_1722 | -4.0015898 | 2.91E-25 | *fumC* | fumarate hydratase, class II |
| ABUW_0307 | -4.059348 | 2.93E-14 | *-* | hypothetical protein |
| ABUW_1364 | -4.1316272 | 3.81E-15 | *-* | alpha/beta hydrolase fold protein |
| ABUW_1975 | -4.1568476 | 3.21E-36 | *adeB* | multidrug efflux protein AdeB |
| ABUW_3426 | -4.1797956 | 1.83E-27 | *-* | TonB protein |
| ABUW_2168 | -4.3757216 | 1.06E-24 | *-* | hypothetical protein |
| ABUW_1598 | -4.7264328 | 4.42E-18 | *-* | hypothetical protein |
| ABUW_1974 | -4.7305083 | 1.08E-45 | *adeA* | multidrug efflux protein AdeA |
| ABUW_1187 | -4.7680562 | 1.33E-24 | *basI* | phosphopantetheinyl transferase |
| ABUW_2182 | -4.8770607 | 1.03E-29 | *-* | TonB-dependent receptor |
| ABUW_1655 | -4.8897704 | 7.80E-29 | *-* | TonB-dependent siderophore receptor |
| ABUW_2981 | -5.0048213 | 1.07E-25 | *-* | heme oxygenase |
| ABUW_2985 | -5.0121842 | 7.47E-27 | *-* | hypothetical protein |
| ABUW_2185 | -5.1903337 | 1.11E-29 | *-* | siderophore biosynthesis protein |
| ABUW_0159 | -5.2189277 | 5.55E-35 | *-* | TonB-dependent receptor protein |
| ABUW_2186 | -5.2478511 | 3.14E-37 | *-* | siderophore biosynthesis protein |
| ABUW_1186 | -5.4005017 | 2.39E-20 | *basH* | thioesterase |
| ABUW_2167 | -5.4366336 | 1.39E-36 | *-* | iron-regulated membrane protein |
| ABUW_2166 | -5.5194361 | 1.81E-30 | *-* | hypothetical protein |
| ABUW_1177 | -5.5533783 | 6.33E-19 | *bauA* | ferric acinetobactin receptor |
| ABUW_0143 | -5.7791884 | 3.19E-18 | *-* | TonB-dependent receptor protein |
| ABUW_2987 | -5.7835154 | 8.01E-21 | *-* | RNA polymerase sigma factor FecI |
| ABUW_1355 | -5.8219782 | 1.17E-29 | *-* | complement control module protein |
| ABUW_3403 | -5.9045355 | 1.68E-33 | *-* | TonB-dependent receptor |
| ABUW_2075 | -5.9415855 | 4.31E-21 | *-* | isochorismatase |
| ABUW_2916 | -5.9922207 | 2.11E-17 | *pfeA* | TonB dependent outer membrane siderophore receptor protein |
| ABUW_1176 | -5.9992594 | 6.27E-21 | *bauB* | ferric acinetobactin transport system periplasmic binding protein |
| ABUW_2165 | -6.0606158 | 1.34E-29 | *-* | outer-membrane receptor for Fe(III)-coprogen,Fe(III)-ferrioxamine B and Fe(III)-rhodotrulic acid |
| ABUW_2986 | -6.0781585 | 2.92E-16 | *-* | putative transmembrane sensor protein FecR |
| ABUW_1185 | -6.1919625 | 3.23E-23 | *-* | ABC transporter |
| ABUW_1169 | -6.2564954 | 4.90E-21 | *basA* | non-ribosomal peptide synthetase |
| ABUW_1183 | -6.3451659 | 4.46E-24 | *-* | hypothetical protein |
| ABUW_1174 | -6.407212 | 6.30E-17 | *bauC* | ferric acinetobactin transport system permease |
| ABUW_1175 | -6.4897324 | 7.73E-20 | *bauE* | ferric acinetobactin transport system ATP-binding protein |
| ABUW_1178 | -6.5574047 | 3.14E-37 | *basC* | nonribosomal peptide synthetase BasC |
| ABUW_2187 | -6.614078 | 1.08E-45 | *-* | transporter, major facilitator family |
| ABUW_2188 | -6.6884501 | 2.95E-34 | *-* | hypothetical protein |
| ABUW_1182 | -6.6929468 | 8.36E-26 | *basG* | histidine decarboxylase |
| ABUW_2076 | -6.7825582 | 7.66E-21 | *-* | 2,3-dihydro-2,3-dihydroxybenzoate dehydrogenase |
| ABUW_1188 | -6.8604867 | 7.96E-22 | *basJ* | isochorismate synthetase |
| ABUW_2189 | -6.9424781 | 3.09E-32 | *-* | IucA/IucC-family aerobactin siderophore biosynthesis component |
| ABUW_1168 | -7.002075 | 3.82E-19 | *bauF* | siderophore-interacting protein |
| ABUW_1181 | -7.0152899 | 1.91E-20 | *basF* | 2,3 dihydro-2,3 dihydroxybenzoate synthase |
| ABUW_1173 | -7.0192228 | 1.76E-22 | *bauD* | ferric acinetobactin transport system permease |
| ABUW_1184 | -7.0493817 | 3.84E-29 | *-* | ABC transporter, ATP-binding protein |
| ABUW_1170 | -7.4440638 | 1.64E-20 | *basB* | non-ribosomal peptide synthetase |
| ABUW_2178 | -7.466404 | 2.67E-25 | *-* | rhizobactin siderophore biosynthesis protein RhbD |
| ABUW_1179 | -7.5410319 | 1.07E-25 | *basD* | nonribosomal peptide synthetase BasD |
| ABUW_1172 | -7.5742538 | 4.73E-13 | *-* | hypothetical protein |
| ABUW_1180 | -7.9274727 | 9.46E-21 | *basE* | 2,3-dihydroxybenzoate-AMP ligase |
| ABUW_1171 | -8.2359059 | 2.34E-12 | *-* | hypothetical protein |

**Table S2.** The overview of metabolic fluxes of AB5075 during infection at 2 and 4 h.

| **Reaction ID** | **Subsystem** | **Flux (2h)** | **Flux (4h)** | **FDR** |
| --- | --- | --- | --- | --- |
| R_VALtex |  | 0 | -6.935287 | 0 |
| R_CO2tex |  | 0 | -3.3713407 | 0 |
| R_NH4tex |  | 0.00175978 | -2.8108668 | 0.00275616 |
| R_NH4tex |  | 0.00175978 | -2.8108668 | 0.00275616 |
| R_NH4tpp |  | 0 | -2.8108668 | 0 |
| R_HCO3E |  | 0.618 | 0.95304308 | 0.05513022 |
| R_PIabc |  | 0.21727503 | 0 | 0 |
| R_HSERTA |  | 0.10016802 | 0 | 0 |
| R_FE3tex |  | 0 | -0.0045758 | 0 |
| R_SO4t2 |  | 0.05648642 | 0 | 0 |
| R_CYTBO3_4pp |  | 12.9114386 | 0 | 0 |
| R_3OXCOAT |  | 0 | 0.0221279 | 0 |
| R_COBALT2tex |  | 2.25E-05 | 0 | 0 |
| R_AACPS3 |  | 0 | 0.03901374 | 0 |
| R_AACPS4 |  | 0 | 0.04598332 | 0 |
| R_AACPS7 |  | 0 | 0.02378949 | 0 |
| R_3HAD100 | Fatty acid biosynthesis | 0 | 0.08020871 | 0 |
| R_3HAD40 | Fatty acid biosynthesis | 0 | 0.08020871 | 0 |
| R_THRAi | Glycine, serine and threonine metabolism | 0.54718776 | 0 | 0 |
| R_GHMT2r | Glycine, serine and threonine metabolism | 0 | -0.381519 | 0 |
| R_AKGDH | Citrate cycle | 4.3296128 | 0 | 0 |
| R_DHAD1 | Valine, leucine and isoleucine biosynthesis | 0.1970439 | 0 | 0 |
| R_ACOAD20 |  | 0 | 0.09212331 | 0 |
| R_3HAD60 | Fatty acid biosynthesis | 0 | 0.08020871 | 0 |
| R_ACOAD7f |  | 0 | -0.0390137 | 0 |
| R_METS | Cysteine and methionine metabolism | 0.10029108 | 0 | 0 |
| R_ACPPAT160 |  | 0 | 0.01950687 | 0 |
| R_ACPPAT161 |  | 0 | 0.02299166 | 0 |
| R_AHSERL2 | Cysteine and methionine metabolism | 0.10016802 | 0 | 0 |
| R_FUM | Citrate cycle | 5.25478164 | 2.14784973 | 0 |
| R_OAADC | Pentose phospate pathway | 0.86573947 | 0 | 0 |
| R_ALDD2y | Glycolysis | 0 | 0.54718776 | 0 |
| R_PPCK | Glycolysis | 0.50251102 | 0 | 0 |
| R_PRPPS | Pentose phospate pathway | 0.23080152 | 0 | 0 |
| R_ADK3 | Purine metabolism | 0 | -0.1368009 | 0 |
| R_AGPAT160 | Glycerolipid metabolism | 0.01438248 | 0.01950687 | 0.05725796 |
| R_AGPAT161 | Glycerolipid metabolism | 0.01695183 | 0.02299166 | 0.00423664 |
| R_HSERTA | Cysteine and methionine metabolism | 0.10016802 | 0 | 0 |
| R_MTHFR3 | One carbon pool by folate | 0.10029108 | 0.0001669 | 0.00052366 |
| R_GARFT | Purine metabolism | 0.10091404 | 0.2368009 | 0 |
| R_HXAND |  | 0.05481905 | 0 | 0 |
| R_HXAND |  | 0.05481905 | 0 | 0 |
| R_MDH2 | Pyruvate metabolism | 8.50680246 | 0 | 0 |
| R_NDPK1 | Purine metabolism | 0.10086378 | 0.2368009 | 0 |
| R_CU2tex |  | 0.0001598 | 0.00021673 | 0.00110072 |
| R_MNtex |  | 0 | 0.00021123 | 0 |
| R_ALATA_L |  | 0 | 1.01026362 | 0 |
| R_GLUt2r |  | 4.05587298 | 0 | 0 |
| R_SUCDi |  | 4.33010368 | 0.55316194 | 0.00065846 |
| R_ENO | Glycolysis | -0.4249176 | -0.8496099 | 0 |
| R_AMMQLT8 |  | 0 | 3.06E-05 | 0 |
| R_APRAUR | Riboflavin metabolism | 0.00010052 | 0.00013634 | 0.00040996 |
| R_ACOTA | Arginine and proline metabolism | 0 | -0.0904187 | 0 |
| R_AGPR | Arginine and proline metabolism | 0 | -0.0904187 | 0 |
| R_DHDPS | Lysine biosynthesis | 0.08362751 | 0 | 0 |
| R_HSDx | Glycine, serine and threonine metabolism | 0 | -0.0775479 | 0 |
| R_KARA1 | Valine, leucine and isoleucine biosynthesis | -0.1970439 | -0.2672494 | 0.05409959 |
| R_GAPD | Glycolysis | -0.4249176 | -0.8496099 | 0 |
| R_ACHBS | Valine, leucine and isoleucine biosynthesis | 0.06547981 | 0 | 0 |
| R_ACHBS | Valine, leucine and isoleucine biosynthesis | 0.06547981 | 0 | 0 |
| R_AHCYSNS | Cysteine and methionine metabolism | 0.05471853 | 0 | 0 |
| R_ASPTA |  | 0 | -0.9238789 | 0 |
| R_ALCD2x | Methane metabolism | -0.9129891 | -2.443142 | 0.00177481 |
| R_ALCD2y |  | 0.91298909 | 0 | 0 |
| R_SUCOAACTr | Pyruvate metabolism | 0.67694632 | 0 | 0 |
| R_CPK1 | Purine metabolism | 0.04028466 | -0.0465553 | 0 |
| R_UMPK | Pyrimidine metabolism | 0.08520293 | 0 | 0.00023 |
| R_CDPMEK |  | 0.00018031 | 0.00024455 | 0.00061337 |
| R_IPPMIa | Valine, leucine and isoleucine biosynthesis | -0.1015413 | -0.1377198 | 0.00067337 |
| R_CLtex |  | 0.00117311 | 0.00159108 | 0.00041337 |
| R_MCTP1App | Peptidoglycan biosynthesis | 0.00313145 | 0.00824716 | 2.40E-05 |
| R_COabc |  | 0 | 3.06E-05 | 0 |
| R_IPPMIa | Valine, leucine and isoleucine biosynthesis | -0.1015413 | -0.1377198 | 0.0020917 |
| R_CPPPGO | Porphyrin and chlorophyll metabolism | 0 | 6.82E-05 | 0 |
| R_CTECOAI7 |  | 0 | 0.03901374 | 0 |
| R_TKT2 | Pentose phospate pathway | 0 | -0.0837762 | 0 |
| R_LPADSS | Lipopolysaccharide biosynthesis | 0.00338502 | 0.00694737 | 0.0009834 |
| R_IPPMIb | Valine, leucine and isoleucine biosynthesis | -0.1015413 | -0.1377198 | 0.0020917 |
| R_IPPMIb | Valine, leucine and isoleucine biosynthesis | -0.1015413 | -0.1377198 | 0.0020917 |
| R_NDPK3 | Purine metabolism | 0.03256453 | 0.08069914 | 0 |
| R_PTAr |  | 0 | -0.2849512 | 0 |
| R_FDH |  | 0.25708661 | 0 | 0 |
| R_FDH7 |  | 0.19105394 | 0 | 0 |
| R_RNTR3c |  | 0.01198646 | 0 | 0 |
| R_SADT | Purine metabolism | 0.03550872 | 0.07350872 | 0 |
| R_DALAt2pp |  | 0.00313145 | 0.00424716 | 0.00056889 |
| R_VALTA | Valine, leucine and isoleucine degradation | -0.0953728 | -0.1293535 | 0.00056889 |
| R_DHQTi | Phenylalanine, tyrosine and tryptophan biosynthesis | 0.03128022 | 0 | 0 |
| R_DASYN160 | Glycerophospholipid metabolism | 0.01438248 | 0.01950687 | 0.00056889 |
| R_DASYN161 | Glycerophospholipid metabolism | 0.01695183 | 0.02299166 | 2.40E-05 |
| R_DB4PS | Riboflavin metabolism | 0.00020104 | 0.00027267 | 2.40E-05 |
| R_PPND | Phenylalanine, tyrosine and tryptophan biosynthesis | 0.03112944 | 0 | 0 |
| R_CHORS | Phenylalanine, tyrosine and tryptophan biosynthesis | 0.03128022 | 0.0002045 | 0.00056889 |
| R_DDPA | Phenylalanine, tyrosine and tryptophan biosynthesis | 0.03128022 | 0.0002045 | 2.40E-05 |
| R_DHQS | Phenylalanine, tyrosine and tryptophan biosynthesis | 0.03128022 | 0.0002045 | 2.40E-05 |
| R_PSCVT | Phenylalanine, tyrosine and tryptophan biosynthesis | 0.03128022 | 0.0002045 | 2.40E-05 |
| R_SHK3Dr | Phenylalanine, tyrosine and tryptophan biosynthesis | 0.03128022 | 0.0002045 | 2.40E-05 |
| R_DHFR | One carbon pool by folate | 0.00604811 | 0.00820301 | 2.40E-05 |
| R_DHFS | Folate biosynthesis | 0.00015078 | 0.0002045 | 2.40E-05 |
| R_SADT | Purine metabolism | 0.03550872 | 0.07350872 | 0 |
| R_NTD11 | Purine metabolism | 0.05481905 | 0.05550872 | 0 |
| R_DHPACCOAHIT |  | 0 | 0.0221279 | 0 |
| R_DHPACCOAHIT |  | 0 | 0.0221279 | 0 |
| R_DHPACCOAHIT |  | 0 | 0.0221279 | 0 |
| R_DHPPDA | Riboflavin metabolism | 0.00010052 | 0.00013634 | 2.40E-05 |
| R_DHPS | Folate biosynthesis | 0.00015078 | 0.0002045 | 2.40E-05 |
| R_SHKK | Phenylalanine, tyrosine and tryptophan biosynthesis | 0.03128022 | 0.0002045 | 2.40E-05 |
| R_SERAT | Cysteine and methionine metabolism | 0.0208205 | 0 | 0 |
| R_DMATT |  | 0.0000225 | 3.06E-05 | 0.0001963 |
| R_DMPPS |  | 0.0000225 | 3.06E-05 | 0.0001963 |
| R_DNMPPA |  | 0.00015078 | 0.0002045 | 0.00030031 |
| R_DNTPPA |  | 0 | 0.0002045 | 0 |
| R_DPCOAK | Pantothenate and CoA biosynthesis | 0.00012982 | 0.00017607 | 0.00030031 |
| R_XPPT | Purine metabolism | 0.05481905 | 0.05550872 | 0 |
| R_DXPRIi |  | 0.00018031 | 0.00024455 | 0.00030031 |
| R_DXPS |  | 0.00028083 | 0.00031271 | 0.00030031 |
| R_TPI | Glycolysis | -0.1869564 | -0.2393579 | 0 |
| R_3HAD80 | Fatty acid biosynthesis | 0 | 0.08020871 | 0 |
| R_ECOAH9ir |  | 0 | 0.18424661 | 0 |
| R_FBA | Glycolysis | -0.1551087 | -0.3261631 | 0 |
| R_FACOAE120 |  | 0 | 0.02378949 | 0 |
| R_FACOAE160 |  | 0 | 0.03901374 | 0 |
| R_FACOAL140 |  | 0 | 0.02378949 | 0 |
| R_FACOAL161 |  | 0 | 0.03901374 | 0 |
| R_IMPC | Purine metabolism | -0.1008638 | -0.1368009 | 0 |
| R_RPE | Pentose phospate pathway | -0.1338129 | -0.3872797 | 0 |
| R_FCLT | Porphyrin and chlorophyll metabolism | 0.0000503 | 6.82E-05 |  |
| R_G3PD2 | Glycerophospholipid metabolism | -0.0313343 | -0.0424985 |  |
| R_MOAT | Lipopolysaccharide biosynthesis | 0.00338502 | 0.00694737 | 0.0009834 |
| R_RNTR2c |  | 0.00608913 | 0 | 0 |
| R_FE3t |  | 0 | 0.00457577 | 0 |
| R_MOAT2 | Lipopolysaccharide biosynthesis | 0.00338502 | 0.00694737 | 0.0009834 |
| R_FEROpp |  | 0.00043994 | 0.00114394 | 0.00171218 |
| R_GLYCLTt2r |  | -0.0001508 | -0.0002045 | 0.00117138 |
| R_FMNAT | Riboflavin metabolism | 0.0000503 | 6.82E-05 | 0.00014903 |
| R_FMNRx |  | 0 | 0.27305647 | 0 |
| R_3HOPCD |  | -0.0000225 | -3.06E-05 | 0.00014903 |
| R_G1PACT |  | 0.02129583 | 0.0288834 | 0.343232 |
| R_G1SAT | Porphyrin and chlorophyll metabolism | 0.00080416 | 0.00109068 | 0.00223853 |
| R_TALA | Pentose phospate pathway | -0.0512412 | -0.1235035 | 0 |
| R_TKT1 | Pentose phospate pathway | -0.0512412 | -0.1235035 | 0 |
| R_RPI | Pentose phospate pathway | -0.1248418 | -0.2651122 | 0 |
| R_NADH8 |  | 0 | 3.06E-05 | 0 |
| R_TDSK | Lipopolysaccharide biosynthesis | 0.00338502 | 0.00694737 | 0.0009834 |
| R_DLYSPYRAT | Cysteine and methionine metabolism | 0.00939434 | 0 | 0 |
| R_5DOAN | Cysteine and methionine metabolism | 0.00010052 | 0 | 0 |
| R_DHORTS | Pyrimidine metabolism | -0.074555 | -0.1011185 | 0 |
| R_ORPT | Pyrimidine metabolism | -0.074555 | -0.1011185 | 0 |
| R_AMPMS2 | Thiamine metabolism | 5.03E-05 | 0 | 0 |
| R_GLUTRR | Porphyrin and chlorophyll metabolism | 0.00080416 | 0.00109068 | 0.00093965 |
| R_GLUTRS |  | 0.00080416 | 0.00109068 | 0.00093965 |
| R_GLYCL |  | 0.30819656 | 0.38992866 | 0.067432 |
| R_FE3Ri |  | 0 | 0.0010945 | 0 |
| R_DCTPD | Pyrimidine metabolism | 0.00589733 | 0.01299851 | 0 |
| R_GRTT |  | 0.0000225 | 3.06E-05 | 0.00292108 |
| R_GTPCI | Folate biosynthesis | 0.00015078 | 0.0002045 | 0.0036588 |
| R_GTPCII | Riboflavin metabolism | 0.00010052 | 0.00013634 | 0.0792108 |
| R_HACD6i |  | 0 | 0.02378949 | 0 |
| R_HADPCOADH3 |  | 0 | 0.0221279 | 0 |
| R_THZPSN | Thiamine metabolism | 5.03E-05 | 0 | 0 |
| R_HISt2r |  | 0.02135218 | 0.02895982 | 0.0727258 |
| R_HMBS | Porphyrin and chlorophyll metabolism | 0.00010052 | 0.00013634 | 0.06257957 |
| R_METAT | Cysteine and methionine metabolism | 0.00027384 | 0.00023507 | 0.07957426 |
| R_OHPBAT | Glycine, serine and threonine metabolism | 0.0000503 | 6.82E-05 | 0.07432 |
| R_URIDK2r | Pyrimidine metabolism | -0.0058973 | -0.0079985 | 0.17432 |
| R_ADNK1 | Purine metabolism | 0.00022358 | 0 | 0 |
| R_IMPD |  | 0 | 0.07435073 | 0 |
| R_IPDPS |  | 0.00015777 | 0.00021398 | 0.00132072 |
| R_PMPK | Thiamine metabolism | 0.0000503 | 6.82E-05 | 0.10387882 |
| R_TMPK | Thiamine metabolism | 0.0000503 | 6.82E-05 | 0.10387882 |
| R_TMPPP | Thiamine metabolism | 0.0000503 | 6.82E-05 | 0.10387882 |
| R_ASP1DC | beta-Alanine metabolism | 0.00012982 | 0.00017607 | 0.321 |
| R_PANTS | beta-Alanine metabolism | 0.00012982 | 0.00017607 | 0.321 |
| R_HETZK | Thiamine metabolism | 0 | 6.82E-05 | 0 |
| R_K2L4Aabcpp |  | 0.00438502 | 0.00594737 | 0.218867 |
| R_HMPK1 | Thiamine metabolism | 0 | 6.82E-05 | 0 |
| R_TMN | Thiamine metabolism | 0 | 6.82E-05 | 0 |
| R_3OAR60 | Fatty acid biosynthesis | 0 | 0.08020871 | 0 |
| R_3OAS100 | Fatty acid biosynthesis | 0 | 0.08020871 | 0 |
| R_KAS7 |  | 0 | 0.08499707 | 0 |
| R_3OAS120 | Fatty acid biosynthesis | 0 | 0.08020871 | 0 |
| R_KAS7 |  | 0 | 0.08499707 | 0 |
| R_PAPPT3 | Peptidoglycan biosynthesis | 0.0032629 | 0.00849433 | 2.78E-06 |
| R_KDOPP | Lipopolysaccharide biosynthesis | 0.00877004 | 0.02189475 | 0 |
| R_ADCL | Folate biosynthesis | 0.00015078 | 0.0002045 | 3.78E-04 |
| R_APSR |  | 0.05550872 | 0 | 0 |
| R_ASPO5 | Alanine, aspartate and glutamate metabolism | 0.00051342 | 0.00069635 | 7.62E-02 |
| R_UAGCVT | Amino sugar and nucleotide sugar metabolism | 0.0032629 | 0.00849433 | 3.78E-04 |
| R_DHORD5 | Pyrimidine metabolism | -0.0058973 | 0 | 0 |
| R_FLDR2 |  | 0 | 0.00825865 | 0 |
| R_MCOATA |  | 0.54392086 | 0.02378949 | 3.78E-04 |
| R_UAGPT3 | Peptidoglycan biosynthesis | 0.0032629 | 0.00849433 | 2.78E-06 |
| R_RNTR2c2 |  | 0 | 0.00825865 | 0 |
| R_GLCOAS | Citrate cycle | 0.0000225 | 3.06E-05 | 3.20E-03 |
| R_MECDPDH |  | 0 | 0.00024455 | 0 |
| R_MECDPS |  | 0.00018031 | 0.00024455 | 0.00105574 |
| R_MEPCT |  | 0.00018031 | 0.00024455 | 0.00105574 |
| R_PABB | Folate biosynthesis | 0.00015078 | 0.0002045 | 0.00105574 |
| R_ALAALAr | D-Alanine metabolism | 0.0032629 | 0.00849433 | 0.00240829 |
| R_UAAGDS | Lysine biosynthesis | 0.0032629 | 0.00849433 | 0.00240829 |
| R_METSOXR2 |  | 0 | 6.82E-05 | 0 |
| R_UAMAGS | D-Glutamine and D-glutamate metabolism | 0.0032629 | 0.00849433 |  |
| R_E4PD | Glycolysis | 0.0000503 | 6.82E-05 | 0.00E+00 |
| R_GCALDD | Glycolysis | 0.00015078 | 0.0002045 | 0 |
| R_MNabc_1 |  | 0 | 0.00021123 | 0 |
| R_UAMAS | D-Glutamine and D-glutamate metabolism | 0.0032629 | 0.00849433 | 0.00240829 |
| R_UAPGR | Amino sugar and nucleotide sugar metabolism | 0.0032629 | 0.00849433 | 0.00240829 |
| R_UGMDDS | Lysine biosynthesis | 0.0032629 | 0.00849433 | 0.00240829 |
| R_MOHMT | Pantothenate and CoA biosynthesis | 0.00012982 | 0.00017607 | 0.1320089 |
| R_MPTG | Peptidoglycan biosynthesis | 0.00313145 | 0.00824716 | 2.78E-06 |
| R_METS_1 | Cysteine and methionine metabolism | 0 | 0.0001669 | 0 |
| R_DTMPK | Pyrimidine metabolism | 0.00589733 | 0.01299851 | 0 |
| R_NDPK4 | Purine metabolism | 0.00589733 | 0.01299851 | 0 |
| R_NDPK8 | Purine metabolism | 0.00589733 | 0.01299851 | 0 |
| R_NADS1 | Nicotinate and nicotinamide metabolism | 0 | 0.00069635 | 0 |
| R_TMDS | Pyrimidine metabolism | 0.00589733 | 0.01299851 | 0 |
| R_NDPK6 | Purine metabolism | -0.0058973 | 0 | 0.00012 |
| R_CTPS2 | Pyrimidine metabolism | 0.04207666 | 0.04906981 | 0 |
| R_NDPK7 | Purine metabolism | 0 | 0.00825865 | 0 |
| R_SUCD1 | Citrate cycle | 0 | 0.01631875 | 0 |
| R_NNATr |  | 0.00051342 | 0.00069635 | 0.00049252 |
| R_NNDPR | Nicotinate and nicotinamide metabolism | 0.00051342 | 0.00069635 | 0.00049252 |
| R_NPHS |  | 0.0000225 | 3.06E-05 | 0.00267531 |
| R_OCBT |  | 0 | 0.09041866 | 0 |
| R_OCTDPS |  | 0.0000225 | 3.06E-05 | 0.00226231 |
| R_3OAS60 | Fatty acid biosynthesis | 0 | 0.08020871 | 0 |
| R_PSSA160 | Glycine, serine and threonine metabolism | 0.01438248 | 0.01950687 | 0.00226231 |
| R_MCITS | Propanoate metabolism | 0 | 0.18424661 | 0 |
| R_SUCD1 | Citrate cycle | 0 | 0.01631875 | 0 |
| R_SUCD1 | Citrate cycle | 0 | 0.01631875 | 0 |
| R_ORNTAC |  | 0 | 0.09041866 | 0 |
| R_ADSS | Purine metabolism | 0.04604473 | 0.06245017 | 0 |
| R_OXPTNDH |  | 0.0000225 | 3.06E-05 | 0.00027257 |
| R_DHQTi | Phenylalanine, tyrosine and tryptophan biosynthesis | 0 | 0.0002045 | 0 |
| R_PSSA161 | Glycine, serine and threonine metabolism | 0.01695183 | 0.02299166 | 0.00056889 |
| R_A5PISO |  | 0.00877004 | 0.01189475 | 0.00270985 |
| R_ADSL1r | Purine metabolism | 0.046 | 0.06245017 | 0 |
| R_ALDD1 |  | 0.06547981 | 0.27305647 | 0.00016575 |
| R_PDX5POi | Vitamin B6 metabolism | 0.0000503 | 6.82E-05 | 0.00208462 |
| R_PE160abcpp |  | 0 | 0.01404492 | 0 |
| R_PE161abcpp |  | 0.00474653 | 0.00643769 | 0.00246442 |
| R_PERD | Vitamin B6 metabolism | 0.0000503 | 6.82E-05 | 0.00033928 |
| R_GK1 | Purine metabolism | 0.05481905 | 0.07435073 | 0.0021 |
| R_PGAMT | Amino sugar and nucleotide sugar metabolism | -0.0212958 | -0.0288834 | 0.00223853 |
| R_PGM |  | 0.42491764 | 0.51960989 | 0.00117138 |
| R_PHEt2r |  | 0.04175527 | 0.07876031 | 0.00112426 |
| R_PHETA1 |  | 0 | 0.0221279 | 0 |
| R_KDOCT2 | Lipopolysaccharide biosynthesis | 0.00877004 | 0.02189475 | 0 |
| R_PROt2r |  | 0.04982167 | 0 | 0 |
| R_GLUR | D-Glutamine and D-glutamate metabolism | -0.0156572 | -0.0084943 | 0.00270985 |
| R_PNTK | Pantothenate and CoA biosynthesis | 0.00012982 | 0.00017607 | 0.0002032 |
| R_PPA |  | 0.63440921 | 0.89903374 | 0.00291041 |
| R_PPBNGS | Porphyrin and chlorophyll metabolism | 0.00040208 | 0.00054534 | 0.00061337 |
| R_PPCDC | Pantothenate and CoA biosynthesis | 0.00012982 | 0.00017607 | 0.00240829 |
| R_PPNCL2 | Pantothenate and CoA biosynthesis | 0 | 0.00017607 | 0 |
| R_PPPGO |  | 0.0000503 | 6.82E-05 | 0.24082884 |
| R_OIVD1r | Glycolysis | 0 | 0.02215847 | 0 |
| R_NDPK2 | Purine metabolism | 0.05585085 | 0.12200339 | 0 |
| R_ASPCT | Pyrimidine metabolism | 0.0746 | 0.1011185 | 0 |
| R_GMPS |  | 0.05481905 | 0.07435073 |  |
| R_OMPDC | Pyrimidine metabolism | 0.07455501 | 0.1011185 | 0 |
| R_PROt4 |  | 0 | 0.06757281 | 0 |
| R_DHORD2 | Pyrimidine metabolism | 0.07453248 | 0.1011185 | 0 |
| R_ASPK | Glycine, serine and threonine metabolism | 0.18379554 | 0.19097134 |  |
| R_PSD160 | Glycerophospholipid metabolism | 0.01438248 | 0.01950687 | 0.00153134 |
| R_PSD161 | Glycerophospholipid metabolism | 0.01695183 | 0.02299166 | 0.00142154 |
| R_GF6PTA | Alanine, aspartate and glutamate metabolism | 0.02129583 | 0.0288834 | 0.00240829 |
| R_DLYSOXGAT | Cysteine and methionine metabolism | -0.0093943 | 0 | 0 |
| R_ALAR | D-Alanine metabolism | 0 | 0.01274149 | 0 |
| R_PTPATi | Pantothenate and CoA biosynthesis | 0.00012982 | 0.00017607 | 0.00291041 |
| R_QULNS | Nicotinate and nicotinamide metabolism | 0.00051342 | 0.00069635 | 0.0010069 |
| R_RBFK | Riboflavin metabolism | 0.0000503 | 6.82E-05 | 0.00283456 |
| R_RBFSa | Riboflavin metabolism | 0.00020104 | 0.00027267 | 0.00283456 |
| R_RBFSb | Riboflavin metabolism | 0.00010052 | 0.00013634 | 0.00052366 |
| R_RNDR1 |  | 0.00589733 | 0.00799851 | 0.00149236 |
| R_RNDR3 |  | 0 | 0.00825865 | 0 |
| R_RNDR4 |  | 0 | 0.00799851 | 0.00149236 |
| R_DHAD3 | Valine, leucine and isoleucine biosynthesis | 0.06547981 | 0.08880985 | 0.00283456 |
| R_NADK | Nicotinate and nicotinamide metabolism | 0.00010075 | 0.00013664 | 0.0004242 |
| R_ADNK1 |  | 0 | 0.0001669 | 0 |
| R_DAPDC | Lysine biosynthesis | 0.07736462 | 0.10492914 | 0.0001963 |
| R_SDPTA |  | -0.0836275 | -0.1134235 | 0.0027258 |
| R_DAPE | Lysine biosynthesis | 0.08362751 | 0.11342347 | 0.0027258 |
| R_SHCHD2 | Porphyrin and chlorophyll metabolism | 0.0000503 | 6.82E-05 | 0.00052366 |
| R_SHCHF | Porphyrin and chlorophyll metabolism | 0.0000503 | 6.82E-05 | 0.00052366 |
| R_DHDPRy | Lysine biosynthesis | 0.08362751 | 0.11342347 | 0.00299939 |
| R_SDPDS | Lysine biosynthesis | 0.08362751 | 0.11342347 | 0.00299939 |
| R_FDMO2 |  | 0 | 0.27305647 | 0 |
| R_AICART | Purine metabolism | 0.101 | 0.2368009 | 0.00021 |
| R_GLUPRT | Purine metabolism | 0.10091404 | 0.2368009 | 0.0000082 |
| R_PRAGSr | Purine metabolism | 0.10091404 | 0.2368009 | 0.0000082 |
| R_CYTBO3 |  | 0 | 0.65428043 | 0 |
| R_CYTBO3 |  | 0 | 0.65428043 | 0 |
| R_FE2t4pp |  | 0.00175978 | 0 | 0 |
| R_NH4t4pp |  | 0.00175978 | 0 | 0 |
| R_COBALT2tpp |  | 2.25E-05 | 0 | 0 |
| R_PRAIS | Purine metabolism | 0.10091404 | 0.2368009 | 0.0000082 |
| R_KDOPS | Lipopolysaccharide biosynthesis | 0.00877004 | 0.02189475 | 0 |
| R_THDPS | Lysine biosynthesis | 0.0836 | 0.11342347 | 0.00054986 |
| R_IPMD | Valine, leucine and isoleucine biosynthesis | 0.10154127 | 0.13771979 | 0.00097949 |
| R_IPPS | Valine, leucine and isoleucine biosynthesis | 0.10154127 | 0.13771979 | 0.00097949 |
| R_PRFGS | Purine metabolism | 0.10091404 | 0.2368009 | 0.0000082 |
| R_ADSL2r | Purine metabolism | 0.10086378 | 0.2368009 | 0.0000082 |
| R_LEUTAi | Valine, leucine and isoleucine degradation | 0.10154127 | 0.13771979 | 0.00142719 |
| R_AHCi | Cysteine and methionine metabolism | -0.0545955 | 0.0001669 | 0.00112426 |
| R_ACLS | Valine, leucine and isoleucine biosynthesis | 0.1970439 | 0.2672494 | 0.00142719 |
| R_AIRCr | Purine metabolism | 0.10086378 | 0.2368009 | 0.0000082 |
| R_TRDR |  | 0.0614563 | 0.02432383 | 0.00093965 |
| R_TRPt2r |  | 0.01281135 | 0.01737595 | 0.00273325 |
| R_TYRt2r |  | 0 | 0.0421525 | 0 |
| R_U23GAAT | Lipopolysaccharide biosynthesis | 0.00877004 | 0.02189475 | 0 |
| R_UHGADA |  | 0.00877004 | 0.01189475 | 0.00254051 |
| R_FE2tex |  | 0 | 0.00457577 | 0 |
| R_UAGAAT |  | 0 | -521.3652 | 0 |
| R_HSK | Glycine, serine and threonine metabolism | 0 | 0.07754787 | 0 |
| R_UAGDP |  | 0.02129583 | 0.0288834 | 0.00058737 |
| R_THRS | Glycine, serine and threonine metabolism | 0 | 0.07754787 | 0 |
| R_ACGK | Arginine and proline metabolism | 0 | 0.09041866 | 0 |
| R_PItex |  | 0 | 0.29468875 | 0 |
| R_H2Otpp |  | -6.9163731 | -0.7552935 | 0.00173548 |
| R_UDCPDP | Peptidoglycan biosynthesis | 0 | 0.00849433 | 0 |
| R_H2Otex |  | -6.92 | -0.7575814 | 0.00260718 |
| R_H2Otex |  | -6.92 | -0.7575814 | 0.00260718 |
| R_AIRCr | Purine metabolism | 0.10086378 | 0.2368009 | 0.0000082 |
| R_UPP3MT | Porphyrin and chlorophyll metabolism | 0.0000503 | 6.82E-05 | 0.00031014 |
| R_UPP3S | Porphyrin and chlorophyll metabolism | 0.00010052 | 0.00013634 | 0.523 |
| R_UPPDC1 | Porphyrin and chlorophyll metabolism | 0.0000503 | 6.82E-05 | 0.00031014 |
| R_PRASCSi | Purine metabolism | 0.10086378 | 0.2368009 | 0.0000082 |
| R_USHD | Lipopolysaccharide biosynthesis | 0.00338502 | 0.00694737 | 0 |
| R_VALt2r |  | 0 | 6.935287 | 0 |
| R_VALt2rpp |  | 0 | -6.935287 | 0 |
| R_ARGSL | Alanine, aspartate and glutamate metabolism | 0 | 0.09041866 | 0 |
| R_H2Otex |  | -6.92 | -0.7575814 | 0.00179186 |
| R_ZNabc |  | 0.0000769 | 0.00010424 | 0.00185159 |
| R_SULabc |  | 0 | 0.00132605 | 0 |
| R_ARGSS | Alanine, aspartate and glutamate metabolism | 0 | 0.09041866 | 0 |
| R_Kt1 |  | 0.04399286 | 0.05966723 | 0.00014903 |
| R_HSDy | Glycine, serine and threonine metabolism | -0.100168 | 0 | 0 |
| R_NAt3_1 |  | 0 | 0.06757281 | 0 |
| R_DHDPS | Lysine biosynthesis | 0 | 0.11342347 | 0 |
| R_3OAS80 | Fatty acid biosynthesis | 0 | 0.08020871 | 0 |
| R_EAR100x | Fatty acid biosynthesis | 0 | 0.08020871 | 0 |
| R_EAR120x | Fatty acid biosynthesis | 0 | 0.08020871 | 0 |
| R_EAR60x | Fatty acid biosynthesis | 0 | 0.08020871 | 0 |
| R_KAS15 | Fatty acid biosynthesis | 0 | 0.08020871 | 0 |
| R_3HAD120 | Fatty acid biosynthesis | 0 | 0.08020871 | 0 |
| R_3HAD140 | Fatty acid biosynthesis | 0 | 0.06266863 | 0 |
| R_3OAR100 |  | 0.08020871 | 0 | 0 |
| R_3OAR120 |  | 0.08020871 | 0 | 0 |
| R_3OAR140 |  | 944.691287 | 0 | 0 |
| R_EAR140x | Fatty acid biosynthesis | 0 | 0.06266863 | 0 |
| R_3OAR160 |  | 0.02876497 | 0 | 0 |
| R_3OAR40 |  | 0.08020871 | 0 | 0 |
| R_KAS16 | Fatty acid biosynthesis | 0.02378949 | 0.08020871 | 0.0003317 |
| R_3OAR80 |  | 0.08020871 | 0 | 0 |
| R_3HAD160 | Fatty acid biosynthesis | 0.02876497 | 0.03020654 | 0.0003317 |
| R_3OAS160 | Fatty acid biosynthesis | 0.02876497 | 0.03020654 | 0.0003317 |
| R_EAR160x | Fatty acid biosynthesis | 0.02876497 | 0.03020654 | 0.0003317 |
| R_ACACT6r | Fatty acid degradation | 0 | -0.0237895 | 0 |
| R_ECOAH6 | Fatty acid degradation | 0 | -0.0237895 | 0 |
| R_FBP | Glycolysis | 0.1551087 | 0.32616306 | 0 |
| R_ASAD | Glycine, serine and threonine metabolism | -0.1837955 | 0 | 0 |
| R_DHAD2 | Valine, leucine and isoleucine biosynthesis | 0 | 0.18424661 | 0 |
| R_TKT2 | Pentose phospate pathway | -0.0825717 | 0 | 0 |
| R_PGK | Glycolysis | 0.42491764 | 0.84960989 | 0 |
| R_ASADi | Glycine, serine and threonine metabolism | 0 | 0.19097134 | 0 |
| R_CYTBO3 |  | 0 | 0.65428043 | 0 |
| R_ALATA_L |  | -0.1219885 | 0 | 0 |
| R_CYTBO3 |  | 0 | 0.65428043 | 0 |
| R_CYTK1 | Pyrimidine metabolism | 0 | 0.10136916 | 0 |
| R_AMMQT8_2 |  | 2.25E-05 | 0 | 0 |
| R_KARA2 | Valine, leucine and isoleucine biosynthesis | 0.06547981 | 0.27305647 | 0.00185159 |
| R_SULRi |  | 0.121 | 0.27305647 | 0.00254051 |
| R_ARGt2r |  | 0.03692088 | 0 | 0 |
| R_ARGt5r |  | 0.02974511 | 0 | 0 |
| R_METGL | Cysteine and methionine metabolism | 0.06547981 | 0.27305647 | 0.00185159 |
| R_ME1 | Pyruvate metabolism | 0 | 1.03972426 | 0 |
| R_ASPTA |  | -3.8865311 | 0 | 0 |
| R_CHORM |  | 0.03112944 | 0 | 0 |
| R_MG2tex |  | 0 | 45.3631428 | 0 |
| R_SULRi |  | 0.121 | 0.27305647 | 0.00254051 |
| R_CPPPGO2 | Porphyrin and chlorophyll metabolism | 5.03E-05 | 0 | 0 |
| R_CYTBD2pp |  | 0 | 1.19265984 | 0 |
| R_UMPK | Pyrimidine metabolism | 0 | 0.1155602 | 0 |
| R_CYSDSF | Thiamine metabolism | 0 | 0.24488592 | 0 |
| R_CYSS | Cysteine and methionine metabolism | 0.0208205 | 0.27305647 | 0.00037181 |
| R_CBPS | Pyrimidine metabolism | 0.07455501 | 0.19153716 | 0 |
| R_DHAD1 | Valine, leucine and isoleucine biosynthesis | 0 | 0.2672494 | 0 |
| R_SSALy | Alanine, aspartate and glutamate metabolism | 0 | 0.27111426 | 0 |
| R_SSCOARy | Alanine, aspartate and glutamate metabolism | 0 | 0.27111426 | 0 |
| R_DNTPPA |  | 0.00015078 | 0 | 0 |
| R_OGCOAAT | Fatty acid degradation | -0.0000225 | -3.06E-05 | 0.00014264 |
| R_KAS2 | Fatty acid biosynthesis | 0 | 0.02378949 | 0 |
| R_ACOAD6f | Fatty acid degradation | 0 | 0.02378949 | 0 |
| R_KAS7 | Fatty acid biosynthesis | 0 | 0.08499707 | 0 |
| R_EAR40x |  | 0.08020871 | 0 | 0 |
| R_ACCOAC | Fatty acid biosynthesis | 0.54392086 | 0.76150592 | 0.00010882 |
| R_EAR80x |  | 0.08020871 | 0 | 0 |
| R_FASC161ACP |  | 0.03390366 | 0 | 0 |
| R_FASC161ACP |  | 0.03390366 | 0 | 0 |
| R_NADH7 |  | 0 | 1.19265984 | 0 |
| R_ALCD2y |  | 0 | 2.443142 | 0 |
| R_FDMO_1 |  | 0.06547981 | 0 | 0 |
| R_FE2abc |  | 0.00337373 | 0 | 0 |
| R_PIuabcpp |  | 0 | 0.29468875 | 0 |
| R_FE3abcpp |  | 0.00175978 | 0 | 0 |
| R_FLDR |  | 0.01807558 | 0 | 0 |
| R_G3PAT160 |  | 0.01438248 | 0 | 0 |
| R_G3PAT161 |  | 0.01695183 | 0 | 0 |
| R_GART | Purine metabolism | 0 | 0.1368009 | 0 |
| R_GLNabc |  | 0.39958035 | 0 | 0 |
| R_ASPt2r |  | -2.5936874 | 0 | 0 |
| R_ACHBS | Valine, leucine and isoleucine biosynthesis | 0 | 0.27305647 | 0 |
| R_SERAT | Cysteine and methionine metabolism | 0 | 0.27305647 | 0 |
| R_UAGAAT | Lipopolysaccharide biosynthesis | 0.00877004 | 521.377093 | 0 |
| R_MICITDr | Citrate cycle | 0 | 0.18424661 | 0 |
| R_OIVD3 | Glycolysis | 0 | 0.18424661 | 0 |
| R_PRPPS | Pentose phospate pathway | 0 | 0.23861575 | 0 |
| R_MECDPDH2 |  | 0.00018031 | 0 | 0 |
| R_AMPTMETALA | Thiamine metabolism | 0 | 0.32010387 | 0 |
| R_MNabc |  | 0.00015574 | 0 | 0 |
| R_MTHFC | Glyoxylate and dicarboxylate metabolism | 0.20182807 | 0 | 0 |
| R_MTHFD | Glyoxylate and dicarboxylate metabolism | 0.20182807 | 0 | 0 |
| R_NADS2 | Nicotinate and nicotinamide metabolism | 0.00051342 | 0 | 0 |
| R_NAt3_15 |  | 0.35257719 | 0 | 0 |
| R_ACKr | Purine metabolism | 0 | 0.28495121 | 0 |
| R_AKGDa | Citrate cycle | 0 | 0.36244039 | 0 |
| R_AKGDb | Citrate cycle | 0 | 0.36244039 | 0 |
| R_DIPEPabc12 |  | 0 | 0.32010387 | 0 |
| R_PDHcr | Glycolysis | 0 | 0.36244039 | 0 |
| R_MCITL2 | Propanoate metabolism | 0 | 0.18424661 | 0 |
| R_PDX5PS | Vitamin B6 metabolism | 5.03E-05 | 0 | 0 |
| R_PDX5PS | Vitamin B6 metabolism | 5.03E-05 | 0 | 0 |
| R_PE160abcpp |  | 0.01035537 | 0 | 0 |
| R_HCO3E |  | 0.618 | 0.95304308 | 0.05273325 |
| R_MDH |  | -3.2520208 | 1.10812547 | 0.00058737 |
| R_PPNCL | Pantothenate and CoA biosynthesis | 0.00012982 | 0 | 0 |
| R_ASPT | Alanine, aspartate and glutamate metabolism | 0.77828288 | 1.28939565 | 0.06273325 |
| R_HCO3E |  | 0.618 | 0.95304308 | 0.00041269 |
| R_PDH | Glycolysis | 0 | 0.77273958 | 0 |
| R_ATPS4rpp |  | 12.9099563 | 25.0097621 | 0.00288154 |
| R_ATPS4rpp |  | 12.9099563 | 25.0097621 | 0.00288154 |
| R_DIPEPabc2 |  | 0 | 0.62353578 | 0 |
| R_DIPEPabc8 |  | 0 | 1.09552174 | 0 |
| R_AMPTALAGLN | Glutathione metabolism | 0 | 0.62353578 | 0 |
| R_SERt4 |  | 0.1007903 | 0 | 0 |
| R_MGt5 |  | -0.0019552 | 45.360491 | 0.00173548 |
| R_ATPS4rpp |  | 12.9099563 | 25.0097621 | 0.00061388 |
| R_ATPS4rpp |  | 12.9099563 | 25.0097621 | 0.00061388 |
| R_THIORDXi |  | 5.03E-05 | 0 | 0 |
| R_ASNN | Alanine, aspartate and glutamate metabolism | 0 | 1.02183526 | 0 |
| R_THRt4 |  | 0.60436407 | 0 | 0 |
| R_AMPTGLYASN | Thiamine metabolism | 0 | 1.09552174 | 0 |
| R_PPS | Pyruvate metabolism | 0 | 0.54040797 | 0 |
| R_TYRTA |  | -0.0311294 | 0 | 0 |
| R_UDCPDP |  | 0.0032629 | 0.00849433 | 0.00179186 |
| R_ADK1 | Purine metabolism | 0.44322454 | 1.31676398 | 0 |
| R_SUCOAS | Citrate cycle | -3.5690164 | 0 | 0 |

**Table S3.** The calculated metabolic fluxes of AB5075 during infection at 2 and 4 h.

a. Significantly changed fluxes in fatty acid biosynthesis and degradation pathways at 2 and 4 h.

| **Reaction ID** | **Subsystem** | **Flux (2 h)** | **Flux (4 h)** |
| --- | --- | --- | --- |
| R_3HAD100 | Fatty acid biosynthesis | 0 | 0.08020871 |
| R_3HAD40 | Fatty acid biosynthesis | 0 | 0.08020871 |
| R_3HAD60 | Fatty acid biosynthesis | 0 | 0.08020871 |
| R_3HAD80 | Fatty acid biosynthesis | 0 | 0.08020871 |
| R_3OAR60 | Fatty acid biosynthesis | 0 | 0.08020871 |
| R_3OAS100 | Fatty acid biosynthesis | 0 | 0.08020871 |
| R_3OAS120 | Fatty acid biosynthesis | 0 | 0.08020871 |
| R_3OAS60 | Fatty acid biosynthesis | 0 | 0.08020871 |
| R_3OAS80 | Fatty acid biosynthesis | 0 | 0.08020871 |
| R_EAR100x | Fatty acid biosynthesis | 0 | 0.08020871 |
| R_EAR120x | Fatty acid biosynthesis | 0 | 0.08020871 |
| R_EAR60x | Fatty acid biosynthesis | 0 | 0.08020871 |
| R_KAS15 | Fatty acid biosynthesis | 0 | 0.08020871 |
| R_3HAD120 | Fatty acid biosynthesis | 0 | 0.08020871 |
| R_3HAD140 | Fatty acid biosynthesis | 0 | 0.06266863 |
| R_EAR140x | Fatty acid biosynthesis | 0 | 0.06266863 |
| R_KAS16 | Fatty acid biosynthesis | 0.02378949 | 0.08020871 |
| R_3HAD160 | Fatty acid biosynthesis | 0.02876497 | 0.03020654 |
| R_3OAS160 | Fatty acid biosynthesis | 0.02876497 | 0.03020654 |
| R_EAR160x | Fatty acid biosynthesis | 0.02876497 | 0.03020654 |
| R_ACACT6r | Fatty acid degradation | 0 | -0.0237895 |
| R_ECOAH6 | Fatty acid degradation | 0 | -0.0237895 |
| R_OGCOAAT | Fatty acid degradation | -0.0000225 | -3.06E-05 |
| R_KAS2 | Fatty acid biosynthesis | 0 | 0.02378949 |
| R_ACOAD6f | Fatty acid degradation | 0 | 0.02378949 |
| R_KAS7 | Fatty acid biosynthesis | 0 | 0.08499707 |
| R_ACCOAC | Fatty acid biosynthesis | 0.54392086 | 0.76150592 |

b. Significantly changed amino acid metabolism at 2 and 4 h.

| **Reaction ID** | **Subsystem** | **Flux (2 h)** | **Flux (4 h)** |
| --- | --- | --- | --- |
| R_ASPO5 | Alanine, aspartate and glutamate metabolism | 0.00051342 | 0.00069635 |
| R_GF6PTA | Alanine, aspartate and glutamate metabolism | 0.02129583 | 0.0288834 |
| R_ARGSL | Alanine, aspartate and glutamate metabolism | 0 | 0.09041866 |
| R_ARGSS | Alanine, aspartate and glutamate metabolism | 0 | 0.09041866 |
| R_SSALy | Alanine, aspartate and glutamate metabolism | 0 | 0.27111426 |
| R_SSCOARy | Alanine, aspartate and glutamate metabolism | 0 | 0.27111426 |
| R_ASNN | Alanine, aspartate and glutamate metabolism | 0 | 1.02183526 |
| R_ACOTA | Arginine and proline metabolism | 0 | -0.0904187 |
| R_AGPR | Arginine and proline metabolism | 0 | -0.0904187 |
| R_ACGK | Arginine and proline metabolism | 0 | 0.09041866 |
| R_ASP1DC | beta-Alanine metabolism | 0.00012982 | 0.00017607 |
| R_PANTS | beta-Alanine metabolism | 0.00012982 | 0.00017607 |
| R_METS | Cysteine and methionine metabolism | 0.10029108 | 0 |
| R_AHSERL2 | Cysteine and methionine metabolism | 0.10016802 | 0 |
| R_HSERTA | Cysteine and methionine metabolism | 0.10016802 | 0 |
| R_AHCYSNS | Cysteine and methionine metabolism | 0.05471853 | 0 |
| R_SERAT | Cysteine and methionine metabolism | 0.0208205 | 0 |
| R_DLYSPYRAT | Cysteine and methionine metabolism | 0.00939434 | 0 |
| R_5DOAN | Cysteine and methionine metabolism | 0.00010052 | 0 |
| R_METAT | Cysteine and methionine metabolism | 0.00027384 | 0.00023507 |
| R_METS_1 | Cysteine and methionine metabolism | 0 | 0.0001669 |
| R_DLYSOXGAT | Cysteine and methionine metabolism | -0.0093943 | 0 |
| R_AHCi | Cysteine and methionine metabolism | -0.0545955 | 0.0001669 |
| R_METGL | Cysteine and methionine metabolism | 0.06547981 | 0.27305647 |
| R_CYSS | Cysteine and methionine metabolism | 0.0208205 | 0.27305647 |
| R_SERAT | Cysteine and methionine metabolism | 0 | 0.27305647 |
| R_ALAALAr | D-Alanine metabolism | 0.0032629 | 0.00849433 |
| R_ALAR | D-Alanine metabolism | 0 | 0.01274149 |
| R_UAMAGS | D-Glutamine and D-glutamate metabolism | 0.0032629 | 0.00849433 |
| R_UAMAS | D-Glutamine and D-glutamate metabolism | 0.0032629 | 0.00849433 |
| R_GLUR | D-Glutamine and D-glutamate metabolism | -0.0156572 | -0.0084943 |
| R_THRAi | Glycine, serine and threonine metabolism | 0.54718776 | 0 |
| R_GHMT2r | Glycine, serine and threonine metabolism | 0 | -0.381519 |
| R_HSDx | Glycine, serine and threonine metabolism | 0 | -0.0775479 |
| R_OHPBAT | Glycine, serine and threonine metabolism | 0.0000503 | 6.82E-05 |
| R_PSSA160 | Glycine, serine and threonine metabolism | 0.01438248 | 0.01950687 |
| R_PSSA161 | Glycine, serine and threonine metabolism | 0.01695183 | 0.02299166 |
| R_ASPK | Glycine, serine and threonine metabolism | 0.18379554 | 0.19097134 |
| R_HSK | Glycine, serine and threonine metabolism | 0 | 0.07754787 |
| R_THRS | Glycine, serine and threonine metabolism | 0 | 0.07754787 |
| R_HSDy | Glycine, serine and threonine metabolism | -0.100168 | 0 |
| R_ASAD | Glycine, serine and threonine metabolism | -0.1837955 | 0 |
| R_ASADi | Glycine, serine and threonine metabolism | 0 | 0.19097134 |
| R_DHDPS | Lysine biosynthesis | 0.08362751 | 0 |
| R_UAAGDS | Lysine biosynthesis | 0.0032629 | 0.00849433 |
| R_UGMDDS | Lysine biosynthesis | 0.0032629 | 0.00849433 |
| R_DAPDC | Lysine biosynthesis | 0.07736462 | 0.10492914 |
| R_DAPE | Lysine biosynthesis | 0.08362751 | 0.11342347 |
| R_DHDPRy | Lysine biosynthesis | 0.08362751 | 0.11342347 |
| R_SDPDS | Lysine biosynthesis | 0.08362751 | 0.11342347 |
| R_THDPS | Lysine biosynthesis | 0.0836 | 0.11342347 |
| R_DHDPS | Lysine biosynthesis | 0 | 0.11342347 |
| R_DHQTi | Phenylalanine, tyrosine and tryptophan biosynthesis | 0.03128022 | 0 |
| R_PPND | Phenylalanine, tyrosine and tryptophan biosynthesis | 0.03112944 | 0 |
| R_CHORS | Phenylalanine, tyrosine and tryptophan biosynthesis | 0.03128022 | 0.0002045 |
| R_DDPA | Phenylalanine, tyrosine and tryptophan biosynthesis | 0.03128022 | 0.0002045 |
| R_DHQS | Phenylalanine, tyrosine and tryptophan biosynthesis | 0.03128022 | 0.0002045 |
| R_PSCVT | Phenylalanine, tyrosine and tryptophan biosynthesis | 0.03128022 | 0.0002045 |
| R_SHK3Dr | Phenylalanine, tyrosine and tryptophan biosynthesis | 0.03128022 | 0.0002045 |
| R_SHKK | Phenylalanine, tyrosine and tryptophan biosynthesis | 0.03128022 | 0.0002045 |
| R_DHQTi | Phenylalanine, tyrosine and tryptophan biosynthesis | 0 | 0.0002045 |
| R_DHAD1 | Valine, leucine and isoleucine biosynthesis | 0.1970439 | 0 |
| R_KARA1 | Valine, leucine and isoleucine biosynthesis | -0.1970439 | -0.2672494 |
| R_ACHBS | Valine, leucine and isoleucine biosynthesis | 0.06547981 | 0 |
| R_ACHBS | Valine, leucine and isoleucine biosynthesis | 0.06547981 | 0 |
| R_DHAD3 | Valine, leucine and isoleucine biosynthesis | 0.06547981 | 0.08880985 |
| R_DHAD2 | Valine, leucine and isoleucine biosynthesis | 0 | 0.18424661 |
| R_KARA2 | Valine, leucine and isoleucine biosynthesis | 0.06547981 | 0.27305647 |
| R_DHAD1 | Valine, leucine and isoleucine biosynthesis | 0 | 0.2672494 |
| R_ACHBS | Valine, leucine and isoleucine biosynthesis | 0 | 0.27305647 |
| R_VALTA | Valine, leucine and isoleucine degradation | -0.0953728 | -0.1293535 |
